# The global prevalence of female genital mutilation/cutting: A systematic review and meta-analysis of national, regional, facility, and school-based studies

**DOI:** 10.1371/journal.pmed.1004061

**Published:** 2022-09-01

**Authors:** Leen Farouki, Zeinab El-Dirani, Sawsan Abdulrahim, Christelle Akl, Chaza Akik, Stephen J. McCall

**Affiliations:** 1 Center for Research on Population and Health, Faculty of Health Sciences, American University of Beirut, Beirut, Lebanon; 2 Department of Health Promotion and Community Health, Faculty of Health Sciences, American University of Beirut, Beirut, Lebanon; Cornell University Joan and Sanford I Weill Medical College, UNITED STATES

## Abstract

**Background:**

Female genital mutilation/cutting (FGM/C) is a nonmedical procedure entailing the modification of the external female genitalia. A description of the prevalence and distribution of FGM/C allows the tracking of progress toward ending FGM/C by 2030 (Sustainable Development Goal (SDG): target 5.3). This systematic review aimed to examine FGM/C prevalence and types, by World Health Organization (WHO) region and country.

**Methods and findings:**

A systematic search using Medical Subject Headings (MeSH) and keywords from 2009 to March 24, 2022 was undertaken in MEDLINE, PubMED, PsycINFO, Web of Science, and Embase to identify studies presenting FGM/C prevalence. Abstract and full-text screening, quality assessment, and data extraction were undertaken by 2 reviewers. Only nationally representative studies were included in the meta-analysis. Pooled FGM/C prevalence was estimated by random-effects meta-analysis using generalized linear mixed models (GLMMs). FGM/C prevalence with 95% confidence intervals (CIs), prediction intervals (PIs), and FGM/C type were presented separately by women aged 15 to 49 years and girls aged 0 to 14 years.

A total of 163 studies met the inclusion criteria and 30 were included in the meta-analysis, of which 23 were from the WHO African Region (AFR), 6 from the Eastern Mediterranean Region (EMR), and 1 from the South East Asian Region (SEAR). These studies included data from 406,068 women across 30 countries and 296,267 girls across 25 countries; the pooled prevalence estimate of FGM/C among women aged 15 to 49 years was 36.9% (95% CI: 19.6% to 58.3%; PI: 0.4% to 99.0%), and 8.27% (95% CI: 3.7% to 17.3%; PI: 0.1% to 89.3%) among girls aged 0 to 14 years. Among included countries, this gave a total estimated prevalence of 84,650,032 women (95% CI: 45,009,041 to 133,834,224) and 13,734,845 girls with FGM/C (95% CI: 6,211,405 to 28,731,901). Somalia had the highest FGM/C prevalence among women (99.2%), and Mali had the highest among girls (72.7%). The most common type of FGM/C among women was “flesh removed” (Type I or II) in 19 countries. Among girls, “not sewn closed” (Type I, II, or IV) and “flesh removed” (Type I or II) were the most common types in 8 countries, respectively. Among repeated nationally representative studies, FGM/C decreased for both women and girls in 26 countries. The main limitation of the study methodology is that estimates were based on available published data, which may not reflect the actual global prevalence of FGM/C.

**Conclusions:**

In this study, we observed large variation in FGM/C prevalence between countries, and the prevalence appears to be declining in many countries, which is encouraging as it minimizes physical and physiological harm for a future generation of women. This prevalence estimate is lower than the actual global prevalence of FGM/C due to data gaps, noncomparable denominators, and unavailable surveys. Yet, considerable policy and community-level interventions are required in many countries to meet the SDG target 5.3.

**Trial registration:**

Registration: CRD42020186937.

## Abstract 2

Leen Farouki and colleagues discuss the global prevalence and distribution of female genital mutilation/cutting by World Health Organization region and country.

## Author summary

**Why was this study done?:**

- Female genital mutilation/cutting (FGM/C) is an extreme form of gender inequality that violates women’s and girls’ human rights, and the practice has lifelong health and economic consequences for women and girls.
- Previous studies on prevalence of FGM/C have used repeated nationally representative cross-sectional studies and found that FGM/C is decreasing in many countries.
- This study aimed to provide a baseline prevalence estimate and to understand the data gaps in prevalence required for tracking progress toward the Sustainable Development Goal (SDG) target 5.3.

**What did the researchers do and find?:**

- This was a systematic review and meta-analysis of all available studies on FGM/C, and it provided a thorough overview of studies published on FGM/C prevalence at a national, subregional, school, facility, and community level.
- Approximately 100 million girls and women of reproductive age have experienced FGM/C across 30 countries in 3 WHO regions, with a prevalence of 37% in women and 8% among girls.
- There were large differences between regions and countries, where some countries practiced FGM/C universally, and FGM/C appeared to be decreasing in 26 countries for both women and girls.

**What do these findings mean?:**

- Current findings imply that progress toward SDG 5.3 is attainable in some countries, but much work is required in others, including Egypt, Somalia, Sudan, Indonesia, Guinea, and Mali.
- Evaluation of structural or community level policies and interventions in countries that had a decline in FGM/C will be beneficial for countries that have a high prevalence of FGM/C.
- The prevalence estimate of this study is accurate of the included countries but is an underestimate of the global prevalence due to gaps in available data across the world, which are important to resolve to understand actual progress toward SDG 5.3.

## Introduction

Female genital mutilation/cutting (FGM/C), also referred to as female circumcision, is a nonmedical procedure that entails the total or partial removal of external female genitalia and other injuries to the female genital organs [1]. The United Nations Sustainable Development Goal (SDG) target 5.3 on gender equality refers to FGM/C as a harmful traditional practice and calls for ending the practice by 2030.

While the exact global prevalence of FGM/C is unknown, estimates of FGM/C range from 100 to 140 million women and girls in the African and the Middle Eastern Region [2,3], and UNICEF estimates the global prevalence to be over 200 million women and girls living with FGM/C [1]. Nationally representative data show that there is a decline in the prevalence of FMG/C, but this is not universal across countries [1,4,5]. FGM/C persists due to religious, social, and cultural factors [6]. It is commonly believed to create better marriage prospects because of beliefs related to morality, hygiene, and aesthetics; FGM/C is also believed to curb sexual urges and maintain virginity [7]. However, the procedure has no health benefits and has resulted in negative health outcomes, including menstrual difficulties, infertility, urinary problems, mental health problems, pregnancy complications, severe pain, infection, septicemia, and even death [8–10]. FGM/C is also an economic burden throughout the life course for women and girls [11].

FGM/C is most often performed on girls between infancy and adolescence and has been classified into 4 types [12]. Type I (clitoridectomy) involves the partial or total removal of the prepuce and/or the clitoral gland. Type II involves the partial or total removal of the labia minora and clitoral glans without the excision of the labia majora. Type III (infibulation) involves narrowing the vaginal canal by modifying the labia majora and minora and may also include the removal of the clitoral glans. Type IV involves any other nonmedical, harmful procedure, such as cauterization, pricking, and scraping [7]. Risks differ by type; the most severe type, Type III, has serious obstetric risks including infant resuscitation, stillbirth, and neonatal death; while Types I and II carry risks of cesarean section and postpartum bleeding [13].

An important aspect of the SDGs is to track progress on ending harmful traditional practices such as FGM/C. However, to our knowledge, there is no comprehensive review in the literature that provides estimates of FGM/C globally, by World Health Organization (WHO) region, or specific countries, which can be used to track improvements toward SDG 5.3. A review of the prevalence of FGM/C will support efforts to understand the global burden of FGM/C and inform adequate prevention and intervention efforts, and local and international policies. A review of the types of FGM/C will contribute similarly by tracking the severity of the procedure. This systematic review and meta-analysis aimed to examine (1) the prevalence of FGM/C and (2) the proportion of the different types of FGM/C, among girls aged 0 to 14 years and women aged 15 to 49 years old by country and WHO region.

## Methods

### Search strategy and study selection

In this systematic review and meta-analysis of FGM/C prevalence, separate searches were conducted in the following databases: MEDLINE, PubMED, PsycINFO, Web of Science, and Embase. Hand searches of the gray literature were conducted through searches of reports from international nongovernmental organizations, including UNFPA and UNICEF among others, and other Google searches. Hand searches of the bibliographies of relevant systematic reviews were also conducted. Together, these databases provide international and interdisciplinary publications. The search strategy (S1 Table) was adapted to the format of each database. To present up-to-date data that can be used as a baseline to monitor progress on SDG 5.3 over the last decade, the search was limited to include publications from 2009 until 2020. The search was updated to include publications from 2009 until 2022. The last search in all databases was conducted on March 24, 2022. For nationally representative studies, the hand searches were conducted to include studies prior to 2009 in a post hoc analysis to present FGM/C prevalence across time. The MeSH term for FGM/C was used when possible; otherwise, keywords were used, including “Female Genital Mutilation,” “Female Genital Alteration,” “Female Circumcision,” and “Female Genital Cutting”. No language restrictions were imposed. The references were imported from each database into EndNote then into systematic review software DistillerSR and duplicates were removed [14].

### Study protocol, registration, and reporting

The reporting of this study was based on the Preferred Reporting Items for Systematic Review (PRISMA) reporting guidelines (S1 PRISMA Checklist) [15,16]. The prospectively written study protocol (S1 Study Protocol) was registered with PROSPERO, number CRD42020186937 [17].

### Inclusion and exclusion criteria

This systematic review and meta-analysis were part of a larger project on FGM/C prevalence and its determinants [6,17]. Cohort or cross-sectional studies that reported on FGM/C prevalence at the national level, using representative samples or population-based methods, were included in the systematic review and meta-analysis. Subregional, facility, community, and school-based studies and studies that used non-population-based methods or non-probability sampling designs, including cross-sectional, cohort designs, were included in the systematic review but not in the meta-analysis. Furthermore, case-series in migrant populations outside of countries that practice FGM/C were included to understand the scope of the literature on FGM/C in these countries.

Studies were excluded if they (i) only reported on health outcomes, determinants, the attitudes and knowledge of healthcare providers, economic effects, or perceptions of FGM/C; (ii) only used qualitative methods; (iii) were systematic reviews (except for referencing); or (iv) were policy reports, conference proceedings, or letters to the editor. If numerous journal articles used the same data source, e.g., secondary data analysis of international surveys, only the original report was included. Other than nationally representative studies, if the same data source completed multiple studies in a given country across time, then the most recent was included. The supporting information contains further details on the included and excluded studies (S1 Text).

### Study screening

Titles and abstracts were screened independently by 2 reviewers. Articles selected for full-text review were also screened by 2 reviewers, independently and in duplicates. The reasons for exclusion at both the abstract and full-text stages were recorded. Disagreements between the 2 reviewers were resolved by discussion and consulting a third reviewer who verified the eligibility of all included studies. The supporting information contains further details on the screening process (S2 Table).

### Data extraction and quality assessment

Data were extracted from included articles using a structured data extraction form, uploaded onto DistillerSR. Data were extracted by 1 reviewer and verified by a second reviewer; disagreements were resolved by a third reviewer. Data included in the final tables were verified against the original publication by a further reviewer. Items extracted from studies included study characteristics, sampling methods, design, host country and country of origin, ethnicity, age, age at FGM/C, location of procedure, performer of FGM/C, FGM/C prevalence, and proportion of different FGM/C types. The FGM/C prevalence in each included study was extracted as a proportion or calculated from the numbers presented. All data items were extracted from the most recent nationally representative studies (e.g., MICS or DHS), while only prevalence estimates were extracted from the older nationally representative studies for a post hoc analysis. Studies were assessed for risk of bias independently by 2 reviewers using an adapted tool by Hoy and colleagues, which is specific to prevalence studies [18]. This tool includes 9 items that collectively assess the selection bias, representativeness of the sample, validity of the tool, and appropriateness of the estimate. Each item was scored as low or high risk of bias, and each paper was given an overall score rated as low, moderate, or high risk of bias.

### Data analysis

Because the literature fell into certain categories, namely nationally representative, subregional, and non-probability samples, data in the present study were grouped similarly. Prevalence estimates from the different studies were grouped by country, WHO region, and study design. Pooled estimates of FGM/C prevalence were only presented from studies with representative samples or population-based methods at a national level, and the most recent survey was used in the meta-analysis. Prevalence estimates were presented separately for women aged 15 to 49 years old and girls aged 0 to 14 years old as most studies collected data for women and girls separately as defined by these age groups, and it was considered inappropriate to pool these groups together due to a cohort effect [4,5]. Studies that estimated FGM/C among girls using the number of women with at least 1 daughter with FGM/C were excluded from the meta-analysis because this does not provide an estimate of prevalence among all girls aged 0 to 14 years old. The denominator of FGM/C type was the total number of women and girls with FGM/C, respectively. In addition, a post hoc summary of prevalence estimates of FGM/C for each country was presented across time for both women and girls.

For the meta-analysis, heterogeneity between studies is usually assessed using the *I*^***2***^ statistic [19]. Although high values of *I*^***2***^ are common in meta-analysis for prevalence studies, prediction intervals are recommended to be presented as a measure of heterogeneity [20]. The prediction interval is the range where a proportion from a future study would be expected to be located within if this study was randomly selected from the same group of studies included in the meta-analysis [21]. In addition, τ^2^ values were also presented as a measure of the variance of effect sizes among studies [22]. Using data extracted from survey reports, a random-effects meta-analysis was conducted to produce a pooled prevalence across all nationally representative studies and across each WHO region. The random-effects meta-analysis of the pooled prevalence, 95% confidence intervals (CIs), and prediction intervals (PIs) were estimated using generalized linear mixed models (GLMMs) [23] through the “metaprop” command within the Meta package, version 4.15–1 [24]. Funnel plots were constructed to inspect visual asymmetry using the funnelR package, version 0.1.0, which was developed for proportion data (S1 and S2 Figs, S2 Text) [25]. To provide the total number of girls (0 to 14 years old) and women (15 to 49 years old) with FGM/C, the pooled prevalence estimate was extrapolated against the age-specific population total in 2020, which only included countries that were included in the meta-analysis, using the UN Population Division [26]. All statistical analyses were conducted using R version 4.1.2.

### Protocol amendments

The protocol was amended to include studies in any language and to specify the disaggregation by age group (S1 Study Protocol). Other than studies involving migrants, case series and case–control studies were excluded as prevalence cannot be calculated. A data-driven analysis was conducted to present prevalence of FMG/C across time from national surveys. A GLMM meta-analysis was used rather than a Freeman–Tukey transformation due to the limitations of the latter approach [23]. We also provided prediction intervals due to recent methodological recommendations, and we presented the total number of women and girls with FGM/C to allow comparison with other global estimates [20].

### Ethical approval and role of the funding source

This was a systematic review of published studies, so no ethical approval was required. There was no funding source for this study.

## Results

**Fig 1:**
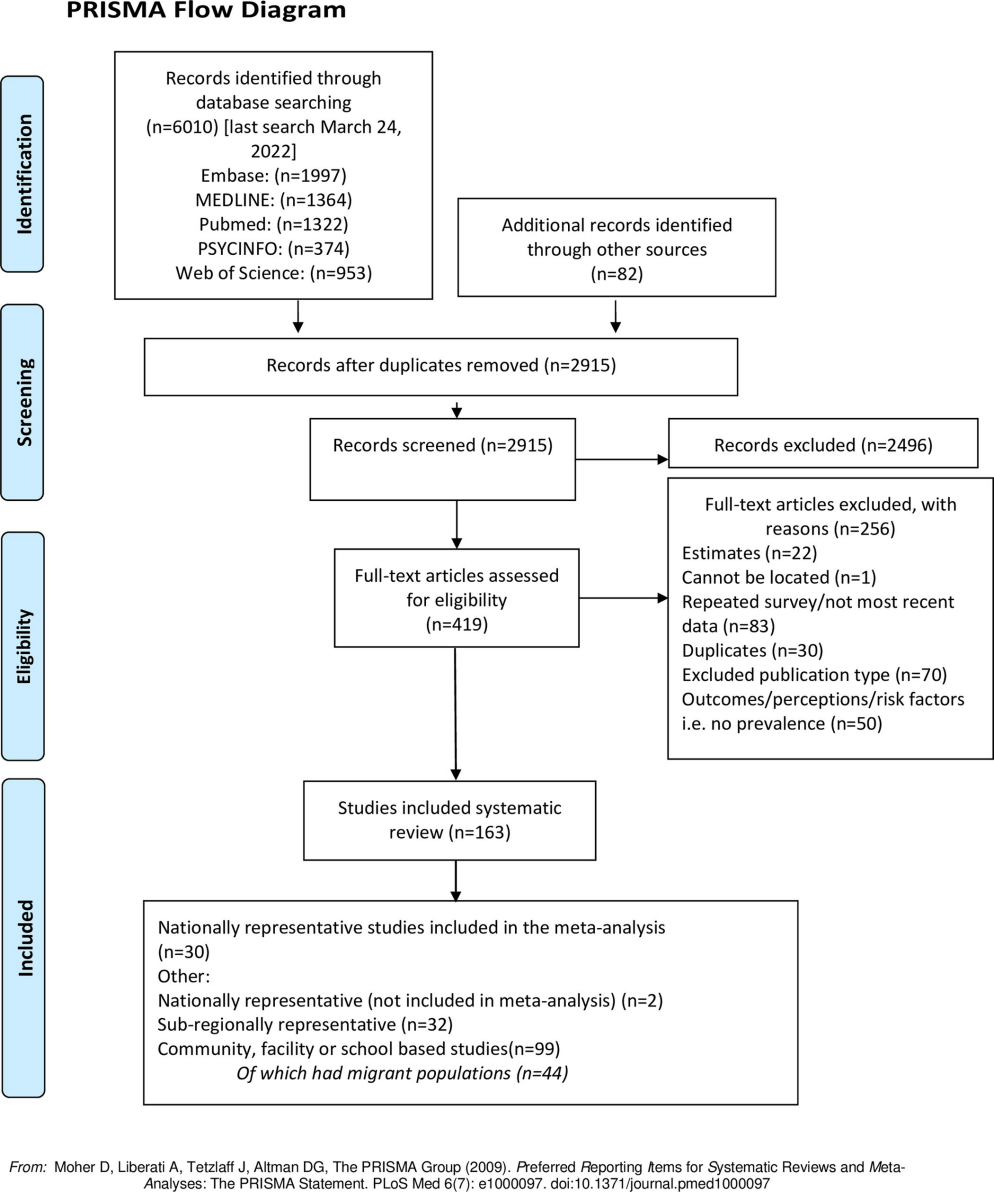
PRISMA flow diagram of study selection.

Out of 2,915 records retrieved from database and hand searches, 419 publications were assessed under full-text review. Of these, a total of 163 were included in the systematic review: 30 nationally representative studies were included in the meta-analysis of FGM/C prevalence, and 2 were included in the systematic review but not in the meta-analysis; 32 subregional studies; and 99 community, school, or facility-based studies including 44 on migrant populations (Fig 1). The Indonesia RISKESDAS survey [27] was not included in the meta-analysis because it did not provide the sample size, and the Pew Research Center [28] and Yemen DHS surveys [29] were not included in the meta-analysis of FGM/C prevalence of girls as these surveys did not have comparable denominators.

### Nationally representative studies

Of the 32 nationally representative studies, 16 used data from Demographic and Health Surveys (DHS), 10 used data from Multiple Indicator Cluster Surveys (MICS), and 6 used other population-based surveys (S3 Table). Furthermore, 23 represent the African Region (AFR) [30–52], 6 represent the Eastern Mediterranean Region (EMR) [29,53–57], 2 represent the South East Asian Region (SEAR) [27,58], and 1 represented both EMR and AFR [28]. All national studies reported FGM/C prevalence among the total number of women and girls in surveyed households, except surveys from Liberia (reported on women who have heard of FGM/C) [42], surveys from Zambia [52], Niger [46], and Uganda [51] (reported only on women), and surveys from Yemen [29] and the Pew Research Center [28] (asked women whether at least one of their daughters had FGM/C). Apart from that of the Pew Research Center, all studies had a low risk of bias and used a cross-sectional design with multistage cluster sampling. The Pew Research Center survey had a moderate risk of bias, a cross-sectional design, and used stratified random sampling [28].

**Fig 2:**
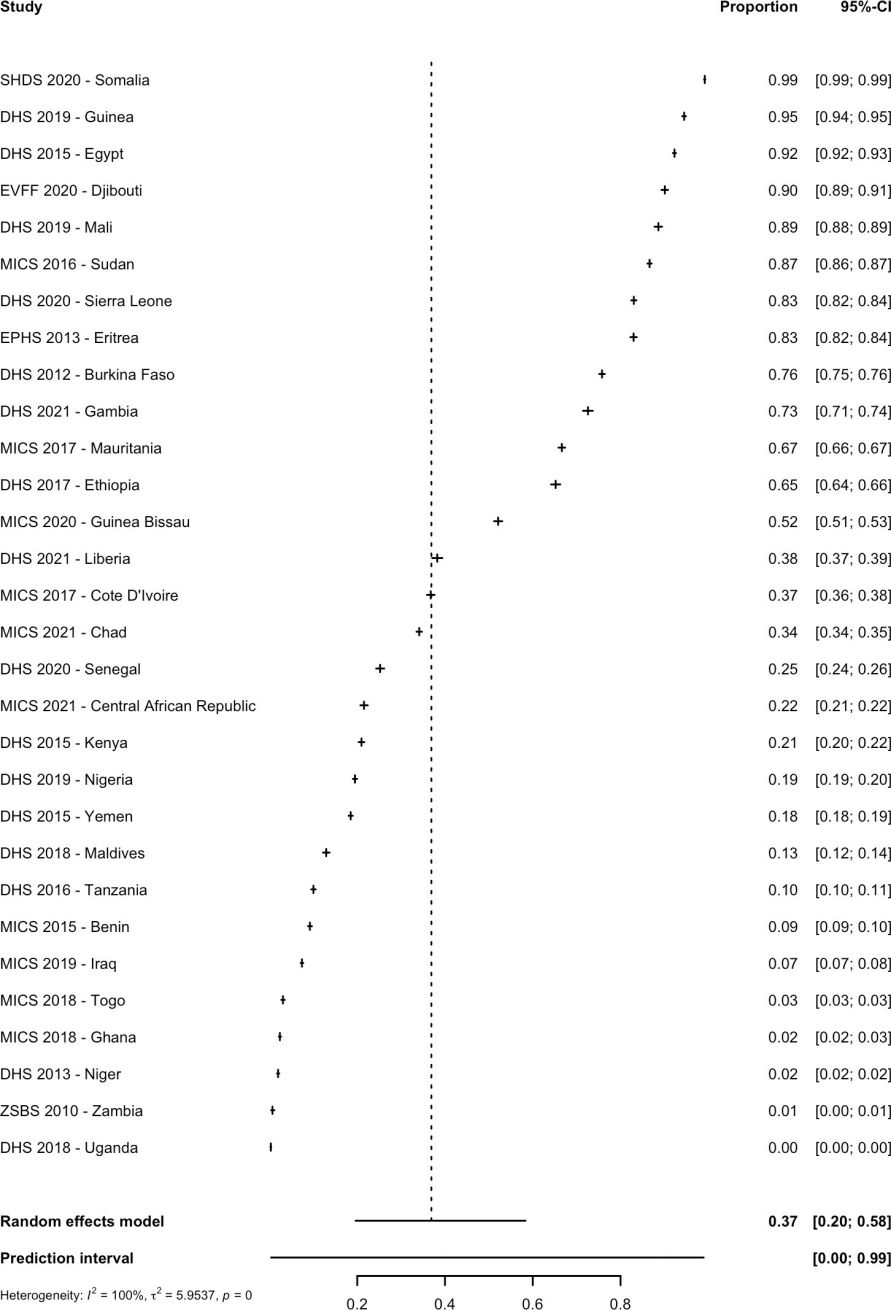
Pooled prevalence of FGM/C among women in 30 countries. There were 32 studies included in the systematic review as nationally representative studies; however, the Pew Research Study [28] and the Indonesia RISKESDAS survey [27] did not include women, thus they were not included in this analysis. The year indicates the date of publication. CI, Confidence Interval; DHS, Demographic and Health Surveys; EPHS, Eritrea Population and Health Survey; EVFF, L’enquête nationale sur les violences faites aux femmes (National survey on violence against women); MICS, Multiple Indicator Cluster Surveys; SHDS, Somali Health and Demographic Survey; ZSBZ, Zambia Sexual Behaviour Survey.

**Fig 3:**
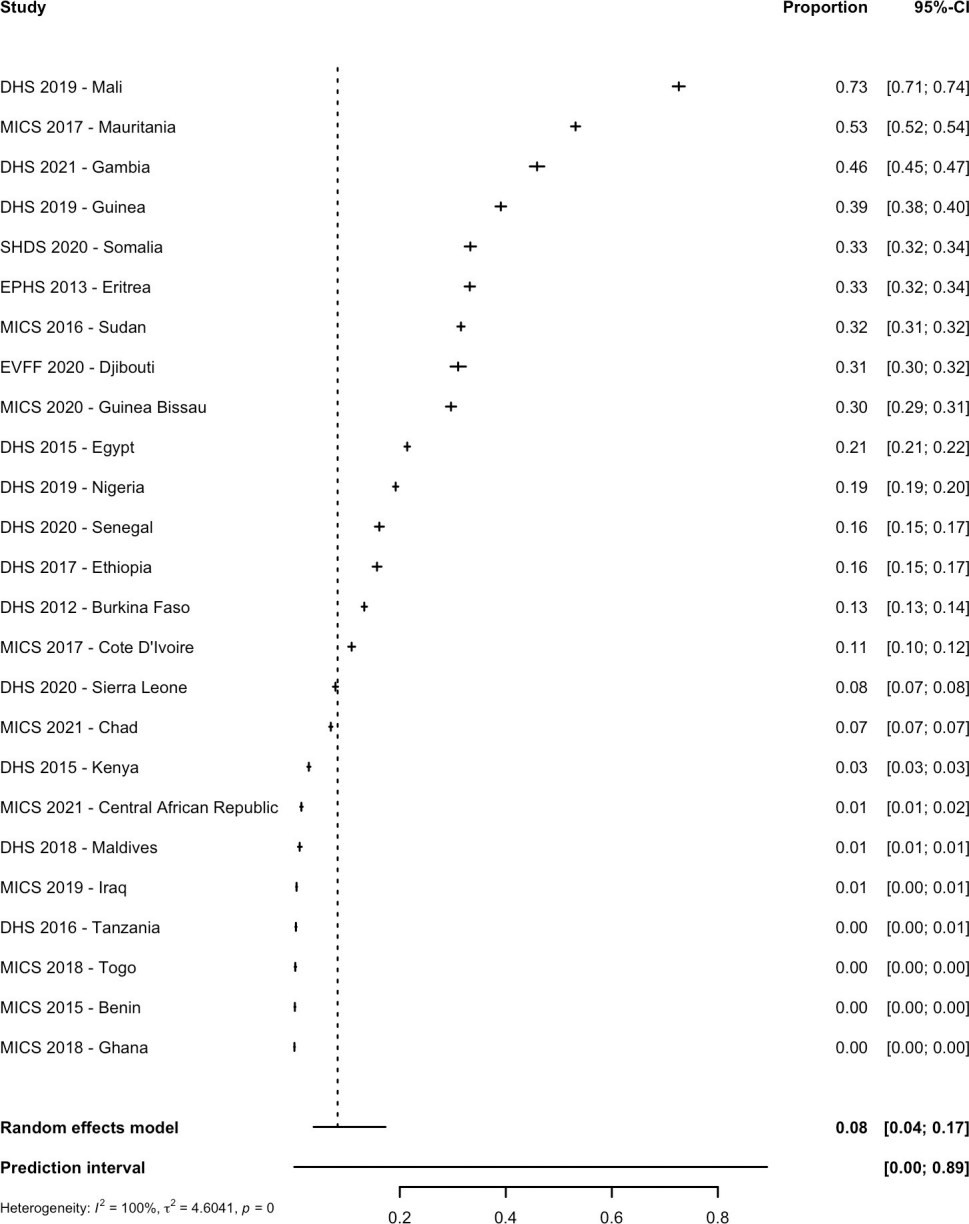
Pooled prevalence of FGM/C among girls in 25 countries. There were 32 studies included in the systematic review as nationally representative studies; however, surveys from Zambia [52], Liberia [42], Niger [46], and Uganda [51] did not include girls, and the Pew Research Study [28] and Yemen [29] only included women who reported on at least 1 daughter in their household who has had FGM/C, and the Indonesia RISKESDAS survey [27] did not report a sample size, thus they were not included in this analysis. The year indicates the date of publication. CI, confidence interval; DHS, Demographic and Health Surveys; EPHS, Eritrea Population and Health Survey; EVFF, L’enquête nationale sur les violences faites aux femmes (National survey on violence against women); MICS, Multiple Indicator Cluster Surveys; SDHS, Somali Health and Demographic Survey.

The 30 nationally representative studies included in the meta-analysis provided data on women in 30 countries and data on girls in 25 countries. Out of a total of 406,068 women aged 15 to 49 years in 30 countries, 168,997 women had FGM/C representing a pooled prevalence of 36.9% (CI: 19.6% to 58.3%; PI: 0.4% to 99.0%; τ^2^ = 6.0) (Table 1 and Fig 2). Prevalence estimates varied considerably by country and ranged from 99.2% in Somalia [56] to 0.3% in Uganda [51]. Out of a total of 296,267 girls aged 0 to 14 years in 25 countries, 50,686 girls had FGM/C, and this gave a pooled prevalence of 8.3% (95% CI: 3.7% to 17.3%; PI: 0.1% to 89.3%; τ^2^ = 4.6). The country level prevalence ranged between 72.7% in Mali [43] and 0.1% in Ghana [38] (Table 1 and Fig 3). Among included countries, the total estimated prevalence was 84,650,032 women (95% CI: 45,009,041 to 133,834,224) and 13,734,845 girls with FGM/C (95% CI: 6,211,405 to 28,731,901) (Table 1).

**Table 1:** Prevalence of FGM/C in women and girls in nationally representative studies.

			**Women 15–49 years**			**Girls 0–14 years^¶^**		
**WHO Region**	**Country, Survey^§^**	**Year^§§^**	**FGM/C, %**	**Total number with FGM/C**	**Sample Size**	**FGM/C, %**	**Total number with FGM/C**	**Sample Size**
**AFR**	Benin, MICS [30]	2014	9.2	1,457	15,815	0.2	20	9,902
	Botswana, Pew study^†^ [28]	2010				5	20*	399
	Burkina Faso, DHS [31]	2010	75.8	12,949	17,087	13.3	2,319	17,434
	Cameroon, Pew study^†^ [28]	2010				1	8*	755
	Central African Republic, MICS [32]	2018–2019	21.6	1,983	9,202	1.4	139	9,704
	Chad, MICS [33]	2019	34.1	7,698	22,561	7	1,838	26,303
	Chad, Pew study^†^ [28]	2010				39	304*	779
	Cote D’Ivoire, MICS [34]	2016	36.7	4,329	11,780	10.9	972	8,909
	Democratic Republic of Congo, Pew study^†^ [28]	2010				9	70*	773
	Eritrea, EPHS [36]	2010	83.0	8,495	10,238	33.2	2,948*	8,879
	Ethiopia, DHS [35]	2016	65.2	5,101	7,822	15.7	1,147	7,306
	Ethiopia, Pew study^†^ [28]	2010				33	204*	618
	Gambia, DHS [37]	2019–2020	72.6	4,490	6,186	45.9	2,343	5,105
	Ghana, MICS [38]	2017–2018	2.4	341	14,374	0.1	15	12,015
	Ghana, Pew study^†^ [28]	2010				9	63*	699
	Guinea, DHS [39]	2018	94.5	10,276	10,874	39.1	3,563	9,122
	Guinea Bissau, MICS [40]	2018–2019	52.1	5,703	10,945	29.7	2,558	8,625
	Guinea-Bissau, Pew study^†^ [28]	2010				33	178*	539
	Kenya, DHS [41]	2014	21	3,066	14,625	2.8	352	12,388
	Kenya, Pew study^†^ [28]	2010				10	76*	762
	Liberia, DHS [42] ^#^	2019–2020	38.2	2,568	6,716			
	Liberia, Pew study^†^ [28]	2010				21	182*	866
	Mali, DHS [43]	2018	88.6	4,699	5,302	72.7	4,314	5,939
	Mali, Pew study^†^ [28]	2010				77	447*	581
	Mauritania, MICS [44]	2015	66.6	9,555	14,342	53.2	6,936	13,048
	Mozambique, Pew study^†^ [28]	2010				12	76*	631
	Niger, DHS [46]	2012	2	219	11,160			
	Nigeria, DHS [45]	2018	19.5	5,202	26,705	19.2	4,640	24,143
	Nigeria, Pew study^†^ [28]	2010				13	106*	813
	Rwanda, Pew study^†^ [28]	2010				3	15*	499
	Senegal, DHS [47]	2019	25.2	2,181	8,649	16.1	1,176	7,288
	Senegal, Pew study^†^ [28]	2010				4	21*	537
	Sierra Leone, DHS [48]	2019	83	12,932	15,574	7.9	946	12,037
	South Africa, Pew study^†^ [28]	2010				4	33*	819
	Tanzania, DHS [49]	2015–2016	10	1,329	13,266	0.4	47	11,795
	Tanzania, Pew study^†^ [28]	2010				6	64*	1,074
	Togo, MICS [50]	2017	3.1	225	7,326	0.3	17	6,077
	Uganda, DHS [51]	2016	0.3	56	18,506			
	Uganda, Pew study^†^ [28]	2010				13	89*	682
	Zambia, ZSBS [52]	2009	0.7	15*	2,206			
	Zambia¸ Pew study^†^ [28]	2010				3	13*	443
**EMR**	Djibouti, Pew study^†^ [28]	2010				58	469*	808
	Djibouti, EVFF [57]	2019	90.1	5,567*	6,179*	31.0	1,225*	3,951*
	Egypt, DHS^¶¶^ [53]	2014	92.3	20,086*	21,762	21.4	4,941*	23,090
	Iraq, MICS [54]	2018	7.4	2,270	30,660	0.5	128	24,438
	Somalia, SHDS [56]	2018–2019	99.2	14,651	14,771	33.3^##^	2,492^##^	7,482^##^
	Sudan, MICS [55]	2014	86.6	15,853	18,302	31.5	5,570	17,661
	Yemen, DHS^†^ [29]	2013	18.5	4,705	25,434	15.9	1,909*	12,005
**SEAR**	Maldives, DHS [58]	2016–2017	12.9	996	7,699	1.1	40*	3,626
	Indonesia, RISKESDAS^††^ [27]	2013				51.2	NA	NA
**Pooled prevalence** ^‡^			**Women 15–49 years**			**Girls 0–14 years** ^¶¶^		
			**Pooled prevalence, % (95% CI)**	**Estimated total number with FGM/C** **(95% CI)**	**Total population** ^Ŧ^	**Pooled prevalence, %** **(95% CI)**	**Estimated total number with FGM/C** **(95% CI)**	**Total population** ^Ŧ^
**Global**			36.90 (19.6–58.3)	84,650,032 (45,009,041–133,834,224)	229,403,880	8.27 (3.7–17.3)	13,734,845 (6,211,405–28,731,901)	166,080,352
**AFR**			28.16 (13.5–49.7)	48,363,907 (23,151,473–85,306,651)	171,746,830	7.83 (3.0–18.7)	10,137,312 (3,935,814–24,223,384)	129,467,580
**EMR**			77.31 (31.7–96.2)	44,486,688 (18,258,474–55,327,837)	57,543,252	14.65 (3.6–44.4)	5,356,258 (1,301,589–16,229,646)	36,561,491

AFR, African Region; DHS, Demographic and Health Survey; EMR, Eastern Mediterranean Region; EPHS, Eritrea Population and Health Survey; EVFF, L’enquête nationale sur les violences faites aux femmes (National survey on violence against women); FGM/C, female genital mutilation/cutting; MICS, Multiple Indicator Cluster Surveys; NA, Not available; SEAR, South East Asian Region; SHDS, Somali Health and Demographic Survey; WHO, World Health Organization.

§The Pew study corresponds to the Islam and Christianity in Sub-Saharan Africa Survey, Pew Research Centre.

§§Year of data collection.

^¶^For girls, studies either reported (1) the percentage/total number of girls with FGM/C or (2) the percentage/total number of women with at least 1 daughter with FGM/C.

^¶¶^In the Egypt DHS 2014 report, the age category of girls is 0 to 19 years.

*The total number with FGM/C was computed using data available in the study/report.

^†^Excluded from the meta-analyses of girls (0–14 years) as results represent the percentage of women with at least 1 daughter with FGM/C.

^††^Excluded from the meta-analyses of girls (0–14 years) due to insufficient data.

^‡^No pooled prevalence was calculated for SEAR as data were only available from 1 country.

^#^Liberia: among women who have heard of FGM/C.

^##^Somalia: computed using the dataset as no denominator was provided in the report.

^Ŧ^Population estimates were taken from the United Nations 2019 Revision of World Population Prospects total population estimates for 2020 [26].

Within AFR, the prevalence among women was 28.2% (95% CI: 13.5% to 49.7%; PI: 0.3% to 97.9%; τ^2^ = 5.1), while among girls, it was 7.8% (95% CI: 3.0% to 18.7%; PI: 0.1% to 91.1%; τ^2^ = 4.9). This provided a regional estimate of 48,363,907 (95% CI: 23,151,473 to 85,306,651) women with FGM/C and 10,137,312 (95% CI: 3,935,814 to 24,223,384) girls with FGM/C. Within EMR, the prevalence among women was 77.3% (95% CI: 31.7% to 96.2%; PI: 0.2% to 100%; τ^2^ = 6.2), while among girls, it was 14.7% (95% CI: 3.6% to 44.4%; PI: 0.04% to 98.7%; τ^2^ = 3.1). This provided an EMR regional estimate of 44,486,688 (95% CI: 18,258,474 to 55,327,837) women with FGM/C and 5,356,258 (95% CI: 1,301,589 to 16,229,646) girls with FGM/C.

Among available nationally representative surveys that ranged between 1994 and 2020, most countries showed a decline in the prevalence of FGM/C across repeated cross-sections of women and girls (26 countries for both women and girls) (Table 2). In addition, among repeated cross-sections of women, 6 countries showed a minor decrease in prevalence (0% to 3%, not including Uganda) and 3 countries showed an increase in the prevalence of FGM/C. In particular, there was an increase from 97.9% to 99.2% in Somalia (2006 to 2020), from 71.6% to 75.8% in Burkina Faso (1998–1999 to 2010), and from 44.5% to 52.1% in Guinea-Bissau (2006 to 2018–2019). For repeated cross-sections of girls, 2 countries had a minor decrease in prevalence (0% to 3%, not including Togo or Niger) and 1 country had an increase (Cameroon: 0.7% in 2004 to 1.0% in 2010). The largest decline was in Central African Republic (43.4% in 1994–1995 to 21.6% in 2018–2019) among repeated cross-sections of women and in Ethiopia from 51.9% in 2000 to 15.7% in 2016, which was among women who reported having at least 1 daughter who had FGM/C in 2000 and among girls in 2016.

**Table 2:** Repeated nationally representative cross-sectional studies reporting the prevalence of FGM/C by country.

Region	Country	Date of survey	% FGM/C among women	Total sample size of women	% FGM/C among girls	Total sample size of girls	Survey source
AFR	Benin	2001*	**17.0**	6,219	**6.7**	3,681	DHS
		2006*	**12.9**	17,794	**2.2**	11,067	
		2011–2012	**7.3**	16,599	**0.3**	10,671	
		2014	**9.2**	15,815	**0.2**	9,902	MICS
	Botswana	2010*			**5.0**	399	Pew Res Center
	Burkina Faso	1998–1999**	**71.6**	6,445	**45.5**	3,499	DHS
		2003*	**76.6**	12,477	**31.6**	7,540	
		2006*	**72.5**	7,316	**24.7**	4,548	MICS
		2010	**75.8**	17,087	**13.3**	17,434	DHS
	Cameroon	2004*	**1.4**	5,391	**0.7**	2,975	DHS
		2010*			**1.0**	755	Pew Res Center
	Central African Republic	1994–95	**43.4**	5,884			DHS
		2000	**35.9**	16,941			
		2006*	**25.7**	11,592	**6.6**	6,778	MICS
		2010	**24.2**	11,510	**0.8**	17,441	
		2018–2019	**21.6**	9,202	**1.4**	9,704	
	Chad	2004*	**44.9**	6,085	**20.7**	3,893	DHS
		2010	**44.2**	15,936	**12.1**	15,936	MICS
		2010*			**39.0**	779	Pew Res Center
		2014–2015	**38.4**	11,534	**9.9**	14,310	DHS
		2019	**34.1**	22,561	**7.0**	26,303	MICS
	Côte D’Ivoire	1994	**42.7**	8,099			DHS
		1998–99**	**44.5**	3,040	**13.5**	3,040	DHS
		2005	**41.7**	5,183			DHS
		2006	**36.0**	12,888	**9.5**	12,888	MICS
		2011–2012	**38.2**	10,060	**10.5**	8,110	DHS
		2016	**36.7**	11,780	**10.9**	8,909	MICS
	Democratic Republic of Congo	2010*			**9.0**	773	Pew Res Center
	Eritrea	1995**	**94.5**	5,054	**71.4**		DHS
		2002*	**88.7**	8,754	**62.5**	4,604	
		2010	**83.0**	10,238	**33.2**	8,879	EPHS
	Ethiopia	2000*	**79.9**	15,367	**51.9**	7,659	
		2005*	**74.3**	14,070	**37.7**	7,920	DHS
		2010*			**33.0**	618	Pew Res Center
		2011			**23.0**		WMS
		2016	**65.2**	7,822	**15.7**	7,306	DHS
	Gambia	2005–2006*	**78.3**	9,982	**64.3**	5,337	MICS
		2010*	**76.3**	14,685	**42.4**	16,635	
		2013	**74.9**	10,233			DHS
		2018	**75.7**	13,640	**50.6**	11,718	MICS
		2019–2020	**72.6**	6,186	**45.9**	5,105	DHS
	Ghana	2006	**3.8**	5,890			MICS
		2010*			**9.0**	699	Pew Res Center
		2011	**3.8**	10,627	**0.4**	8,276	MICS
		2017–2018	**2.4**	14,374	**0.1**	12,015	
	Guinea	1999*	**98.6**	6,753	**54.4**	4,240	
		2005*	**95.6**	7,954	**56.8**	4,972	DHS
		2012	**96.9**	9,142	**45.5**	8,497	
		2016	**96.8**	9,663	**45.3**	8,832	MICS
		2018	**94.5**	10,874	**39.1**	9,122	DHS
	Guinea Bissau	2006*	**44.5**	8,010	**34.7**	4,575	MICS
		2010*	**49.8**	18,734	**38.7**	10,563	
		2010*			**33.0**	539	Pew Res Center
		2014	**44.9**	10,234	**29.6**	8,267	MICS
		2018–2019	**52.1**	10,945	**29.7**	8,625	
	Kenya	1998**	**37.6**	7,881	**24.1**	1,590	DHS
		2003**	**32.2**	8,195	**21.0**	1,577	
		2008–2009	**27.1**	8,444			
		2010*			**10.0**	762	Pew Res Center
		2014	**21.0**	14,625	**2.8**	12,388	DHS
	Liberia	2007	**58** ^##^				DHS
		2010*			**21.0**	866	Pew Res Center
		2013	**44.4** ^##^				DHS
		2019–2020^†^	**38.2**	6,716			
	Mali	1995–1996**	**93.7**	9,704	**73.6**	6,399	DHS
		2001*	**91.6**	12,849	**73.0**	8,223	
		2006*	**85.2**	14,583	**68.7**	9,105	
		2009–2010*	**88.5**	26,751	**74.6**		MICS
		2010*			**77.0**	581	Pew Res Center
		2012–2013	**91.4**	10,424	**69.2**	11,857	DHS
		2015	**82.7**		**76.4**		MICS
		2018	**88.6**	5,302	**72.7**	5,939	DHS
	Mauritania	2000–2001*	**71.3**	7,728	**66.2**	3,887	DHS
		2007*	**72.2**	12,549	**65.8**	6,454	MICS
		2011	**69.4**	12,754	**54.8**	10,992	
		2015	**66.6**	14,342	**53.2**	13,048	
	Mozambique	2010*			**12.0**	631	Pew Res Center
	Nigeria	1999**	**25.1**	8,206	**20.2**	4,503	DHS
		2003*	**19.0**	7,620	**9.9**	4,129	
		2007*	**26.0**	24,565	**13.3**	13,124	MICS
		2008*	**29.6**	33,385	**29.9**	11,563	DHS
		2010*			**13.0**	813	Pew Res Center
		2011	**27.0**	30,772	**19.2**	16,874	MICS
		2013	**24.8**	38,948	**16.9**	36,308	DHS
		2016–2017	**18.4**	34,376	**25.3**	17,529	MICS
		2018	**19.5**	26,705	**19.2**	24,143	DHS
	Niger	1998*	**4.5**	7,577	**2.5**	7,577	DHS
		2006*	**2.2**	9,223	**0.9**	6,173	DHS
		2012	**2.0**	11,160			
	Rwanda	2010*			**3.0**	499	Pew Res Center
	Senegal	2005*	**28.2**	14,602	**19.5**	7,419	DHS
		2010*			**4.0**	537	Pew Res Center
		2010–2011	**25.7**	15,688	**12.9**	8,983	DHS
		2012–2013			**17.5**	7,172	
		2014	**24.7**	8,488	**12.9**	7,186	
		2015	**24.2**	8,851	**14.6**	7,529	
		2016	**22.7**	8,865	**13.6**	7,390	
		2017	**24.0**	16,787	**14.0**	14,008	
		2018	**23.3**	9,414	**14.1**	7,598	
		2019	**25.2**	8,649	**16.1**	7,288	
	Sierra Leone	2005	**94.0^##^**		**34** ^##^		MICS
		2008*	**91.3**	7,374	**32.5**	4,590	DHS
		2010	**88.3**	13,359	**10.2**	14,703	MICS
		2013	**89.6**	16,658			DHS
		2017	**86.1**	17,873	**8.4**	12,972	MICS
		2019	**83.0**	15,574	**7.9**	12,037	DHS
	South Africa	2010*			**4.0**	819	Pew Res Center
	United Republic of Tanzania	1996**	**17.9**	8,120	**6.7**	4,753	DHS
		2003–2004	**17.7**	6,863			
		2004–2005*	**14.6**	10,329	**4.2**	6,095	
		2010*	**14.6**	10,139	**3.4**	6,075	
		2010*			**6.0**	1,074	Pew Res Center
		2015–2016	**10.0**	13,266	**0.4**	11,795	DHS
	Togo	2006*	**5.8**	6,211	**1.0**	3,431	MICS
		2010	**3.9**	6,379	**0.4**	4,679	
		2013–2014	**4.7**	9,480	**0.3**		DHS
		2017	**3.1**	7,326	**0.3**	6,077	MICS
	Uganda	2006	**0.6**	8,531			DHS
		2010*			**13.0**	682	Pew Res Center
		2011	**1.4**	8,674			DHS
		2016	**0.3**	18,506			
	Zambia	1998	**4.5**				
		2000	**3.8**	1,791			ZSBS
		2003	**0.6**	2,324			
		2005	**0.9**	2,146			
		2009	**0.7**	2,206			
		2010*			**3.0**	443	Pew Res Center
		EMR	Djibouti	2004	**98.1**	2,741			PAPFAM
				2006*	**93.1**	6,020	**48.5**	1,923	MICS
2010*						**58.0**	808	Pew Res Center
				2019	**90.1**	6,179	**31.0**	3,951	EVFF
Egypt	1995*		**97.0**	14,779	**49.7**	10,847	DHS
	2000*		**97.3**		**49.5**	11,540	
	2003*		**97.0**	9,159	**47.3**	6,587	
	2005		**95.8**	19,474	**27.7**	20,628	
	2008		**91.1**	5,540	**24.1**	16,475	
	2014		**92.3**	21,762	**21.4**	23,090	
	2015		**87.2**	7,906	**14.1**	5,280	
Iraq	2011		**8.1**	55,194	**20.6**	8,759	MICS
	2018		**7.4**	30,660	**0.5**	24,438	
Somalia	2006*		**97.9**	6,764	**46.0**	3,716	MICS
	2018–2019		**99.2**	14,771	**33.3** ^#^	7,482^#^	SHDS
Sudan	1989–1990		**89.2**	5,860			DHS
	2006^**¶¶**^		**69.4**				SHHS
	2010		**88.2** ^**¶**^	16,716	**37.0**	19,084	MICS
	2014		**86.6**	18,302	**31.5**	17,661	
Yemen	1997*		**22.6**	10,414	**19.7**	7,854	DHS
	2013*		**18.5**	25,434	**15.9**	12,005	
SEAR	Indonesia	2013			**51.2**		RISKESDAS
	Maldives	2016–2017	**12.9**	7,699	**1.1**	3,626	DHS

AFR, African Region; DHS, Demographic and Health Survey; EMR, Eastern Mediterranean Region; Empty cell, Not available in report; EPHS, Eritrea Population and Health Survey; EVFF, L’enquête nationale sur les violences faites aux femmes (National survey on violence against women); FGM/C, female genital mutilation/cutting; MICS, Multiple Indicator Cluster Surveys; SEAR, South East Asian Region; SHHS, Sudan Household Health Survey; SHDS, Somali Health and Demographic Survey WMS, Welfare Monitoring Survey; ZSBS, Zambia Sexual Behaviour Survey.

*Women with at least 1 living daughter with FGM/C.

**Women reporting whether their eldest daughter had FGM/C.

^¶^Age range for women 18–49 years old.

^¶¶^Only in North Sudan (Not measured in South Sudan).

†Among women who have heard of FGM/C.

^#^This was computed using the dataset as no denominator was provided in the report.

^##^Asked if they were women part of “Sande” (Liberia) or “Secret society” (Sierra Leone) as a proxy for having FGM/C. Liberia 2013 was calculated manually using the same method as Liberia 2007. For most countries, girls was defined as 0–14 years old; Indonesia (2013) 0–11 years old; Senegal (2010–2011) 0–9 years; Egypt (2015) age 1–14 years old; Egypt (2014) 0–19 years old; Egypt (2008 and 2005) 0–17 years old. Where no survey is indicated, refer to the previous survey.

**Fig 4:**
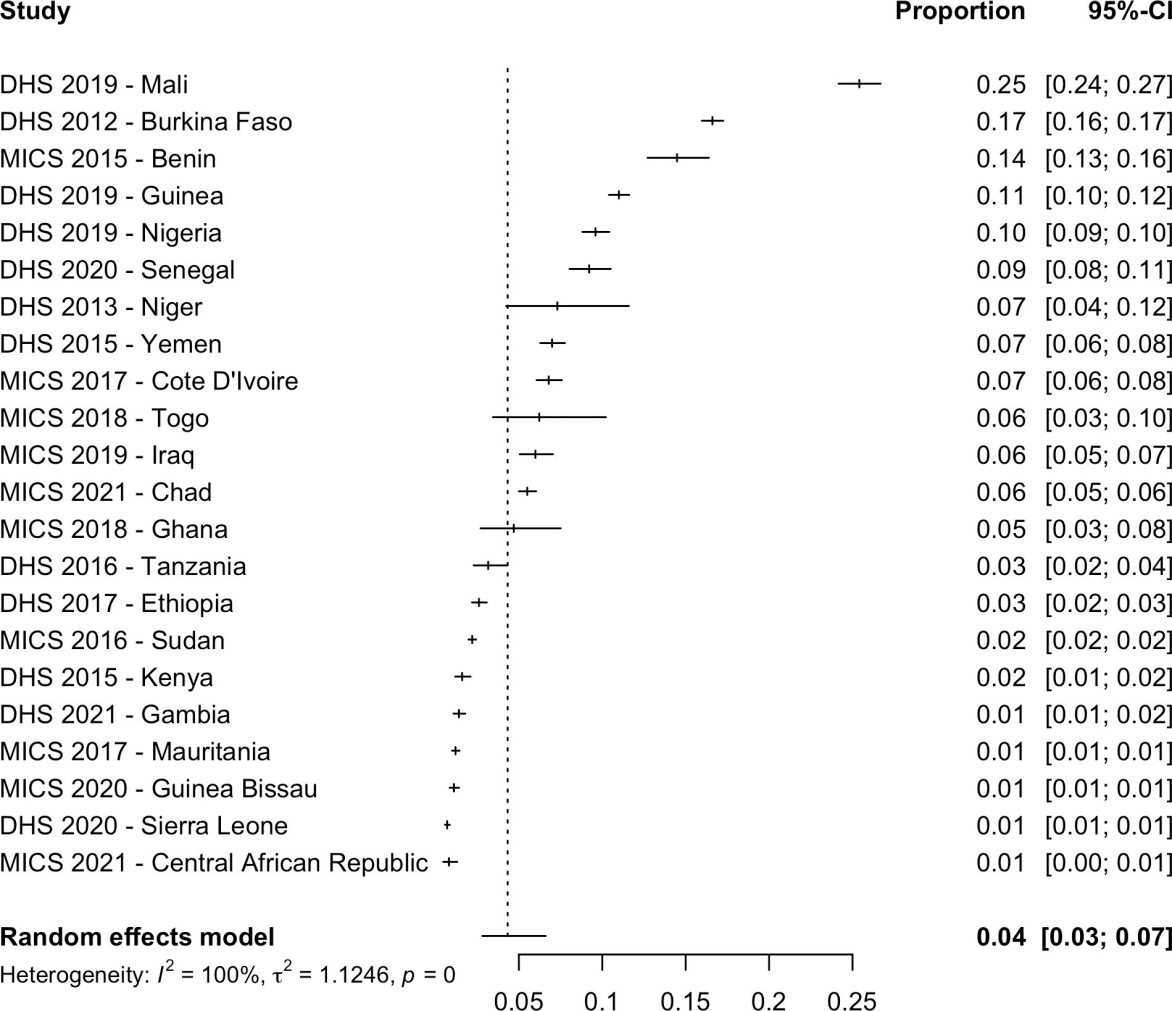
Pooled proportion of women with FGM/C that were “nicked”. The year indicates the date of publication. CI, confidence interval; DHS, Demographic and Health Surveys; MICS, Multiple Indicator Cluster Surveys.

**Fig 5:**
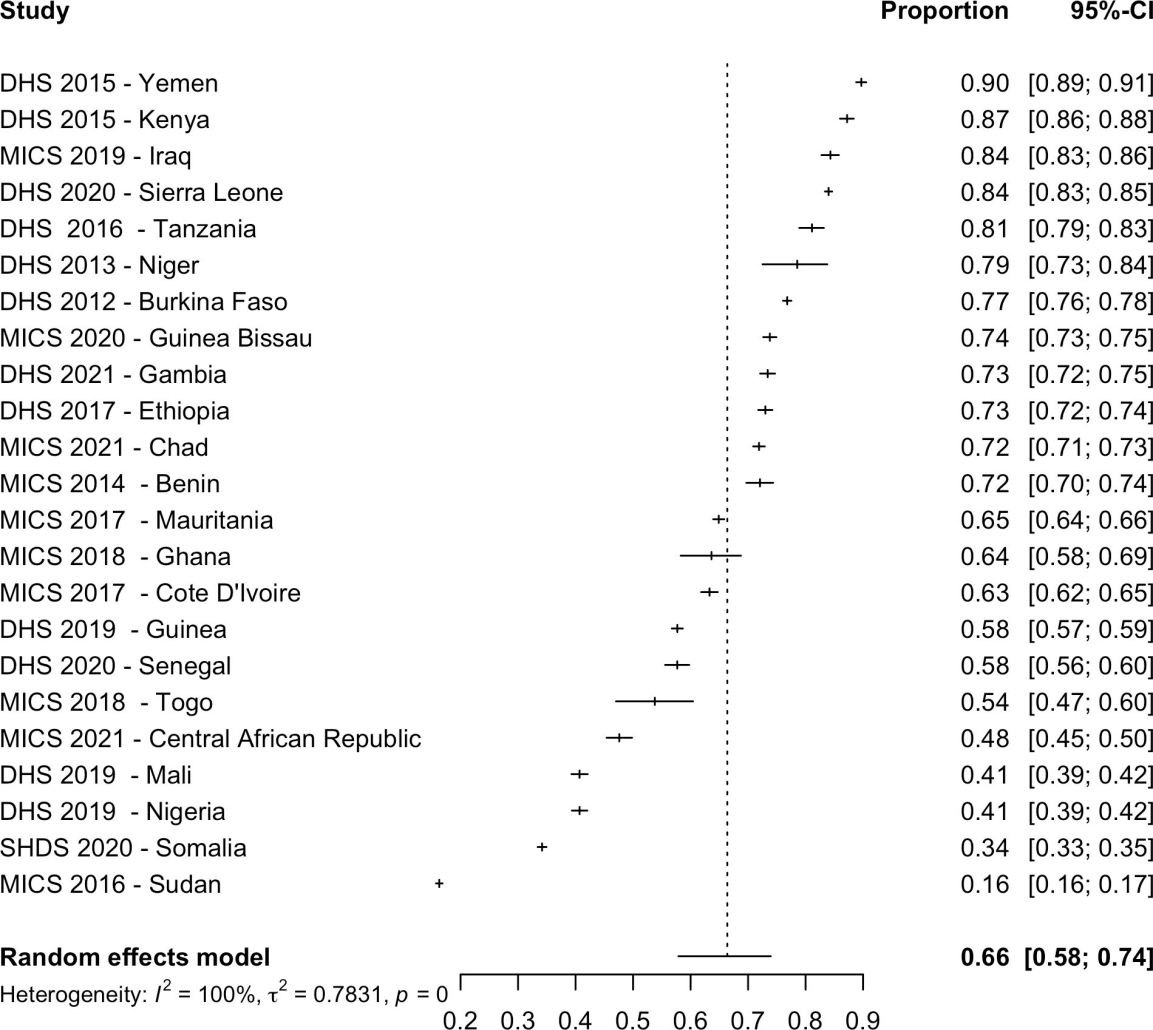
Pooled proportion of women with FGM/C that had “flesh removed”. The year indicates the date of publication. CI, confidence interval; DHS, Demographic and Health Surveys; MICS, Multiple Indicator Cluster Surveys; SHDS, Somali Health and Demographic Survey.

**Fig 6:**
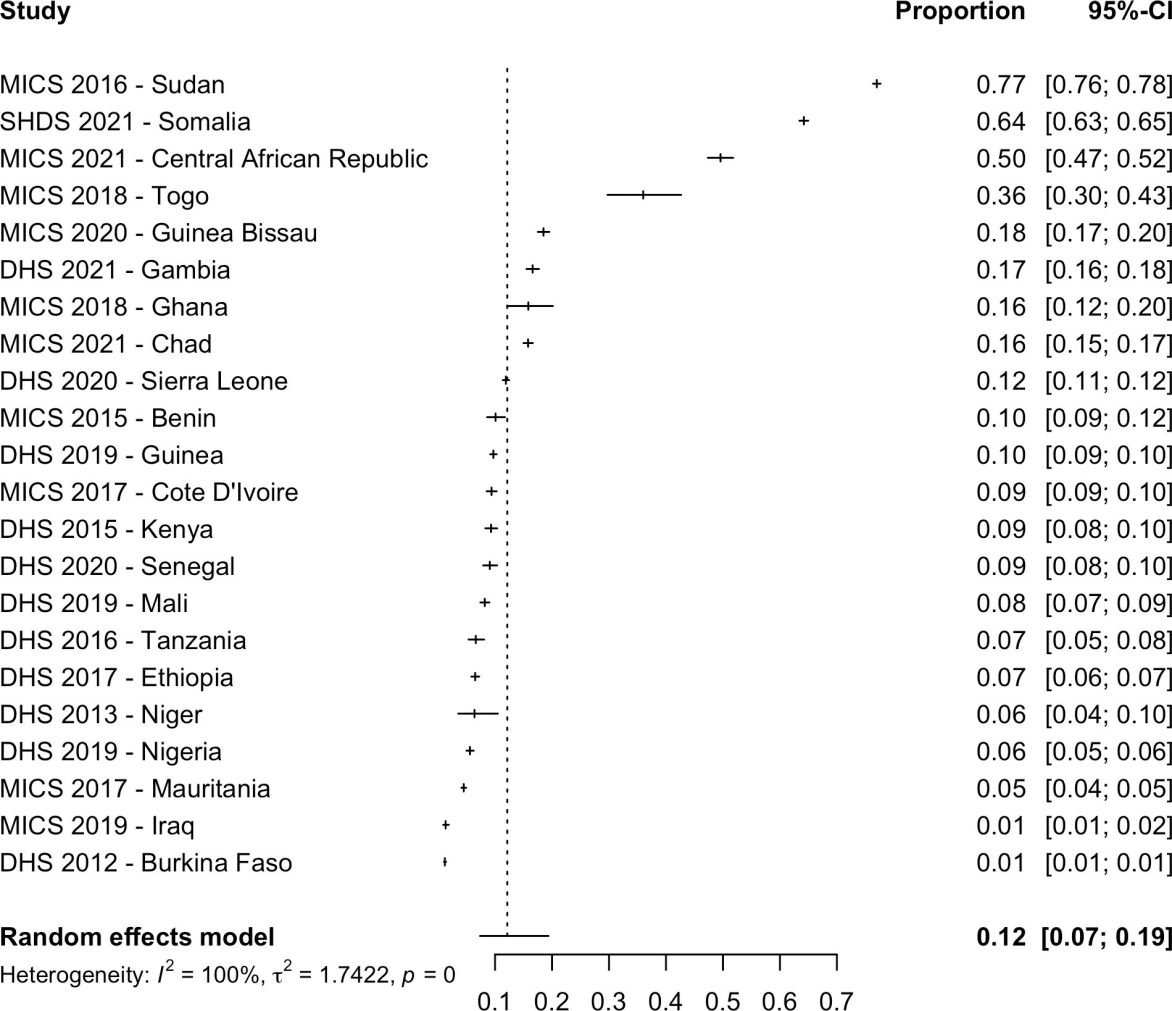
Pooled proportion of women with FGM/C that had their genital area “sewn closed”. Somalia includes both Types III and IV. The year indicates the date of publication. CI, confidence interval; DHS, Demographic and Health Surveys; MICS, Multiple Indicator Cluster Surveys; SHDS, Somali Health and Demographic Survey.

Of the 30 national reports, 23 recorded FGM/C type for women aged 15 to 49 (Table 3). In MICS and DHS, Types I and II were described as “cut with flesh removed”, Type III was described as “sewn closed”, and Type IV was described as “nicked” or “cut”. Among women, the type “flesh removed” was the most common type in 19 countries, “nicked” was the least common type in 14 countries, “sewn closed” was most common among women in 2 countries (Sudan (77.0%) and Central African Republic (49.6%)), and the most common type in Somalia (64.2%) was Types III and IV together (“Pharaonic”). The pooled proportion of women with FGM/C that were “nicked” was 4.3% (95% CI: 2.8% to 6.6%) (Fig 4), had “flesh removed” was 66.4% (95% CI: 57.9% to 73.9%) (Fig 5), and had their genital area “sewn closed” was 12.1% (95% CI: 7.4% to 19.4%) (Fig 6). The age group for Djibouti was not comparable and was not included in the meta-analysis of FGM/C type. No pooled proportion of types was conducted among girls due to inconsistent reporting of types, and it was only collected in 17 out of 25 countries. Among girls with FGM/C, “not sewn closed” and “flesh removed” were the most common type in 8 countries each, and “sewn closed” was the least common type in 11 countries, although it was the most common type in the Central African Republic (59.2%). Surveys using the terms “not sewn closed” may refer to Types, I, II, and IV (Table 3).

**Table 3:** Types of FGM/C in nationally representative studies.

			Women 15–49 years					Girls 0–14 years					
WHO Region	Country, Survey	Year^§^	Prevalence FGM/C, n (%)	Type of FGM/C, %^§§^				Prevalence FGM/C, n (%)	Type of FGM/C, %^§§^				
				Nicked	Flesh removed	Sewn closed	Don’t Know/ Missing Type		Nicked	Not Sewn Closed	Flesh removed	Sewn closed	Don’t Know/ Missing Type
**AFR**	Benin, MICS [30]	2014	1,457 (9.2)	14.5	72.1	10.1	3.4						
	Burkina Faso, DHS [31]	2010	12,949 (75.8)	16.6	76.8	1.2	5.4						
	Central African Republic, MICS [32]	2018–2019	1,983 (21.6)	0.8	47.6	49.6	2	139 (1.4)	0.2		38.6	59.2	2.0
	Chad, MICS [33]	2019	7,698 (34.1)	5.5	71.9	15.8	6.8	1,838 (7.0)	4.4		73.6	18.5	3.5
	Cote D’Ivoire, MICS [34]	2016	4,329 (36.7)	6.8	63.3	9.4	20.5	972 (10.9)	6.3		78.4	10.4	4.9
	Ethiopia, DHS [35]	2016	5,101 (65.2)	2.6	73	6.5	17.9	1,147 (15.7)		90.1		9.3	0.6
	Gambia, DHS [37]	2019–2020	4,490 (72.6)	1.4	73.4	16.6	8.7	2,343 (45.9)		67.8		15.2	17.1
	Ghana, MICS [38]	2017–2018	341 (2.4)	4.6	63.6	15.9	15.9	15 (0.1)			74.5	20.2	5.2
	Guinea, DHS [39]	2018	10,276 (94.5)	11	57.7	9.7	21.6	3,563 (39.1)		84.2		15.8	
	Guinea Bissau, MICS [40]	2018–2019	5,703 (44.5)	1.1	73.8	18.5	6.5	2,558 (29.7)	1.5		81.4	13.4	3.6
	Kenya, DHS [41]	2014	3,066 (21)	1.6	87.2	9.3	1.9	352 (2.8)		86.3		7.8	5.9
	Mali, DHS [43]	2018	4,699 (88.6)	25.4	40.7	8.2	25.8	4,314 (72.7)		88.6		11.4	
	Mauritania, MICS [44]	2015	9,555 (66.6)	1.2	64.9	4.5	29.4	6,936 (53.2)	1.0		75.2	4.2	19.6
	Niger, DHS [46]	2012	219 (2.0)	7.2	78.4	6.3	8.1						
	Nigeria, DHS [45]**	2018	5,202 (19.5)	9.6	40.7	5.6	44.1	4,640 (19.2)		96.5		3.5	
	Senegal, DHS [47]	2019	2,181(25.2)	9.2	57.7	9.1	24.0	1,176 (16.1)		84.3		5.1	10.6
	Sierra Leone, DHS [48]	2020	12,932 (83.0)	0.7	84	11.9	3.3	946 (7.9)		83.3		15.9	0.8
	Tanzania, DHS [49]	2015–2016	1,329 (10.0)	3.2	81.1	6.6	9.1						
	Togo, MICS [50]	2017	225 (3.1)	6.3	54	36.2	3.5						
**EMR**	Djibouti, EVFF [57]	2019						1225 (31.0)*			96.9^#^*	3.1^#^^#^*	
	Iraq, MICS [54]	2018	2,270 (7.4)	6	84.3	1.3	8.4	128 (0.5)	9.1		88.8	1.0	1.1
	Somalia, SHDS [56]	2018–2019	14,651 (99.2)		33.9^¶^	64.2^¶¶^	1.9						
	Sudan, MICS [55]	2014	15,853 (86.6)	2.2	16.3	77	4.5						
	Yemen, DHS [29]	2013	4,705(18.5)	7	89.7	NA	3.3	1,909 (15.9) ^†^	10.7^††^		88.3^††^		1.0^††^

AFR, African Region; DHS, Demographic and Health Survey; EMR, Eastern Mediterranean Region; MICS, Multiple Indicator Cluster Surveys; SHDS, Somali Health and Demographic Survey; WHO, World Health Organization.

^§^Year of data collection.

^§§^Percentages of types from women and girls are calculated from the total number of women and girls with FGM/C, respectively.

^#^Includes Souna (Type I) and Excision (Type II).

^##^Includes Infibulation (Type III).

^¶^ Type I, Sunni = 21.6% and Type II, Intermediate = 12.3%.

^¶¶^ Pharaonic (Type III and IV) = 64.2%.

^†^Prevalence and total number with FGM/C correspond to those of women with at least 1 daughter with FGM/C.

^††^Percent distribution of most recent daughters who had FGM/C.

*The total number and percentages were computed using data available in the study/report.

**All women who had undergone FGM/C in Nigeria were asked about types that are unclassified: angurya (40.4%), gishiri (13.0%), corrosive substances (6.6%).

In all countries, for the majority of women and girls, FGM/C was performed by traditional circumcisers, while a lower proportion was performed by medical professionals. The exception was girls in Egypt, where the proportion of FGM/C performed by medical professionals was 81.9% for girls (Table 4) [53]. For women, in all countries where age of FGM/C was reported, FGM/C was most commonly performed at early ages (0 to 5 years) except for Kenya, Egypt, Sierra Leone, Liberia, and Tanzania where the procedure was most commonly performed at 9 to 14 years or older, and Somalia and Guinea where it was most commonly performed at 5 to 9 years. For girls, age at FGM/C was reported as either a proportion among all girls (with or without FGM/C) at each age group (11 countries) or as a proportion among girls with FGM/C (4 countries).

**Table 4:** Characteristics of FGM/C procedure in nationally representative studies.

			Percent distribution of women 15–49 years by:		Percent distribution of girls 0–14 years by:	
WHO Region	Country, Survey	Year^§^	Age at FGM/C (%)	Performer of FGM/C (%)	Age at FGM/C (%)	Performer of FGM/C (%)
AFR	Burkina Faso, DHS [31]	2010	<5 y (60.4%), 5–9 y (28.2%), 10–14 y (8.9%), 15+ y (2.0%), Don’t know/missing (0.5%)	Traditional (97.2%) Medical (0.2%) Don’t know/missing (2.6%)	<1 y (2.8%), 1–4 y (7.3%), 5–9 y (3.1%), 10–14 y (0.1%), Don’t know/missing (0.1%)*	Traditional (98.3%) Medical (0.2%) Don’t know/missing (1.5%)
	Eritrea, EPHS [36]	2010	<1 y (47.4%), 1–2 y (4.7%), 3–4 (6.5%), 5+ (14.6%), Don’t know/missing (26.9%)	Traditional (84.4%) Medical (0.3%) Other (15.4%)	<1 y (65.7%), 1–4 y (20.9%), 5–6 y (8.1%), 7–8 y (4.3%), 9–10 y (0.4%), 11–12 y (0.1%) 13+ y (0.1%), Don’t know/missing (0.4%)	Traditional (98.3%) Medical (0.09%) Don’t know/missing (1.6%)
	Ethiopia, DHS [35]	2016	<5 y (48.6%), 5–9 y (21.7%), 10–14 y (18.0%), 15+ y (5.9%), Don’t know/missing (5.8%)	Traditional (90.1%) Medical (1%) Don’t know/missing (8.9%)	<1 y (7.2%), 1–4 y (3.4%), 5–9 y (3.7%), 10–14 y (1.0%), Don’t know/missing (0.3%)*	Traditional (97.6%) Medical (1.9%) Don’t know/missing (0.5%)
	Kenya, DHS [41]	2014	<5 y (2.3%), 5–9 y (26.6%), 10–14 y (42.6%), 15+ y (26.9%), Don’t know /missing (1.7%)	Traditional (83.3%) Medical (14.8%) Don’t know/missing (1.9%)	<1 y (0.0%), 1–4 y (0.2%) 5–9 y (2.1%), 10–14 y (0.5%)*	Traditional (74.9%), Medical (19.7%) Don’t know/missing (5.4%).
	Mali, DHS [43]	2018	<5 y (75.5%), 5–9 y (16.1%), 10–14 y (4.4%). 15+ y (0.3%), Don’t know missing (3.6%)	Traditional (91.5%) Medical (0.3%) Don’t know/missing (8.2%)	<1 y (34.2%), 1–4 y (31.9%), 5–9 (5.2%), 10–14 y (0.4%), Don’t know/missing (0.9%)*	Traditional (98.6%) Medical (1.4%)
	Nigeria, DHS [45]	2018	<5 y (85.6%), 5–9 y (4.2%), 10–14 y (3.9%), 15+ y (4.5%), Don’t know/missing (1.8%)	Traditional (85.4%) Medical (8.6%) Don’t know/missing (6%)	<1 y (17.2%), 1–4 y (1.1%), 5–9 (0.7%), 10–14 y (0.0%), Don’t know/missing (0.1%)*	Traditional (92.8%) Medical (7%) Don’t know/missing (0.1%)
	Niger, DHS [46]	2012	<5 y (75.7%), 5–9 y (7.3%), 10–14 y (7.9%), 15+ y (1.4%), Don’t know/missing (7.8%)	Traditional (95.8%) Other (0.2%) Don’t know/missing (4%)		
	Senegal, DHS [47]	2019	<5 y (84.9%), 5–9 y (10.4%), 10–14y (2.7%), 15+ y (0.4%), Don’t know/missing (1.7%)	Traditional (100%)	<1 y (9.8%), 1–4 y (5.4%), 5–9 y (0.8%), 10–14 y (0.0%), Don’t know/missing (0.1)*	Traditional (100%)
	Sierra Leone, DHS [48]	2019	<5 y (12.3%), 5–9 y (14.1%), 10–14 y (44.9%), 15+ y (26.1%), Don’t know/missing (2.5%)	Traditional (98.4%) Medical (0.4%) Don’t know/missing (1.2%)	<1 y (0.0%), 1–4 y (0.6%), 5–9 y (4.1%), 10–14 y (3.1%), Don’t know/missing (0.1%)*	Traditional (99.4%) Medical (0.6%)
	Guinea, DHS [39]	2018	<5 y (22.4%), 5–9 y (36.7%), 10–14 y (28.4%), 15+ y (3.9%), Don’t know/missing (8.6%)	Traditional (77.6%) Medical (17.3%) Don’t know/missing (5.1%)	<1 y (1.5%), 1–4 y (11.9%), 5–9 y (22.7%), 10–14 y (2.3%), Don’t know/missing (0.8%)*	Traditional (64.8%) Medical (34.9%) Don’t know/missing (0.3%)
	Liberia, DHS [42]	2019–2020	<5 y (24.6%), 5–9 y (16.7%), 10–14 y (33%), 15+ y (21.6%), Don’t know (4.1%)			
	Gambia, DHS [37]	2019–2020	<5 y (64.9%), 5–9 y (17.7%), 10–14 y (6%), 15+ y (0.7%), Don’t know/missing (10.6%)	Traditional (95.1%) Medical (0.4%) Don’t know/missing (4.5%)	<1 y (21.9%), 1–4 y (19.4%), 5–9 y (3.9%), 10–14 y (0.2%), Don’t know (0.5%)*	Traditional (98.8%) Medical (0.1%), Don’t know/missing (1.1%)
	Tanzania, DHS [49]	2015–2016	<1 y (35.4%), 1–4 y (2.3%), 5–6 y (5.2%), 7–8 y (7.5%), 9–10 y (10.9%), 11–12 y (9.3%), 13+ y (27.6%), Don’t know/missing (1.8%)	Traditional (86%)	<1 y (0.1%), 1–4 y (0.2%), 5–9 y (0.1%), 10–14 y (0.1%)*	
EMR^†^	Egypt, DHS [53]	2014	<3 y (0.6%), 3–4 y (1%), 5–6 y (7.4%), 7–8 y (13.4%), 9–10 y (40.9%) 11–12 y (24.6%), 13–14 y (5.3%), 15–17 y (2.6%), 18–19 y (0.1%), Don’t know/missing (4.2%)	Traditional (60.5%) Medical (37.9%) Other (0.1%), Don’t know/missing (1.5%)	<3 y (3.5%); 3–4 y (3.4%), 5–6 y (10.1%), 7–8 y (14.1%), 9–10 y (32.8%) 11–12 y (28.6%), 13–14 y (5.4%), 15–17 y (1.3%), Don’t know/missing (0.7%)	Medical (81.9%) Traditional (17.7%) Don’t know/missing (0.3%)
	Somalia, SHDS [56]	2018–2019	<5 y (0.2%), 5–9 y (70.9%), 10–14 y (27.7%), 15+ y (0.7%), Don’t know/missing (0.5%)			
	Yemen, DHS [29]	2013	First week after birth (83.8%), after first week but before first year (10.5%), > = 1 y (1.2%), Don’t know/missing (4.5%)	Traditional (92.8%) Medical (2.9%) Don’t know/missing (4.3%)	First week after birth (84.9%), after first week but before 1 year (14.3%), > = 1 y (0.6%), Don’t know/missing (0.2%)^¶^	Traditional (84.7%) Medical (12.8%) Don’t know/missing (2.5%)^¶^
SEAR	Indonesia, RISKESDAS [27]	2013			1–5 months (72.4%), 1–4 y (13.9%), 5–11 y (3.3%)	
	Maldives, DHS [58]	2016–2017	<5 y (83.1%), 5–9 y (1.6%), 10–14 y (0.4%), 15+ y (0.4%), Don’t know/missing (14.5%)		<1 y (0.7%), 1–4 y (0.2%), Don’t know/missing (0.1%)*	

AFR, African Region; DHS, Demographic and Health Survey; EMR, Eastern Mediterranean Region; MICS, Multiple Indicator Cluster Surveys; SHDS, Somali Health and Demographic Survey; WHO, World Health Organization; y, years.

^§^Year of data collection.

*Row percentages representing the proportion of age group who had FGM/C out of the entire cohort of girls (with and without FGM/C).

^†^In Djibouti (EVFF, 2019), among girls and women of all ages, performers of FGM/C were 93.2% traditional and 6.8% medically trained, and the average age at FGM/C was 5.8 years.

^¶^Most recent daughter with FGM/C.

### Subregional studies

Thirty-two subregional studies were from 13 countries, with 10 studies from EMR and 22 studies from AFR (Table A in S1 Appendix). Among studies including women, the highest FGM/C prevalence was in the Hababo Guduru District, Ethiopia (98.2%) [59], and the lowest was in Axum Town, North Ethiopia (0.7%) [60]. Regarding the 16 subregional studies reporting prevalence among girls, the highest FGM/C prevalence was in Kersa, Ethiopia (88.1%) [61], and the lowest was in Axum Town, Ethiopia (0%) [60] (Table B in S1 Appendix). Ten out of the 32 subregional studies reported on FGM/C type. Types I and II, reported together, were the most common in 3 studies [62–64], Type II was the most common in 3 studies [65–67], and “sewn closed” was the most common in 3 studies in Somalia [68–70] (Table C in S1 Appendix). In 13 studies, the most common performers of FGM/C were traditional circumcisers [59,61–66,69,71–75]. In 3 studies, in Egypt [76,77] and Saudi Arabia [78], medical professionals were more common than traditional performers (Table D in S1 Appendix).

### School, community, or facility-based studies excluding studies on migrant populations

Within 99 school, community, or facility-based studies, 55 studies (excluding studies on migrant populations) were from 17 countries, with 30 studies from countries in AFR, 3 studies from Malaysia in SEAR, and 22 studies from countries in EMR (Table A in S2 Appendix). Thirty-one were hospital/clinic based, 13 school-based, 8 community-based studies, 2 studies were online surveys, and 1 study was both clinic and school based. Fifty studies had a cross-sectional design, and 5 studies were either prospective or retrospective cohort studies. School and university-based studies reported a prevalence ranging from 9.4% in a Nigerian school [79] to 83.3% in Sudanese schools [80]; hospital or clinic-based studies reported a prevalence from 13% in Northern Nigeria [81] to 100% in Sierra Leone [82], and in community-based studies, FGM/C ranged from 0.4% in a Nigerian community [83] to 99.3% in a snowball sample in Malaysia [84] (Table B in S2 Appendix). Twenty-four studies reported on FGM/C types. In 9 studies, Type I was most common [81,85–92], Type II was most common in 5 studies [93–97], Type III in 3 studies [98–100], and Type IV in 3 studies [84,101,102] (Table C in S2 Appendix). Among these studies, age at FGM/C and who performed the FGM/C is reported in Table D in S2 Appendix.

### Studies on migrant populations

Within the 99 school, community, or facility studies, 44 studies on migrant populations with FGM/C were identified. The included studies were from the Region of the Americas (AMR) (9 studies), European Region (EUR) (25 studies), Western Pacific Region (WPR) (5 studies), and EMR (5 studies) (Table A in S3 Appendix). Most studies had a moderate risk of bias and 5 had a high risk of bias. Participants in these studies were categorized as migrants, refugees, or asylum seekers. Study designs were randomized controlled trial (RCT) (*n =* 1), retrospective database analysis studies (*n* = 8), cross-sectional studies (*n* = 19), case series (*n* = 15), and case–control (*n* = 1). Among study designs that can estimate prevalence, the prevalence ranged from 0.32% of women attending midwifery clinics in the Netherlands [103] to 47.8% of Kurdish and Somali women in Finland [104] (Table B in S3 Appendix). Type III was the most common type in 11 studies [105–115], followed by Type II in 5 studies [116–120], Type I (6 studies) [121–126], Types I and II (3 studies) [127–129], and Type IV (2 studies) [130,131] (Table C in S3 Appendix). Age at FGM/C and whether the procedure was performed by medical or traditional practitioner is presented in Table D in S3 Appendix.

## Discussion

This systematic review and meta-analysis estimated that nearly 100 million girls and women of reproductive age had FGM/C, which was among countries included in the analysis. Results indicated that the practice remains widespread in countries where it is reported. Across 30 countries, there was a pooled prevalence of 37% among women aged 15 to 49 years old, and across 25 countries, there was a pooled prevalence of 8% among girls aged 0 to 14 years old. Over repeated cross-sectional surveys, the prevalence of FGM/C appears to have decreased in 26 countries for both women and girls. It appears to have increased in 3 countries for women (Guinea-Bissau, Burkina Faso, and Somalia) and 1 country for girls (Cameroon). For women who had FGM, most had the type “flesh removed” (Types I and II); for girls, most had “flesh removed” or “not sewn closed”, which may include Types I, II, and IV. “Sewn closed” (Type III), the most severe type of FMG/C, was practiced in over three-quarters of women in Sudan and over half of girls in the Central African Republic. In most countries, FGM/C commonly occurred in early childhood and was performed by traditional circumcisers. FGM/C appears to continue in those who migrated from countries where FGM/C is prevalent.

The total prevalence of FGM/C specified in this study was consistent with previous estimates of FGM/C among girls and women of reproductive age where estimates of FGM/C range from 100 to 140 million women and girls [2,3]. Our study findings differ to the most recent UNICEF report, which states the global prevalence of FGM/C to be over 200 million living women and girls, although the upper end of the combined confidence interval was close to this estimate [1]. Potential explanations may be that UNICEF extrapolated their prevalence to women of all ages, this study was unable to include Indonesia in the prevalence estimate, and this study excluded estimates from surveys that used a household level prevalence of FGM/C among girls.

The decline of FGM/C across repeated cross-sectional studies in many countries is encouraging and corresponds with previous research, which showed an absolute decline in the prevalence of FGM/C among girls aged 0 to 14 years by 51.8%; from 67.6% in 1990–1996 to 15.8% in 2015–2017 [5]. Results were consistent with previous research regarding large variations in prevalence between countries and regions [4,5,132].

Structural level changes including legislative bans and policy changes are likely to play a role in the possible decline. Globally, there are 84 countries that either have specific legislation that bans FGM/C or other legislation that enables the prosecution of FGM/C [133,134]. In Egypt, the lower prevalence for girls may relate to the legal ban implemented in 2008 [53,133]. However, the efficacy of laws against FGM/C depends on enforcement and the specificities of the law. For example, in Mauritania, laws only protect girls below the age of 18 [134]. In Indonesia, FGM/C was legalized in a medical setting in 2010; however, the repeal of that law in 2014 left no explicit ban or consequences [134,135]. In Somalia, there is no national legislation that enforces the Somalia constitution, which states that “circumcision is prohibited” [133,134]. Furthermore, there is no legislative ban in Mali, and the prevalence remains high at 88.6% of women and 72.7% of girls [43].

In addition to legislation and judicial enforcement, other mechanisms may have contributed to a reduction in FGM/C, such as education, literacy, and change in social norms [136,137]. To end the propagation of FGM/C, future research should undertake process evaluations of structural, community, and family-level interventions and policies in countries where FGM/C has declined. Understanding the underlying mechanisms for change in FGM/C, in countries where there has been success, will be instrumental for the adoption of effective policies and interventions to meet the SDG target 5.3.

Consistent with other studies, the most common FGM/C type among women and girls was “cut with flesh removed”, equivalent to Type I or II [4,138]. Koski and colleagues reported that there were no significant differences regarding the types and severity of FGM/C across cohorts [4]. Similar to other findings, this review found that FGM/C most often occurs in early childhood [138].

Similar to the findings of this study, UNICEF reported that traditional circumcisers perform most procedures [138]. Yet, the opposite occurs in Egypt where medicalization of FGM/C was high despite its ban [53]. WHO and UNICEF have called for the end of medicalization of FGM/C [139,140]. Discussions around the medicalization of FGM/C are beyond the scope of this study, but this has been discussed elsewhere [141,142].

There was variation in reports of FGM/C prevalence between different studies within the same country, a phenomenon also reported by UNICEF [138], likely owing to regional or community risk factors. For example, the national prevalence in Ethiopian women was 65.2% [35], while in 1 region, the East Gojjam Zone, it was 96% [65]. Studies based on migrant populations have widely varying prevalence estimates. They demonstrate that FGM/C is present in countries where it is not traditionally practiced; however, high-quality studies are needed to understand FGM/C in these countries and to inform policies, interventions, and relevant healthcare services.

The strengths of the study include a thorough and accurate examination of the research question. The review had broad inclusion criteria to provide a comprehensive review of all FGM/C studies. The study used robust methods to identify studies, extract data, and present findings. The broadest possible scope of research was scanned with no restrictions on language and a hand search of gray literature was conducted. Moreover, DHS and MICS data, which are collected via probability sampling methods with high response rates and a low risk of bias, ensured the quality of the meta-analyses.

This study had several limitations. Estimates were based on the available published data, which may not reflect the actual global prevalence of FGM/C. The actual total number of girls and women with FGM/C globally will be higher than that reported in this study due to missing data from key countries. In addition, Indonesia was not included in the meta-analysis due to lack of a denominator. FGM/C was self-reported, thus the prevalence estimates may be underreported due to legal ramifications or social desirability. Furthermore, the translation of terms within surveys may affect recall and comprehension, which emphasizes the need for survey tools to be validated within each context. In addition, women and girls may not be able to accurately recall the type of procedure performed on them, or there may be confusion due to multiple ways of describing each type [143].

The prevalence in the 0 to 14 age group may be underreported as these girls are still at risk of FGM/C at the time of survey. Future research should adjust prevalence by age at FGM/C procedure or conduct analyses based on age cohorts to be inclusive of those still at risk of FGM/C. A future study examining FGM/C prevalence among 5-year age cohorts will be useful to understand if trends exist across age groups [138]. This study also shows the need for consistency in the denominator of FGM/C among girls and terminology used to describe each type of FGM/C.

This study highlights the need to expand data collection and surveillance using robust methodologies particularly in high-resource countries with migrant populations from countries that practice FGM/C. There are numerous data gaps on the national prevalence of FGM/C in multiple countries, including Colombia, Georgia, Russia, Iran, Oman, Kuwait, Singapore, Thailand, the Philippines, India, Pakistan, Ecuador, Peru, Saudi Arabia, the State of Palestine, Sri Lanka, and United Arab Emirates [144]. In Indonesia, approximately 50% of girls aged 0 to 14 had FGM/C; however, we know relatively little about FGM/C in Indonesia, which warrants further investigation given its large population size.

In conclusion, approximately 100 million women and girls have had FGM/C among countries included in the analysis, and there is large variation between countries in progress to ending FGM/C by 2030. Current findings may be used as a baseline in future attempts to track progress to meeting SDG 5.3. Ending FGM/C in the next generation of girls may be possible in the near future in low-prevalence countries such as Niger, Uganda, and Ghana. However, the decline in FGM/C must be greater in countries where the current prevalence of FGM/C is high such as Egypt, Sudan, Indonesia, Somalia, Djibouti, Guinea, and Mali, thus emphasizing the need for immediate interventions and policies to end this harmful practice.

## Supporting information

S1 PRISMA Checklist PRISMA 2020 Checklist. (DOCX)

S1 Table Search strategy. (DOCX)

S2 Table Interrater reliability rate at different stages of the screening process. (DOCX)

S3 Table Characteristics of nationally representative studies. *Not included in meta-analysis. **Includes countries in AFR and EMR. ***EVFF: L’enquête nationale sur les violences faites aux femmes (National survey on violence against women).
(DOCX)

S1 Text Inclusion and exclusion criteria. (DOCX)

S2 Text Supplementary results. (DOCX)

S1 Study Protocol The global prevalence, distribution, and determinants of female genital mutilation: A protocol for a systematic review and meta-analysis. (DOCX)

S1 Fig Funnel plot of FGM/C prevalence in women of reproductive age (15–49 years old) in nationally representative studies. (TIF)

S2 Fig Funnel plot of FGM/C prevalence in girls (0–14 years old) in nationally representative studies. (TIF)

S1 Appendix Subregional population-based studies. Table A. Characteristics of subregional population-based studies. All studies used cross-sectional methods. Table B. Prevalence of FGM/C in women and girls in subregional population-based studies. *Women reported that at least 1 daughter had FGM/C in the household. ^†^Youngest daughter had FGM/C. ^‡^Due to inconsistent data reported in the study, this number was calculated by the authors of this review. **Total FGM/C and sample size for women and girls were excluded due to conflicting numbers within the report. Table C. Types of FGM/C in subregional population-based studies. *% of women; ^†^% of youngest daughter; ^¶^At least 1 living daughter; ^‡^% of girls. #Pharaonic (Type III or IV); Northeast Zone MICS calculates the prevalence of type out of the total number of participants. Table D. Characteristics of FGM/C procedure in subregional population-based studies. *The report was unclear about the percentages of the performers of FGM/C in women and daughters.
(DOCX)

S2 Appendix School, community, or facility-based studies excluding studies on migrant populations. Table A. Characteristics of school, community, or facility-based studies excluding studies on migrant populations. Table B. Studies reporting FGM/C in women and girls in school, community, or facility-based studies excluding studies on migrant populations. ^#^Among respondents aware of FGM/C. ^##^Out of 342 women, 49 reported FGM/C in daughter(s) (14.3%).*Out of the female school teachers. **Without excluding those who were unsure if they had been mutilated. ***Prevalence according to clinical examination. Table C. Types of FGM/C in school, community, or facility-based studies excluding studies on migrant populations. *Clitoridectomy; **Manually calculated from the report; ^†^Flesh removed (Type I or II); ^¶^Genital area was sewn after cutting; ^‡^Genital area was nicked (without cutting); ^#^Clitoral nicking. Table D. Characteristics of FGM/C procedure in school, community, or facility-based studies excluding studies on migrant populations.
(DOCX)

S3 Appendix Studies on migrant populations. Table A. Characteristics of studies on migrant populations. *Conflicting numbers within the study. The methods state that this was part of a larger study of 338 women with FGM/C. The results are based on 188 women with FGM/C Type III. ^†^Calculated manually using data available in the report. Table B. Studies reporting FGM/C in migrant populations. *Calculated manually. Table C. Types of FGM/C in migrant populations. *Flesh removed. ^‡^% of mothers. ^¶^% of daughters. **Pharaonic (Type III). ^†^Genital area cut without flesh removed or nicked. ^§^Genital area sewn closed. ^Δ^Tissue removed and some stitching. ^#^Includes 2% untouched in the proportion. ^##^Includes 2% none in the proportion. Table D. Characteristics of FGM/C procedure for migrant populations.
(DOCX)

## Abbreviations:

AFR: African Region
AMR: Region of the Americas
CI: confidence interval
DHS: Demographic and Health Surveys
EMR: Eastern Mediterranean Region
EUR: European Region
FGM/C: female genital mutilation/cutting
GLMM: generalized linear mixed model
MeSH: Medical Subject Headings
MICS: Multiple Indicator Cluster Surveys
PI: prediction interval
RCT: randomized controlled trial
SDG: Sustainable Development Goal
SEAR: South East Asian Region
WHO: World Health Organization
WPR: Western Pacific Region
