# Supplementary material for: The global prevalence of female genital mutilation/cutting: A systematic review and meta-analysis of national, regional, facility, and school-based studies
Source: PLoS Med. 2022 Sep 1;19(9):e1004061. doi: 10.1371/journal.pmed.1004061 (PMC9436112; doi:10.1371/journal.pmed.1004061)
Supplement: S1 Table — (DOCX) [file pmed.1004061.s002.docx]

## S1 Table. Search strategy

| **Database** | **Search Terms** |
| --- | --- |
| PsycINFO | 1 Female Genital Mutilation;2 Female Circumcision;3 Female Genital Cutting; 4 Female Genital Alteration; 5 or/1-4; 6 Limit 5 by 2009-2022 |
| PubMed | (("Female Genital Alteration") OR ("Female Genital Cutting") OR ("Female Circumcision") OR ("Female Genital Mutilation")) 2009:2022 [dp] |
| Embase | ('female genital mutilation'/exp OR 'female genital mutilation' OR (('female' OR 'female'/exp OR female) AND genital AND ('mutilation' OR 'mutilation'/exp OR mutilation))) AND [2009-2022]/py |
| Ovid MEDLINE(R) and In-Process, In-Data-Review & Other Non-Indexed Citations  <1946 to March 23, 2022> | 1 Female Genital Mutilation.mp.  2 Female Circumcision.mp.  3 Female Genital Cutting.mp.  4 Female Genital Alteration.mp.  5 1 or 2 or 3 or 4 (1831)  6 Circumcision, Female/ (1569)  7 5 or 6 (2208)  8 ((female adj3 circumcis*) or (girl adj3 circumcis*) or (wom?n adj3 circumcis*)).mp.  9 ((female adj3 genital* adj3 cut*) or (girl adj3 genital* adj3 cut*) or (wom?n adj3 genital* adj3 cut*)).mp.  10 ((female adj3 genital* adj3 alteration*) or (girl adj3 genital* adj3 alteration*) or (wom?n adj3 genital* adj3 alteration*)).mp.  11 ((female adj3 genital* adj3 mutilation) or (girl adj3 genital* adj3 mutilation) or (wom?n adj3 genital* adj3 mutilation)).mp.  12 ((female adj3 genital* adj3 alter) or (girl adj3 genital* adj3 alter) or (wom?n adj3 genital* adj3 alter)).mp.  13 8 or 9 or 10 or 11 or 12 (2358)  14 5 or 7 or 13 (2358)  15 limit 14 to yr="2009 -Current" (1364) |
| Web of Science | 1 Female Genital Mutilation; 2 Female Circumcision;3 Female Genital Cutting; 4 Female Genital Alteration, 5 or/1-4; 6 Limit 5 by 2009-2022 & Document Type: Articles |
