# Supplementary material for: The global prevalence of female genital mutilation/cutting: A systematic review and meta-analysis of national, regional, facility, and school-based studies
Source: PLoS Med. 2022 Sep 1;19(9):e1004061. doi: 10.1371/journal.pmed.1004061 (PMC9436112; doi:10.1371/journal.pmed.1004061)
Supplement: S2 Table — (DOCX) [file pmed.1004061.s003.docx]

S2 Table. Inter-rate reliability at different stages of the screening process.

|  | Stage of systematic review | Cohen’s Kappa |
| --- | --- | --- |
| Stage 1 | Agreement after initial screening of titles and abstracts  Coding decisions and conflicts were discussed and a third reviewer (experienced systematic reviewer) assisted in decision making | 0.34 |
| Stage 1 | Agreement after completing conflict resolution and consensus | 0.95 |
| Stage 2 | Agreement after initial screening of full text  Coding decisions and conflicts were discussed to reach consensus and a third reviewer (experienced systematic reviewer) assisted in decision making. | 0.59 |
| Stage 2 | Agreement after completing conflict resolution and consensus | 1.0 |

A third reviewer confirmed the inclusion of all studies. The Cohen’s kappa provided a global score across all three inclusion criteria; after the full text screening it was decided that the risk factors of FGM/C would be presented in a separate paper. At stage 1, reviewers had the option to indicate if they were unsure, which may also partially explain the low score before resolution. Agreement was higher on the first two points of the inclusion criteria: (i) prevalence studies and (ii) non-population based studies examining FGM/C.
