# Supplementary material for: The global prevalence of female genital mutilation/cutting: A systematic review and meta-analysis of national, regional, facility, and school-based studies
Source: PLoS Med. 2022 Sep 1;19(9):e1004061. doi: 10.1371/journal.pmed.1004061 (PMC9436112; doi:10.1371/journal.pmed.1004061)
Supplement: S3 Table — *Not included in meta-analysis. **Includes countries in AFR and EMR. ***EVFF: L’enquête nationale sur les violences faites aux femmes (National survey on violence against women). (DOCX) [file pmed.1004061.s004.docx]

**S3 Table.** Characteristics of nationally representative studies

| **WHO Region** | **Country, Survey** | **Author** | **Publication date** | **Year of data collection** | **Population description** | **Age category (years)** | **Type** |
| --- | --- | --- | --- | --- | --- | --- | --- |
| **AFR** | **Benin, MICS[1]** | Institut National de la Statistique et de l’Analyse Économique | 2015 | 2014 | Women of reproductive age and girls. | 0-14; 15-49 | Flesh removed, Nicked, Sewn closed. |
|  | **Burkina Faso, DHS[2]** | National Institute of Statistics and Demography (INSD) and ICF International | 2012 | 2010 | Women of reproductive age and girls. | 0-14; 15-49 | Flesh removed, Nicked, Sewn closed. |
|  | **Central African Republic, MICS [3]** | Institut Centrafricain des Statistiques et des Etudes Economiques et Sociales | 2021 | 2018-2019 | Women of reproductive age and girls. | 0-14; 15-49 | Flesh removed, Nicked, Sewn closed. |
|  | **Chad, MICS [4]** | National Institute for Statistics, Economic and Demographic Studies (INSEED) & UNICEF | 2021 | 2019 | Women of reproductive age and girls. | 0-14; 15-49 | Flesh removed, Nicked, Sewn closed. |
|  | **Cote D'Ivoire, MICS[5]** | Institut National de la Statistiques (INS) | 2017 | 2016 | Women of reproductive age and girls. | 0-14; 15-49 | Flesh removed, Nicked, Sewn closed. |
|  | **Ethiopia, DHS[6]** | Central Statistical Agency & ICF | 2017 | 2016 | Women of reproductive age and girls. | 0-14; 15-49 | Cut with flesh removed, Cut with no flesh removed, Sewn closed, Not sewn closed. |
|  | **Eritrea, Population and Health Survey [7]** | National Statistics Office | 2013 | 2010 | Women of reproductive age and their daughters. | 0-14; 15-49 | NA |
|  | **Gambia, DHS[8]** | Gambia Bureau of Statistics | 2021 | 2019-2020 | Women of reproductive age and girls. | 0-14; 15-49 | Flesh Removed, Nicked, Sewn Closed, Not sewn closed. |
|  | **Ghana, MICS[9]** | Ghana Statistical Service | 2018 | 2017- 2018 | Women of reproductive age and girls. | 0-14; 15-49 | Flesh removed, Nicked, Sewn closed. |
|  | **Guinea, DHS[10]** | Institut National de la Statistique Ministère du Plan et du Développement Economique & ICF | 2019 | 2018 | Women of reproductive age and girls. | 0-14; 15-49 | Flesh Removed, Nicked, Sewn closed, Not sewn closed. |
|  | **Guinea Bissau, MICS[11]** | Ministério da Economia, do Plano e Integração Regional | 2020 | 2018-2019 | Women of reproductive age and girls. | 0-14; 15-49 | Flesh Removed, Nicked, Sewn closed, Not determined. |
|  | **Kenya, DHS[12]** | Kenya National Bureau of Statistics & ICF | 2015 | 2014 | Women of reproductive age and girls. | 0-14; 15-49 | Cut with flesh removed, Cut with no flesh removed, Sewn closed, Not sewn closed. |
|  | **Liberia, DHS[13]** | Liberia Institute of Statistics and Geo-Information Services (LISGIS) | 2021 | 2019-2020 | Women of reproductive age who have heard of FGM/C. | 15-49 | NA |
|  | **Mali, DHS[14]** | Institut National de la Statistique (INSTAT), Cellule de Planification et de Statistique Secteur Santé-Développement Social et Promotion de la Famille (CPS/SS-DS-PF) & ICF. | 2019 | 2018 | Women of reproductive age and girls. | 0-14; 15-49 | Cut with flesh removed, Cut with no flesh removed, Sewn closed, Not sewn closed. |
|  | **Mauritania, MICS[15]** | Mauritania National Statistics Office | 2017 | 2015 | Women of reproductive age and girls. | 0-14; 15-49 | Cut and no flesh removed, Cut and flesh removed, Sewn closed . |
|  | **Nigeria, DHS[16]** | National Population Commission (NPC) [Nigeria] & ICF. | 2019 | 2018 | Women of reproductive age and girls. | 0-14; 15-49 | Cut and no flesh removed, Cut and flesh removed, Sewn closed, Not sewn closed. |
|  | **Niger, DHS[17]** | Institut National de la Statistique (INS) & ICF | 2013 | 2012 | Women of reproductive age. | 15-49 | Flesh removed, Nicked, Sewn closed. |
|  | **Senegal, DHS [18]** | Agence Nationale de la Statistique et de la Démographie (ANSD) | 2020 | 2019 | Women of reproductive age and girls | 0-14; 15-49 | Flesh removed, Nicked, Sewn closed, Not Sewn closed. |
|  | **Sierra Leone DHS[19]** | Statistics Sierra Leone & ICF | 2020 | 2019 | Women of reproductive age and girls. | 0-14; 15-49 | Flesh Removed, Nicked, Sewn closed, Not Sewn closed. |
|  | **Tanzania, DHS[20]** | Ministry of Health, Community Development, Gender, Elderly and Children (MoHCDGEC) [Tanzania  Mainland], Ministry of Health (MoH) [Zanzibar], National Bureau of Statistics (NBS), Office of the Chief Government Statistician (OCGS) & ICF. | 2016 | 2015- 2016 | Women of reproductive age and their daughters. | 0-14; 15-49 | Cut, with flesh removed; Cut with no flesh removed, Sewn closed |
|  | **Togo, MICS[21]** | Institut National de la Statistique et des Etudes Economiques et Démographiques (INSEED) | 2018 | 2017 | Women of reproductive age and girls. | 0-14; 15-49 | Cut with flesh removed, Cut with no flesh removed, Sewn closed. |
|  | **Uganda, DHS[22]** | Uganda Bureau of Statistics (UBOS) & ICF | 2018 | 2016 | Women of reproductive age. | 15-49 | NA |
|  | **Zambia, Sexual Behaviour Survey [23]** | Central Statistical Office (CSO) Ministry of Health (MOH) University of Zambia and MEASURE Evaluation | 2010 | 2009 | Women of reproductive age. | 15-49 | NA |
|  | **Multi-country, Tolerance and Tension: Islam and Christianity in Sub-Saharan Africa** [24]** | Pew Research Center | 2010 | 2008, 2009 | Women reporting whether at least one daughter had FGM/C | NA | NA |
| **EMR** | **Djibouti, EVFF*** [25]** | l’Institut National de la Statistique (INSD) & Ministère de la Femme et de la Famille (MFF) | 2020 | 2019 | Women (15-24 years old), women of all ages and girls. | 0-14; 15-49 | Souna (Type I), Excision (Type II), Infibulation (Type III). |
|  | **Egypt, DHS[26]** | Ministry of Health and Population [Egypt], El-Zanaty and Associates [Egypt] & ICF | 2015 | 2014 | Women of reproductive age and girls. | 0-19; 15-49 | NA |
|  | **Iraq, MICS[27]** | Central Statistical Organization | 2019 | 2018 | Women of reproductive age and girls. | 0-14; 15-49 | Flesh removed, Nicked, Sewn closed. |
|  | **Somalia, SHDS[28]** | Directorate of National Statistics | 2020 | 2018-2019 | Women of reproductive age and girls. | 0-14; 15-49 | Sunni (Type I), Intermediate (Type II), Pharaonic (Type III or IV). |
|  | **Sudan, MICS[29]** | Central Bureau of Statistics (CBS) & UNICEF Sudan | 2016 | 2014 | Women of reproductive age and girls | 0-14; 15-49 | Flesh removed, Nicked, Sewn closed. |
|  | **Yemen, DHS[30]*** | Ministry of Public Health and Population (MOPHP), Central Statistical Organization (CSO) [Yemen], Pan Arab  Program for Family Health (PAPFAM), and ICF International. | 2015 | 2013 | Women of reproductive age and their daughters. | 0-14; 15-49 | Cut with flesh removed, Cut with no flesh removed. |
| **SEAR** | **Maldives, DHS[31]** | Ministry of Health (MOH) [Maldives] and ICF. | 2018 | 2016- 2017 | Women of reproductive age and girls. | 0-14; 15-49 | NA |
|  | **Indonesia, RISKESDAS[32]*** | Health Research and Development Agency | 2013 | 2013 | Girls. | 0-11 | NA |

Abbreviations: AFR: African Region; DHS: Demographic and Health Survey; EMR: Eastern Mediterranean Region; MICS: Multiple Indicator Cluster Surveys; SEAR: South-East Asia Region; SHDS: Somali Health and Demographic Survey; WHO: World Health Organization.

*Not included in meta-analysis for girls.

**Includes countries in AFR and EMR.

***EVFF: L’enquête nationale sur les violences faites aux femmes (National survey on violence against women).

**References**

1. Institut national de la statistique et de l’analyse économique (INSAE). Enquête par grappes à indicateurs multiples 2014, Rapport final. Cotonou, Bénin: UNICEF, 2015.

2. Institut National de la Statistique et de la Demographie IBF, International ICF. Burkina Faso Enquete Demographique et de Sante et ‡ Indicateurs Multiples (EDSBF-MICS IV) 2010. Calverton, Maryland, USA: Institut National de la Statistique et de la Demographie - INSD/Burkina Faso and ICF International, 2012.

3. Institut Centrafricain des Statistiques et des Etudes Economiques et Sociales. Central African Republic Multiple Indicator Cluster Survey 2018-2019 Bangui, République Centrafricaine: UNICEF, 2021.

4. INSEED and UNICEF. Enquête par grappes à indicateurs multiples Tchad 2019 Rapport final. N’Djamena, Tchad: UNICEF, 2021.

5. Institute National de la Statistique. Enquête par grappes à indicateurs multiples - Côte d’Ivoire 2016. Cote D’Ivoire: UNICEF, 2017.

6. Central Statistical Agency & ICF. Ethiopia Demographic and Health Survey 2016. Addis Ababa, Ethiopia: CSA and ICF, 2017.

7. National Statistics Office Fafo Institute For Applied International Studies. Eritrea Population and Health Survey 2010. Asmara, Eritrea: World Health Organisation, 2013.

8. Gambia Bureau of Statistics. The Gambia Demographic and Health Survey 2019-20. Banjul, The Gambia: The DHS Program ICF Rockville, Maryland, USA, 2021.

9. Ghana Statistical Service. Multiple Indicator Cluster Survey (MICS2017/18), Survey Findings Report. Accra, Ghana: UNICEF, 2018.

10. Institut National de la Statistique Ministère du Plan et du Développement Economique & ICF. République de Guinée Enquête Démographique et de Santé 2018. Conakry, Guinée: The DHS Program, ICF Rockville, Maryland, USA, 2019.

11. Ministério da Economia e Finanças Direcção-Geral do Plano Instituto Nacional de Estatistica. Inquérito aos Indicadores Múltiplos (MICS6) 2018-2019, Relatório Final. Bissau, Guiné-Bissau: UNICEF, 2020.

12. Kenya National Bureau of Statistics & ICF. Kenya Demographic and Health Survey 2014. Rockville, MD, USA: Kenya National Bureau of Statistics and ICF International, 2015.

13. Liberia Institute of Statistics and Geo-Information Services. Liberia 2019-2020 DHS. Freetown, Liberia: 2021.

14. Institut National de la Statistique (INSTAT) Cellule de Planification et de Statistique Secteur Santé-Développement Social et Promotion de la Famille (CPS/SS-DS-PF) & ICF. Mali Demographic and Health Survey 2018. Bamako, Mali: INSTAT/CPS/SS-DS-PF and ICF, 2019.

15. Mauritania National Statistics Office. Enquête par Grappes à Indicateurs Multiples, 2015, Résultats clés. Nouakchott, Mauritanie: UNICEF, 2016.

16. National Population Commission (NPC) [Nigeria] & ICF. Nigeria Demographic and Health Survey 2018 - Final Report. Abuja, Nigeria: NPC and ICF, 2019.

17. Institut National de la Statistique INSN, International ICF. Niger Enquéte Demographique et de Santè et ‡ Indicateurs Multiples (EDSN-MICS IV) 2012. Calverton, Maryland, USA: INS/Niger and ICF International, 2013.

18. Agence Nationale de la Statistique et de la Démographie (ANSD). Enquête Démographique et de Santé Continue - EDS-Continue 2019. Dakar, Sénégal: The DHS Program ICF Rockville, Maryland, USA 2020.

19. Statistics Sierra Leone & ICF. Sierra Leone Demographic and Health Survey 2019. Freetown, Sierra Leone: Ministry of Health and Sanitation and ICF International, 2020.

20. Ministry of Health Tanzania and Zanzibar National Bureau of Statistics Office of Chief Government Statistician Zanzibar ICF. Tanzania Demographic and Health Survey and Malaria Indicator Survey 2015-2016. Dar es Salaam, Tanzania: MoHCDGEC, MoH, NBS, OCGS, and ICF, 2016.

21. Institut National de la Statistique et des Etudes Economiques et Démographiques. Togo - Enquête à Indicateurs Multiples 2017, Rapport final. Lomé, Togo: UNICEF, 2018.

22. Uganda Bureau of Statistics and ICF. Uganda Demographic and Health Survey 2016. Kampala, Uganda: UBOS and ICF, 2018.

23. Central Statistical Office (CSO) Ministry of Health (MOH) University of Zambia and MEASURE Evaluation. Zambia Sexual Behaviour Survey 2009. Lusaka, Zambia: CSO and MEASURE Evaluation, 2010.

24. Pew Research Center. Tolerance and Tension: Islam and Christianity in Sub-Saharan Africa. Pew Research Center, 2010.

25. l’Institut National de la Statistique (INSD) et Ministère de la Femme et de la Famille (MFF). Rapport de l’enquete nationale sur les mutilations genitales feminines et les violences faites aux femmes Djibouti: FNUAP, UNICEF, EU, 2020.

26. Ministry of Health Population El Zanaty and associates and ICF International. Egypt Demographic and Health Survey 2014. Cairo, Egypt: Ministry of Health and Population and ICF International, 2015.

27. Central Statistical Organization. Iraq Multiple Indicator Cluster Survey 2018 Survey Findings Report. Iraq: UNICEF, 2019.

28. Directorate of National Statistics. Somalia Health and Demographic Survey 2020. Federal Government of Somalia, United Nations Population Fund, 2020.

29. Central Bureau of Statistics & UNICEF Sudan. Multiple Indicator Cluster Survey 2014 of Sudan, Final Report. Khartoum, Sudan: UNICEF, 2016.

30. Ministry of Public Health and Population. Yemen National Health and Demographic Survey 2013. Rockville, Maryland, USA: MOPHP, CSO, PAPFAM, and ICF International, 2015.

31. Ministry of Health and ICF. Maldives Demographic and Health Survey 2016-17. Malé, Maldives: MOH and ICF, 2018.

32. Health Research and Development Agency. Riset Kesehatan Dasar (RISKESDAS) 2013. Indonesia: Government of Indonesia, 2013.
