# Supplementary material for: The global prevalence of female genital mutilation/cutting: A systematic review and meta-analysis of national, regional, facility, and school-based studies
Source: PLoS Med. 2022 Sep 1;19(9):e1004061. doi: 10.1371/journal.pmed.1004061 (PMC9436112; doi:10.1371/journal.pmed.1004061)
Supplement: S1 Text — (DOCX) [file pmed.1004061.s005.docx]

**S1 Text. Inclusion and Exclusion Criteria**

Inclusion criteria:

1. Reported on FGM/C prevalence using population-based methods (cross-sectional or cohort studies) at the national or subnational level or examined FGM/C in facility
2. Or non population-based studies examining FGM/C which may include case series, regional studies, facility-based studies or school-based studies.
3. or examined the risk factors of FGM/C within a population (cross-sectional, case-control or cohort studies). To meet the last criterion the comparison had to occur between women or girls with FGM/C to those without FGM.

Exclusion criteria:

1. Only reported health impacts of FGM/C, healthcare providers, policy, economic effects, or perceptions,
2. Only used qualitative methods
3. Were systematic reviews (except for the purpose of referencing)
4. Conference proceedings and letters to the editor.

[Studies only meeting the last inclusion criterion (iii. risk factors or determinants of FGM/C) were excluded from this reported study on prevalence but were included in the wider project]
