# Supplementary material for: The global prevalence of female genital mutilation/cutting: A systematic review and meta-analysis of national, regional, facility, and school-based studies
Source: PLoS Med. 2022 Sep 1;19(9):e1004061. doi: 10.1371/journal.pmed.1004061 (PMC9436112; doi:10.1371/journal.pmed.1004061)
Supplement: S2 Text — (DOCX) [file pmed.1004061.s006.docx]

**S2 Text. Supplementary results.**

Egger’s test results showed P=0.99, and this did not indicate funnel plot asymmetry or bias among nationally representative surveys on the prevalence of FGM/C amongst women (Supplementary Figure 1). Egger’s test results showed P=0.012, indicating funnel plot asymmetry and implying bias among nationally representative surveys on the prevalence of FGM/C amongst girls (Supplementary Figure 2). In addition, the funnel plots visually show high heterogeneity, although it is likely that this is due to different prevalence rates in different countries rather than publication bias.
