# Supplementary figures and images for: The global prevalence of female genital mutilation/cutting: A systematic review and meta-analysis of national, regional, facility, and school-based studies
Source: PLoS Med. 2022 Sep 1;19(9):e1004061. doi: 10.1371/journal.pmed.1004061 (PMC9436112; doi:10.1371/journal.pmed.1004061)

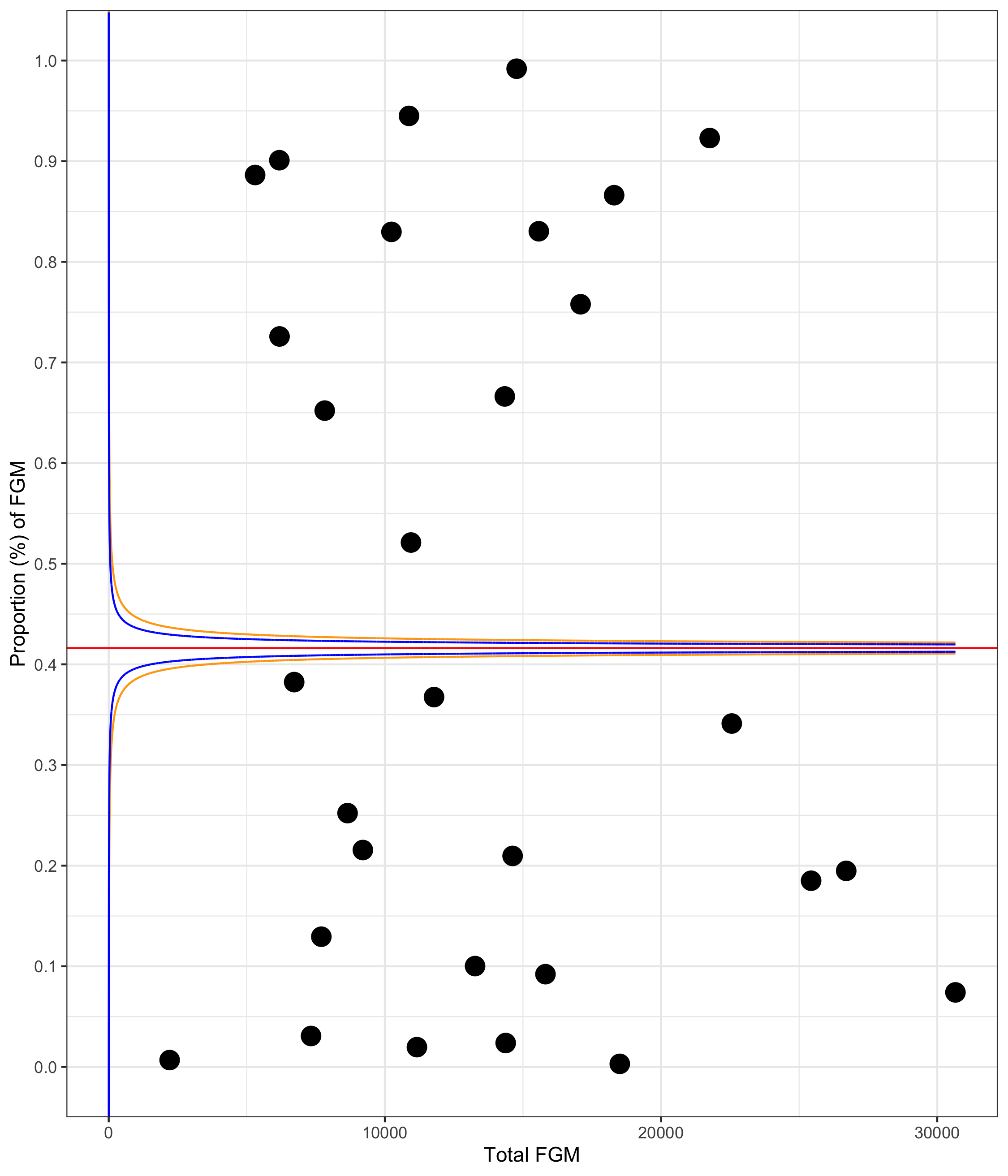

Supplement: S1 Fig — (TIF) [file pmed.1004061.s008.tif]

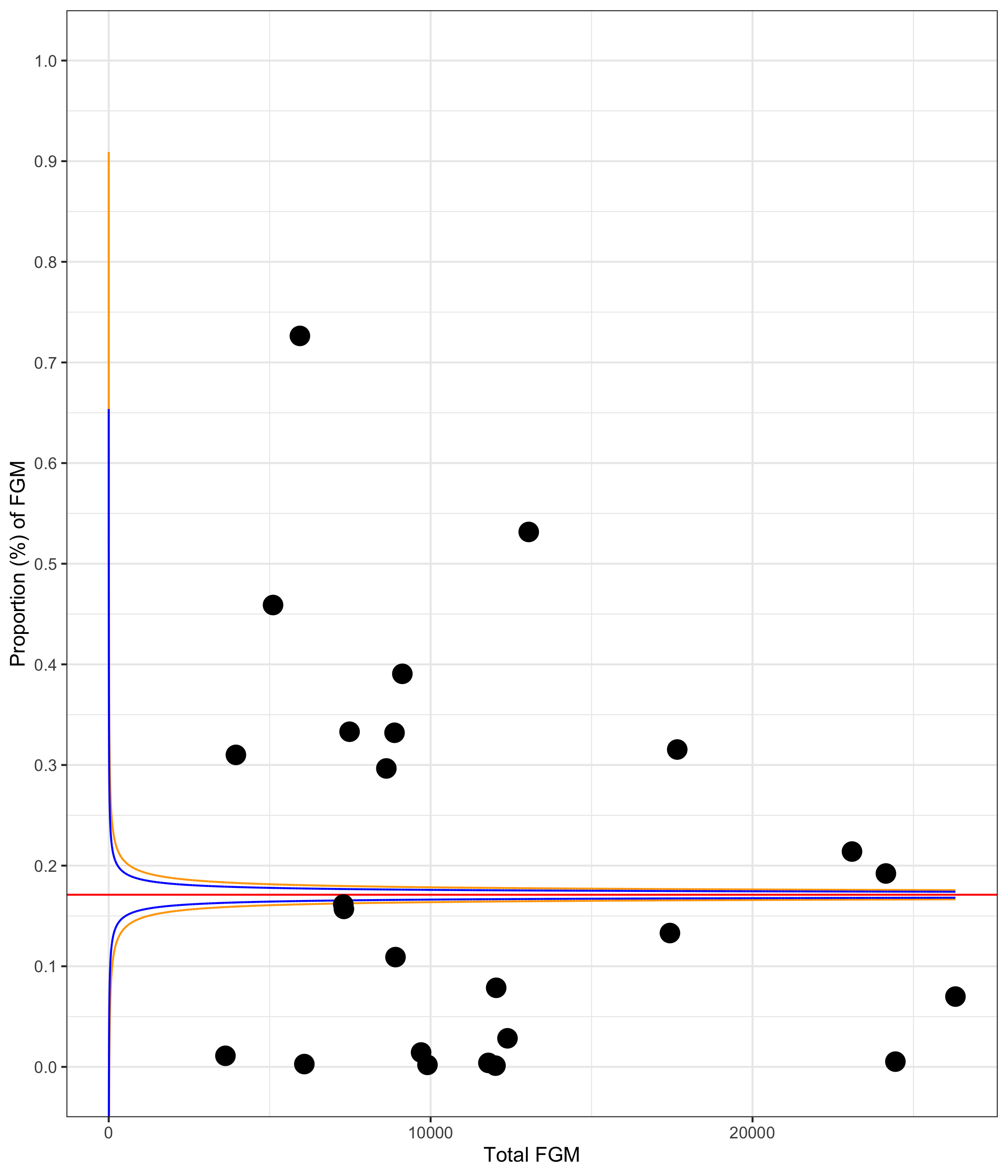

Supplement: S2 Fig — (TIF) [file pmed.1004061.s009.tif]
