# Supplementary material for: The global prevalence of female genital mutilation/cutting: A systematic review and meta-analysis of national, regional, facility, and school-based studies
Source: PLoS Med. 2022 Sep 1;19(9):e1004061. doi: 10.1371/journal.pmed.1004061 (PMC9436112; doi:10.1371/journal.pmed.1004061)
Supplement: S1 Appendix — Table A. Characteristics of subregional population-based studies. All studies used cross-sectional methods. Table B. Prevalence of FGM/C in women and girls in subregional population-based studies. *Women reported that at least 1 daughter had FGM/C in the household. †Youngest daughter had FGM/C. ‡Due to inconsistent data reported in the study, this number was calculated by the authors of this review. **Total FGM/C and sample size for women and girls were excluded due to conflicting numbers within the report. Table C. Types of FGM/C in subregional population-based studies. *% of women; †% of youngest daughter; ¶At least 1 living daughter; ‡% of girls. #Pharaonic (Type III or IV); Northeast Zone MICS calculates the prevalence of type out of the total number of participants. Table D. Characteristics of FGM/C procedure in subregional population-based studies. *The report was unclear about the percentages of the performers of FGM/C in women and daughters. (DOCX) [file pmed.1004061.s010.docx]

**S1 Appendix: Sub-Regional Studies**

**Table A.** Characteristics of Sub-Regional Representative Studies

|  | **Author** | **Year** | **Date of Data collection** | **Sampling Method** | **Sub region** | **Population Description** | **Ethnicity** | **Age** | **Data Collection Site** | **Types of FGM/C** | **Risk of Bias** |
| --- | --- | --- | --- | --- | --- | --- | --- | --- | --- | --- | --- |
| **South Africa** | Scorgie [1] | 2010 | 2007 | Multi-stage cluster | KwaZulu-Natal | Women | NA | 18-60 | Household | NA | Mod. |
| **Burkina Faso** | Greis[2] | 2020 | 2017 | Two-stage stratified (Villages)  Random (Participants) | 10 villages and one sector of Nouna town. | Young women and girls | Bwaba (36.1%), Dafin (21.7%), Mossi (19.8%), Peulh (11%) , Samo (9.5%), other (1.9%) | 12-20 | Village | Flesh removed, Nicked, Sewn closed | Low |
|  | Komboigo[3] | 2019 | 2016 | Multi-staged cluster sampling | Ouagadougou | Women living with a partner | NA | 16-65 | Community site | NA | Mod. |
| **Ethiopia** | Mitike [4] | 2009 | 2004 | Systematic random sampling | Somali Regional State in Eastern Ethiopia. Aysha refugee camp (45.1%), Kebribeyah (42.7%), Hartishek (12.2%). | Refugees from Somalia | Somali (100%) | 1-12 | Household | Clitoral cutting, Vaginal sewing | Mod. |
|  | Yirga  [5] | 2012 | 2008 | Systematic random sampling | Kersa district, East Hararge, Oromia region, Ethiopia | Women of reproductive age reporting on daughters. | Oromo (95.7%), Amhara (4.2%), Gurage (0.1%) | 15-49 | Household | Clitoris only; Clitoris, labia; Clitoris, labia, and surrounding part; Clitoris, labia, surrounding part and stitching; other forms | Mod. |
|  | Gajaa[6] | 2016 | 2014 | Two stage systematic random sampling | Hababo Guduru District | Women of reproductive age and girls | NA | 0-14, 15-49 | District | NA | Low |
|  | Oljira[7] | 2016 | 2013 | Two stage systematic random sampling | Harar, Eastern Ethiopia | Women and daughters | Oromo (39.2%), Harari (5.0%), Amhara (37.8%), Tigre (4.6%), Gurage (8.9%), Other (4.5%) | 0-12 (girls), 15-50+ (mothers) | Household | Removal of flesh, Cut without removal of flesh, Stitched with or without removing flesh | Mod. |
|  | Gebrekirstos[8] | 2014 | 2013 | Multi-stage random sampling | Axum town, North Ethiopia | Mothers and children under 5 | NA | <5, 15-45+ | Community | NA | Mod. |
|  | Andualem [9] | 2016 | 2014 | Systematic random sampling | East Gojjam Zone, Western Amhara | Mothers and infants | Amhara (97%), Others, (3%) | 15-49, <5 | Household | 1,2 | Low |
|  | Bogale[10] | 2014 | 2014 | Stratified random sampling | Bale zone | Women of reproductive age and daughters | Oromo (87.2%), Amhara (12.8%) | 15-49 (women) | Community | 1,2,3 | Low |
|  | Sood[11] | 2022 | 2021 | Multi-stage sampling; random stratified sample of clusters | Afar and Southern Nations, Nationalities and Peoples (SNNP), Addis Ababa City Administration. | Households with adolescent girl and a primary caregiver in the household | NA | 10-19 (girls), and older women | Household | NA | Mod. |
|  | Abebe[12] | 2020 | 2020 | Multi-stage sampling; Systematic random sampling | Afar and Amhara | Women with daughters under 15 years old | Afar (47.4%), Amhara (49.6%), Oromo (3%) | 15-49 (women), 0-14 (girls) | Household | NA | Low |
|  | Melese[13] | 2020 | 2018 | Multi-stage sampling; Systematic random sampling | Degadamot district, Amhara regional state, Northwest Ethiopia | Women with daughters under 5 years old | Amhara (100%) | 0-5 Girls | Household | NA | Low |
|  | Gudeta[14] | 2022 | 2019 | Multi-stage cluster sampling | Keffa Zone, Southwest Ethiopia | Mothers with daughters younger than 15 years | NA | Mothers 15-49, daughters under 15 years | Community | Nicked, Flesh removed | Low |
| **Kenya** | Mudege[15] | 2012 | 2007 -2008 | Population Based (Every household was visited) | Korogocho and Viwandani , 2 informal settlements in Nairobi, Kenya | Girls and women in informal settlements | Kikuyu (36.5%), Kisii (13.8%), Somali (12.9%), Garre (14.2%), Borana (11.9%), Other (17.3%) | 12-24 | Household | NA | Low |
| **Nigeria** | Ifeanyichukwu[16] | 2015 | 2014 | Cluster Sampling | Okada Community, Edo State | Women of reproductive age | Benin (36.3%), Yoruba (15.7%), Esan (15.4%), Igbo (12.0%), Urhobo (10.5%), others: Ijaw, Efik, Igala and Ogoja (10.2%) | 15-49 | Community | NA | Low |
|  | Alo[17] | 2011 | 2010 | Stratified random sampling | Southwest Nigeria | Mothers with at least one circumcised daughter | NA | 15-49 | Household | NA | Mod. |
|  | Adeyinka[18] | 2009 | 2007 | Multi-stage sampling | Igbo-Ora town, Oyo state, Southwestern Nigeria | Adult women | Yoruba (94.4%), Ibo (2.5%), Hausa (1.8%), Others (0.7%) | 18+ (18 to 80 years) | Household | NA | Low |
|  | Johnson[19] | 2012 | NA | Multi-stage (location) and random (participants) | 3 villages in Itu, Akwa Ibom state, southern Nigeria | Women | Ayadehe 100% | <20 – 40+ | Household | 1, 2, unclear | Low |
| **Tanzania** | Galukande[20] | 2015 | 2013-2014 | Multi-stage sampling; Systematic random sampling | Arusha | Women | Masai (predominant) | 18+ Mean 34 years | Household | NA | Low |
| **Sierra Leone** | Bjälkander[21] | 2012 | 2009-2010 | Simple random sampling (streets), systematic random sampling (households),  purposive (sampling participants) | Bombali and Port Loko Districts in Northern Sierra Leone | Young women and girls | Fulah (3.5%), Kono (2.0%), Korankoh (0.6%), Limba (15.5%), Loko (23.2%), Madingo (2.0%), Mende (0.6%), Temne (52.6%) | 10-20 | Household | NA | Mod. |
| **Mali** | Diabate[22] | 2019 | 2009 | Cross sectional Household Survey -Enquête Nationale sur l’Excision au Mali | Kayes, Koulikoro, Sikasso, Segou, Mopti, and Bamako | Girls | Girls living in localities | 0-8 | Household | NA | High |
| **Somalia** | Gele[23] | 2013 | 2011 | Systematic random sampling | Hargeisa district | Women | NA | 18+  31 (Mean) | Household | 1,2,3 | Mod. |
|  | Central Statistics Department, Ministry of Planning and National Development, Somaliland Government[24] | 2020 | 2018-2019 | Multi-stage cluster sampling | Somaliland | Women and girls | NA | 0-14, 15-49 | Household | Sunni, Intermediate, Pharaonic, sewn closed, not sewn closed | Low |
|  | UNICEF & Somalia Ministry of Planning & International Cooperation [25] | 2014 | 2011 | Multi-stage cluster sampling | Northeast Zone | Women and girls | NA | 0-14, 15-49 | Household | Flesh removed, nicked, sewn closed | Low |
| **Yemen** | Alosaimi[26] | 2019 | 2008-2009 | Multi-stage sampling | Alazareq (Dhale Governorate), Asabrah and Fara’a Aludain (Ibb Governorate), Mokaa and Maoza (Taiz Governorate), and Azidiah (Hodeidah Governorate). | Women of reproductive age. | NA | 15-49 | Household | NA | Low |
| **Iraq** | Abdulah[27] | 2019 | 2017 | Two stage random sampling | Iraqi Kurdistan region (Duhok, Erbil, and Sulaiymaniya) | Women of all ages | NA | All age groups | Household | NA | Mod. |
| **Egypt** | Ali [28] | 2018 | 2017 | Random cluster sampling (areas), random sampling (households) | Beni-Suef | Young women and girls. | NA | 12-25 | Household | NA | Mod. |
|  | Mohammed[29] | 2018 | 2016 | Multi-stage systematic random sampling | Rural area in Minia | Women | NA | 18+ | Household | NA | Low |
|  | Salama[30] | 2021 | NA | Multi-stage cluster sampling | Six governorates representing Egypt | Children | NA | 9 months – 16 years | Community | NA | Mod. |
|  | Zayed[31] | 2012 | NA | Random | Cairo & Giza | Women and girls | NA | 5-30 | Community | NA | Mod. |
| **Saudi Arabia** | Milaat[32] | 2018 | 2017 | Random cluster sampling (location), multi-stage random sampling (household) | Hali semi-urban region. | Girls | NA | ≤18 years | Household | NA | Mod. |

All studies used a cross-sectional design.

**Table B.** Prevalence of FGM/C in Women and Girls in Sub-Regional Representative Studies

|  | | | | | | | **Women** | | | **Girls** | | |
| --- | --- | --- | --- | --- | --- | --- | --- | --- | --- | --- | --- | --- |
|  | **Author** | **Year** | **Sub region** | **Sampling method** | **Population description** | **Age** | **Prevalence %** | **Total FGM/C** | **Sample Size** | **Prevalence %** | **Total FGM/C** | **Sample Size** |
| **EMR** | | | | | | | | | | | | |
| **Somalia** | Gele[23] | 2013 | Hargeisa district, Somalia | Systematic random sampling | Adult women | Mean: 31, Range: <25-41+ (includes males) | 97% | 104 | 107 |  |  |  |
|  | Central Statistics Department, Ministry of Planning and National Development, Somaliland Government [24] | 2020 | Somaliland | Multistage cluster sampling | Women and girls | 0-14, 15-49 | 98.1%** |  |  | 19.5%** |  |  |
|  | UNICEF & Somalia Ministry of Planning & International Cooperation [25] | 2014 | Northeast Zone | Multistage cluster sampling | Women and girls | 0-14, 15-49 | 98% | 5382 | 5492 | 30.6% | 1779 | 5,813 |
| **Yemen** | Alosaimi [26] | 2019 | Alazareq (Dhale Governorate), Asabrah and Fara’a Aludain (Ibb Governorate), Mokaa and Maoza (Taiz Governorate), and Azidiah (Hodeidah Governorate). | Multi-stage sampling | Women of reproductive age | 15-49 (mothers) | 47.8% | 3384 | 7076 | 34%* | 2405 | 7076 |
| **Egypt** | Ali[28] | 2018 | Beni-Suef | Random cluster sampling (areas), random sampling (households) | Young women and girls | 12-25 | 55% | 1846 | 3353 |  |  |  |
|  | Mohammed [29] | 2018 | Rural area in Minia | Multi-stage systematic random sampling | Women | 18+ | 76.6% | 320 | 418 |  |  |  |
|  | Zayed[31] | 2012 | Cairo & Giza | Random | Women and girls | 5-30 | 63.9% | 156 | 244 |  |  |  |
|  | Salama[30] | 2021 | Six governorates representing Egypt | Multi-stage cluster sampling | Children | 9 months – 16 years |  |  |  | 29.7% | 508 | 1,710 |
| **Iraq** | Abdulah[27] | 2019 | Iraqi Kurdistan region( Duhok, Erbil, and Sulaiymaniy) | Two stage random sampling | Women | All age groups | 46.8% | 2361 | 5048 |  |  |  |
| **Saudi Arabia** | Milaat[32] | 2018 | Hali semi-urban region | Random cluster sampling (location), multi-stage random sampling (household) | Girls | ≤18 years |  |  |  | 80.3% | 175 | 218 |
| **AFR** | | | | | | | | | | | | |
| **Tanzania** | Galukande[20] | 2015 | Arusha | Multi-stage sampling; Systematic random sampling | Women | 18+ Mean 34 years | 69.2% | 467 | 675 |  |  |  |
| **South Africa** | Scorgie[1] | 2010 | KwaZulu-Natal | Multi-stage cluster | Women | 18-60 | 3% | 26 | 867 |  |  |  |
| **Nigeria** | Ifeanyichukwu[16] | 2015 | Okada Community, Edo State | Cluster Sampling | Women of reproductive age | 15-49 | 28.7% | 90 | 314 |  |  |  |
|  | Alo[17] | 2011 | Southwest Nigeria | Stratified random sampling | Mothers with at least one daughter circumcised | 15-49 | 75% | 315 | 420 | 71%* | 298 | 420 |
|  | Adeyinka [18] | 2009 | Igbo-Ora town, Oyo state, Southwestern Nigeria | Multi-stage sampling | Adult women | 18+ | More than 78.7% | 118 | 150 |  |  |  |
|  | Johnson [19] | 2012 | 3 villages that make up the Ayadehe clan in Itu local govt. area in Akwa Ibom state, southern Nigeria | Multi-stage (location) and random (participants) | Women | <20 – 40+ | 92.7% | 202 | 218 |  |  |  |
| **Ethiopia** | Oljira[7] | 2016 | Harar, Eastern Ethiopia | Two stage systematic random sampling | Women and girls | 0-12 (daughters)  15+ (mothers) | 79.5% | 669 | 842 | 19%* | 160 | 842 |
|  | Gajaa[6] | 2016 | Hababo Guduru District | Two stage systematic random sampling | Women of reproductive age and daughters | Women aged 15-49, Girls aged 0-14 | 98.2% | 599 | 610 | 48%* | 293 | 610 |
|  | Gebrekirstos[8] | 2014 | Axum town, North Ethiopia | Multi-stage random sampling | Children under 5 and their mothers | <5  15+ (mothers) | 0.7% | 5 | 746 | 0% | 0 | 752 |
|  | Yirga[5] | 2012 | Kersa district, East Hararge, Oromia region | Systematic random sampling | Women of reproductive age | 15-49 | 92.3% | 792 | 858 | 88.1%* | 288 | 327 |
|  | Andualem [9] | 2016 | East Gojjam Zone, Western Amhara | Systematic random sampling | Mothers and infants | Mothers 15-49, daughters 0-2 | 96% | 689 | 718 | 49% | 403 | 805 |
|  | Bogale[10] | 2014 | Bale zone | Stratified random sampling | Women of reproductive age and daughters | 15-49 (mothers) | 78.5% | 486 | 619 | NA | 150† | NA |
|  | Mitike[4] | 2009 | Aysha, Kebribeyah and Hartishek refugee camps, Somali Regional State in Eastern Ethiopia | Systematic random sampling | Refugees from Somalia | 1-12 |  |  |  | 42.4% | 122 | 288 |
|  | Sood [11] | 2022 | Afar and Southern Nations, Nationalities and Peoples (SNNP), Addis Ababa City Administration. | Multi-stage sampling; random stratified sample of clusters | Households with adolescent girl and a primary caregiver in the household | 10-19 (girls), and older women | 65% women and girls combined |  |  |  |  |  |
|  | Abebe[12] | 2020 | Afar and Amhara | Multi-stage sampling; Systematic random sampling | Women with daughters under 15 years old | 15-49 (women), 0-14 (girls) | 98% | 398 | 405 | 75.9%‡ | 796 | 1048 |
|  | Melese[13] | 2020 | Degadamot district, Amhara regional state, Northwest Ethiopia | Multi-stage sampling; Systematic random sampling | Women with daughters under 5 years old | 0-5 Girls |  |  |  | 70.80% | 230 | 325 |
|  | Gudeta[14] | 2022 | Keffa Zone, Southwest Ethiopia | Multi-stage cluster sampling | Mothers with daughters younger than 15 years | Mothers 15-49, daughters under 15 years | 21.2% | 159 | 750 | 1.6% | 12 | 750 |
| **Burkina Faso** | Greis[2] | 2020 | 10 villages and one sector of Nouna town | Two-stage stratified (Villages)  Random (Participants) | Young women and girls | 12-20 | 43.2% | 301 | 696 |  |  |  |
|  | Komboigo[3] | 2019 | Ouagadougou | Multi-Stage cluster | Women living with a partner | 16-65 | 53.4% | 307 | 575 |  |  |  |
| **Kenya** | Mudege [15] | 2012 | Korogocho and Viwandani , 2 informal settlements in Nairobi, Kenya | Population based | Young women and girls in informal settlements | 12-24 | 61.3% | 323 | 527 |  |  |  |
| **Sierra Leone** | Bjälkander [21] | 2012 | Bombali and Port Loko Districts in Northern Sierra Leone | Simple random sampling (streets), systematic random sampling (households),  purposive (sampling participants) | Young women and girls | 10-20 | 61.1% | 189 | 310 |  |  |  |
| **Mali** | Diabate[22] | 2019 | Kayes, Koulikoro, Sikasso, Segou, Mopti, and Bamako | Unclear (Original survey is unavailable). Stated as representative according to the report. | Girls living in localities | 0-8 |  |  |  | 71.4% | 1413 | 1979 |

* Women reported that at least 1 daughter had FGM/C in the household. † Youngest daughter had FGM/C. ‡ Due to inconsistent data reported in the study, this number was calculated by the authors of this review.

** Total FGM/C and sample size for women and girls were excluded due to conflicting numbers within the report.

Abbreviations: AFR: African Region EMR: Eastern Mediterranean Region, FGM/C: Female Genital Mutilation/Cutting

**Table C.** Types of FGM/C in Sub-Regional Representative Studies

| **Region** | **Author** | **Year** | **Country** | **Prevalence Women (%)** | **Total FGM/C Women** | **Prevalence Girls (%)** | **Total FGM/C Girls** | **Type 1 (%)** | **Type 2 (%)** | **Types 1 or 2 (%)** | **Type 3 (%)** | **Type 4 (%)** | **Other Type** | **Don't Know/Missing Type** |
| --- | --- | --- | --- | --- | --- | --- | --- | --- | --- | --- | --- | --- | --- | --- |
| AFR | Andualem [9] | 2016 | Ethiopia | 96% | 689 | 49% | 403^‡^ | 48.9^‡^ | 51.1%^‡^ |  |  |  |  |  |
|  | Bogale[10] | 2014 | Ethiopia | 78.5% | 486 | NA | 150^†^ | 2.5%*, 4.7%^†^ | 78.6%*, 87.3%^†^ |  | 7.8%*, 8%^†^ |  | 11.1%* |  |
|  | Gudeta[14] | 2022 | Ethiopia | 21.2% | 159 | 1.6% | 12^‡^ |  |  | Type I and II reported together: 83%^‡^, 83.3% ^‡^ |  | 61%*, 16.7% ^‡^ |  |  |
|  | Johnson [19] | 2012 | Nigeria | 92.7% | 202 |  |  | 7.9%* | 71.2%* |  |  |  |  | 20.9%* |
|  | Greis[2] | 2020 | Burkina Faso | 43.2% | 301 |  |  |  |  | Flesh removed (Type I or II): 69.6%* | 2%* | 20.8%* |  |  |
|  | Mitike[4] | 2009 | Ethiopia |  |  | 42.4% | 122^‡^ |  |  |  | 36.1%^‡^ |  | Partial or full clitoral cutting: 63.9%^‡^ |  |
|  | Oljira[7] | 2016 | Ethiopia | 79.5% | 669 | 19% | 160^¶^ |  |  | Removal of any flesh: 97.5% (Type I or II) ^¶^ | 5.6%^¶^ | 1.9%^¶^ |  |  |
| EMR | Gele[23] | 2013 | Somalia | 97% | 104 |  |  |  |  | Type I and II reported together: 15.9%* | 81.3%* |  |  |  |
| EMR | Central Statistics Department, Ministry of Planning and National Development, Somaliland Government [24] | 2020 | Somaliland | 98.1% |  | 19.5% |  | 29.2%* | 7.3%* |  | 60.7%^*#^, 39.1%^‡^ |  | Not sewn closed: 59.9%^‡^ | 2.8%*,1.0%^‡^ |
|  | UNICEF Somalia [25] | 2014 | Somalia  (Northeast Zone) | 98% | 5382 | 30.6% | 1779^‡^ |  |  | Flesh removed: 5.7%*, 4.7%^‡^ | 86.7%*, 22.5%^‡^ | 1.4%*, 0.9%^‡^ |  | 4.2%*, 2.5%^‡^ |

* % of Women. ^†^ % of youngest daughter. ^¶^ At least one living daughter. ^‡^ % of girls. ^#^ Pharaonic (Type III or IV). Northeast Zone MICS calculates the prevalence of type out of the total number of participants.

Abbreviations: FGM/C: Female Genital Mutilation/Cutting.

**Table D.** Characteristics of FGM/C Procedure in Sub-Regional Representative Studies

| **Country** | **Author** | **Year** | **Age at FGM/C** | **Performer of FGM/C** | **Location of Procedure** |
| --- | --- | --- | --- | --- | --- |
| **AFR** | | | | | |
| **Burkina Faso** | Greis[2] | 2020 | 0–4 years (54.0%), 5–9 years (39.5%), 10–14 years (6.2%), 15+ years (0.3%) | Traditional (97.4%), Medical (1.1%), Other (1.6%) |  |
| **Ethiopia** | Oljira[7] | 2016 | Daughters: <1 (20.6%), 1-4 (5.6%), 5-8 (67.5%) ,9-12 (5.6%), don’t know (0.6%) | Relatives, neighbors, and health personnel (5.6%), Traditional (94.4%) |  |
|  | Gajaa[6] | 2016 | Daughters: 1-4 (2%), 5-8 (20.7%), 9-12 (23.1%), 13-15 (1.6%) | Traditional (92.8%) |  |
|  | Mitike[4] | 2009 | Mean age: the first daughter (older) 7.5 (± 1.7), last (younger) daughter 6.5 (± 1.4) years. Optimum age: 5–8 years old | Traditional (99.1%), Medical (0.9%) |  |
|  | Yirga[5] | 2012 |  | Traditional (94.1%) (76.1% by local healers and 18% by elderly people) |  |
|  | Andualem[9] | 2016 |  | Traditional (100%) | Home (96%), Circumciser's home (4%) |
|  | Abebe[12] | 2020 | <1 year (72%) | Traditional cutters (72.8%), Traditional birth attendant (8.9%), other (0.9%), don’t know (17.3%) |  |
|  | Gudeta[14] | 2022 | Mother's age at circumcision: I don’t know (45.3%); 5–9 (30.8%); 10–14 (18.2%); ≥15 (5.7%). Daughters: <10 years (83.3%); ≥10 years (16.7%) | Mothers: Traditional (98.7%), health professional (1.3%) Daughters: Traditional (100%) |  |
|  | Bogale[10] | 2014 | Mean 7.89 (SD ± 4.56). |  |  |
| **Kenya** | Mudege[15] | 2012 | Mean: 10, Median: 13 |  |  |
| **Nigeria** | Ifeanyichukwu[16] | 2015 | Mean 4.12 ± 1.17, 0-5 years (83.4%) | Traditional (67.7%), Medical (43.3%) | Home (40%)  Traditional home (33.3%)  Health facility (26.7%) |
|  | Johnson [19] | 2012 | < 6 (5.4%), 6-12 (69.8%), 13-18 (23.3%), 18+ (1.5%) | Traditional (53.5%), Medical (0.5%), Native Doctor (unclear if medically trained) (46%) | Home (99.5%) |
| **Sierra Leone** | Bjälkander[21] | 2012 | 0 – 1 (1.6%), 2 – 4 (7.4%), 5 – 9 (21.3%), 10 – 14 (21.9%), 15+ (2.9%), Don’t Know (5.8%) | Traditional (85.7%), Medical (13.2%), Nurse & Sowei (traditional) together (0.5%), don't know (0.5%) |  |
| **Tanzania** | Galukande[20] | 2015 | ≤ 11 years 107 (15.8%) 12–15years 169 (25%) ≥ 16 years 143 (21.2%) |  |  |
| **Mali** | Diabate[22] | 2019 | <1 year (22.9%), 1 (~5%), 2 (~5%), 3 (~ 5%). 6+ (0%). |  |  |
| **EMR** | | | | | |
| **Egypt** | Mohammed[29] | 2018 | Range 4–17, (mean ± SD) 11.5 ± 2.3 | Traditional (91.3%), Medical (8.7%) |  |
|  | Salama[30] | 2021 |  | Medical professionals (76%), Midwife or Daya (20.1%), don’t’ know (3.9%) | Private clinic (40.6%)  Home (59.4%) |
|  | Zayed[31] | 2012 | Mean 10.846 (SD ± 1.98) years and ranged between 8-15 | Traditional (33.3%), Medical (64.1%), Other (2.6%) | Home (56.5%), Clinic (38.5%)  Hospital (5%) |
| **Saudi Arabia** | Milaat[32] | 2018 | 7 or less (59.4%), 8-10 (0.6%), 11-14 (2.9%), 15-17 (1.7%), 18 (35.4%) | Traditional (2.8%), Medical (97.1%) |  |
| **Somalia** | Central Statistics Department, Ministry of Planning and National Development, Somaliland Government [24] | 2020 | For women: 5-9 (57.0%), 10-14 (41.2%), 15+ (1.1%), don’t know (0.1%) | For women: Traditional (78.0%), Medical (21.8%), Don’t know (0.4%)*  For : Traditional (74.9%), Medical (24.5%), Don’t know (0.5%)* |  |

* The report was unclear about the percentages of the performers of FGM/C in women and daughters.

Abbreviations: AFR: African Region; EMR: Eastern Mediterranean Region; FGM/C: Female Genital Mutilation/Cutting

**References**

1. Scorgie F, Beksinska M, Chersich M, Kunene B, Hilber AM, Smit J. "Cutting for love": Genital incisions to enhance sexual desirability and commitment in KwaZulu-Natal, South Africa. Reproductive Health Matters. 2010;18(35):64-73. doi: 10.1016/S0968-8080(10)35500-5.

2. Greis A, Barnighausen T, Bountogo M, Ouermi L, Sie A, Harling G. Attitudes towards female genital cutting among adolescents in rural Burkina Faso: a multilevel analysis. Tropical Medicine & International Health. 2020;25(1):119-31. PubMed PMID: 31698528.

3. Komboigo BE, Kiemtoré S, Kain DP, Zamané Y, Kaboré X, Zoundi M, et al. Évaluation des dysfonctions sexuelles de la femme vivant en couple de la ville de Ouagadougou, Burkina Faso. Medecine et sante tropicales. 2019;29(3):310-6. doi: 10.1684/mst.2019.0932.

4. Mitike G, Deressa W. Prevalence and associated factors of female genital mutilation among Somali refugees in eastern Ethiopia: a cross-sectional study. BMC Public Health. 2009;9:264. PubMed PMID: 19635149.

5. Yirga WS, Kassa NA, Gebremichael MW, Aro AR. Female genital mutilation: prevalence, perceptions and effect on women's health in Kersa district of Ethiopia. International Journal of Women's Health. 2012;4:45-54. PubMed PMID: 22371659.

6. Gajaa M, Wakgari N, Kebede Y, Derseh L. Prevalence and associated factors of circumcision among daughters of reproductive aged women in the Hababo Guduru District, Western Ethiopia: a cross-sectional study. BMC Women's Health. 2016;16:42. PubMed PMID: 27449648.

7. Oljira T, Assefa N, Dessie Y. Female genital mutilation among mothers and daughters in Harar, eastern Ethiopia. International Journal of Gynaecology & Obstetrics. 2016;135(3):304-9. PubMed PMID: 27609740.

8. Gebrekirstos K, Abebe M, Fantahun A. A cross sectional study on factors associated with harmful traditional practices among children less than 5 years in Axum town, north Ethiopia, 2013. Reproductive Health. 2014;11:46. PubMed PMID: 24952584.

9. Andualem M. Determinants of Female Genital Mutilation Practices in East Gojjam Zone, Western Amhara, Ethiopia. Ethiopian Medical Journal. 2016;54(3):109-16. PubMed PMID: 29115777.

10. Bogale D, Markos D, Kaso M. Prevalence of female genital mutilation and its effect on women's health in Bale zone, Ethiopia: a cross-sectional study. BMC public health. 2014;14:1076. doi: 10.1186/1471-2458-14-1076.

11. Sood S, Ramaiya A. Combining Theory and Research to Validate a Social Norms Framework Addressing Female Genital Mutilation. Front Public Health. 2022;9:19. doi: 10.3389/fpubh.2021.747823. PubMed PMID: WOS:000745974100001.

12. Abebe S, Dessalegn M, Hailu Y, Makonnen M. Prevalence and Barriers to Ending Female Genital Cutting: The Case of Afar and Amhara Regions of Ethiopia. International Journal of Environmental Research and Public Health. 2020;17(21):16. doi: 10.3390/ijerph17217960. PubMed PMID: WOS:000588904300001.

13. Melese G, Tesfa M, Sharew Y, Mehare T. Knowledge, attitude, practice, and predictors of female genital mutilation in Degadamot district, Amhara regional state, Northwest Ethiopia, 2018. BMC Women’s Health. 2020;20(1):9. doi: 10.1186/s12905-020-01041-2. PubMed PMID: WOS:000563899100003.

14. Gudeta TA, Regassa TM, Gamtessa LC. Female genital mutilation: prevalence, associated factors and health consequences among reproductive age group women in Keffa Zone, Southwest, Ethiopia. Reproductive Health. 2022;19(1):9. doi: 10.1186/s12978-022-01364-3. PubMed PMID: WOS:000764993700001.

15. Mudege NN, Egondi T, Beguy D, Zulu EM. The determinants of female circumcision among adolescents from communities that practice female circumcision in two Nairobi informal settlements. Health Sociology Review. 2012;21(2):242-50. doi: 10.5172/hesr.2012.21.2.242. PubMed PMID: 2012-25816-010.

16. Ifeanyichukwu OA, Oluwaseyi A, Adetunji L. Female genital mutilation: Attitude and practices among women in Okada community, Edo state. Journal of Medicine and Biomedical Research. 2015;14(2):138-50.

17. Alo OA, Gbadebo B. Intergenerational attitude changes regarding female genital cutting in Nigeria. Journal of Women's Health. 2011;20(11):1655-61. doi: 10.1089/jwh.2010.2610. PubMed PMID: 2011-26507-006.

18. Adeyinka DA, Oladimeji O, Aimakhu C. Female genital cutting: Its perception and practice in Igbo-Ora community, Nigeria. International Journal of Child Health and Human Development. 2009;2(2):143-50. PubMed PMID: 2009-21498-008.

19. Johnson OE, Okon RD. Perception and practice of female genital cutting in a rural community in southern Nigeria. African Journal of Reproductive Health. 2012;16(4):132-9. PubMed PMID: 23444550.

20. Galukande M, Kamara J, Ndabwire V, Leistey E, Valla C, Luboga S. Eradicating female genital mutilation and cutting in Tanzania: an observational study. BMC Public Health. 2015;15:1147. PubMed PMID: 26584655.

21. Bjälkander O, Leigh B, Harman G, Bergström S, Almroth L. Female genital mutilation in Sierra Leone: who are the decision makers? African Journal of Reproductive Health. 2012;16(4):119-31.

22. Diabate I, Mesple-Somps S. Female genital mutilation and migration in Mali: do return migrants transfer social norms? J Popul Econ. 2019;32(4):1125-70. doi: 10.1007/s00148-019-00733-w. PubMed PMID: WOS:000477028000002.

23. Gele AA, Bo BP, Sundby J. Have we made progress in Somalia after 30 years of interventions? Attitudes toward female circumcision among people in the Hargeisa district. BMC Research Notes. 2013;6:122. PubMed PMID: 23537232.

24. Central Statistics Department Ministry of Planning and National Development Somaliland Government. The Somaliland Health and Demographic Survey 2020. 2020.

25. UNICEF and Somalia Ministry of Planning & International Cooperation. Northeast Zone Multiple Indicator Cluster Survey 2011, Final Report. Nairobi, Kenya: UNICEF and Ministry of Planning and International Cooperation, 2014.

26. Alosaimi AN, Essen B, Riitta L, Nwaru BI, Mouniri H. Factors associated with female genital cutting in Yemen and its policy implications. Midwifery. 2019;74:99-106. doi: 10.1016/j.midw.2019.03.010. PubMed PMID: WOS:000465986700013.

27. Abdulah DM, Sedo BA, Dawson A. Female Genital Mutilation in Rural Regions of Iraqi Kurdistan: A Cross-Sectional Study. Public Health Reports. 2019;134(5):514-21. doi: 10.1177/0033354919860512.

28. Ali H, Arafa AE, El Fattah Abd Allah Shehata NA, Fahim AS. Prevalence of Female Circumcision among Young Women in Beni-Suef, Egypt: A Cross-Sectional Study. Journal of Pediatric & Adolescent Gynecology. 2018;31(6):571-4. PubMed PMID: 30064003.

29. Mohammed ES, Seedhom AE, Mahfouz EM. Female genital mutilation: current awareness, believes and future intention in rural Egypt. Reproductive Health. 2018;15(1):175. PubMed PMID: 30333019.

30. Salama I, Sami S, Rabah T, Salama S, Mohsin AA. Female genital mutilation among egyptian school children. European Journal of Pediatrics. 2017;176(11):1463-4. doi: 10.1007/s00431-017-2979-8.

31. Zayed AA, Ali AA. Abusing female children by circumcision is continued in Egypt. Journal of Forensic & Legal Medicine. 2012;19(4):196-200. PubMed PMID: 22520370.

32. Milaat WA, Ibrahim NK, Albar HM. Reproductive health profile and circumcision of females in the Hali semi-urban region, Saudi Arabia: A community-based cross-sectional survey. Annals of Saudi Medicine. 2018;38(2):81-9. PubMed PMID: 29620540.
