# Supplementary material for: The global prevalence of female genital mutilation/cutting: A systematic review and meta-analysis of national, regional, facility, and school-based studies
Source: PLoS Med. 2022 Sep 1;19(9):e1004061. doi: 10.1371/journal.pmed.1004061 (PMC9436112; doi:10.1371/journal.pmed.1004061)
Supplement: S2 Appendix — Table A. Characteristics of school, community, or facility-based studies excluding studies on migrant populations. Table B. Studies reporting FGM/C in women and girls in school, community, or facility-based studies excluding studies on migrant populations. #Among respondents aware of FGM/C. ##Out of 342 women, 49 reported FGM/C in daughter(s) (14.3%).*Out of the female school teachers. **Without excluding those who were unsure if they had been mutilated. ***Prevalence according to clinical examination. Table C. Types of FGM/C in school, community, or facility-based studies excluding studies on migrant populations. *Clitoridectomy; **Manually calculated from the report; †Flesh removed (Type I or II); ¶Genital area was sewn after cutting; ‡Genital area was nicked (without cutting); #Clitoral nicking. Table D. Characteristics of FGM/C procedure in school, community, or facility-based studies excluding studies on migrant populations. (DOCX) [file pmed.1004061.s011.docx]

**S2 Appendix.** School, Community or Facility based studies excluding studies on migrant populations

**Table A.** Characteristics of school, community or facility-based studies excluding studies on migrant populations

|  | | | | | | | | | | | | |
| --- | --- | --- | --- | --- | --- | --- | --- | --- | --- | --- | --- | --- |
|  | **Author** | **Year** | **Data**  **Collection** | **Sampling method and Study Design** | **Sub-Region** | **Population Description** | **Ethnicity** | **Age (years)** | **Exam or Patient report** | **Data Collection Site** | **Types** | **Risk of Bias** |
| **AFR** | | | | | | | | | | | | |
| **Burkina Faso** | Ndiaye[1] | 2010 | 2007 | Purposive, Cross-Sectional | Fada Ngourma | Women who gave birth | NA | 14-44 | Exam | Hospital /Clinic  (Multiple) | 1,2,3 | Low |
|  | Ouédraogo [2] | 2017 | NA | Convenience, Cross Sectional | Boromo and Bittou | Hawkers (street vendors) | NA | 13-24 | Patient Report | Traffic Station | NA | Mod. |
| **Gambia** | Kaplan[3] | 2013 | 2010-2011 | Purposive, Cross Sectional | Western Health Region | Women examined for antenatal care or delivery in hospitals and health centers | Mandinka (n=175), Wolof (n=120), Fula (n=116), Sarahole (n=30), Djola (n=76), Serer (n=13), Other (n=19) | 12-45 | Exam | Hospital/Clinic | 1,2 | Mod. |
|  |  |  |  |  |  |  |  |  |  |  |  |  |
| **Tanzania** | Suleiman[4] | 2021 | 2004-2014 | All women in the hospital facility, Retrospective facility-based | Kilimanjaro region | Women who delivered singletons | NA | 15-49 | Exam | Hospital/Clinic | 1,2,3,4 | Low. |
| **Ethiopia** | Gudu[5] | 2017 | 2015 | Purposive, Cross-Sectional | Jijiga | Nulliparous women who gave birth | Somali (98.6%) and Oroma (1.4%) | Mean 21.8 | Patient report | Hospital s/Clinics | 2 and 3 | Mod. |
|  | Gebremariam[6] | 2016 | 2014 | Muti-stage stratified random sampling, Cross-Sectional | Jijiga district, Somali regional state, eastern Ethiopia | High school and college students | Somali (65%), Amhara (18.7%), Oromo (29%), Other (13.4%) | 15-24 | Patient report | School | 1,3 | Low |
|  | Tamire[7] | 2013 | 2011 | Multi-staged cluster sampling, Cross-Sectional | Hadiya zone, Southern Ethiopia | High school students | Hadiya (89.0%), Gurage (4.0%), Kambata (3.6%), Amhara (2.3%), Silte (0.9%), other (0.3%) | 13-25 | Patient report | School | NA | Low |
|  | Abathun[8] | 2018 | 2015 | Purposive (location), stratified random sampling (participants), Cross-Sectional | Harari and Somali Regions of Ethiopia | High school students | Somali, Harari, Others | 16-22 | Patient Report | School | 1 and 3 | Low |
|  | Shay[9] | 2010 | 2008 | Random sampling, Cross-Sectional | Addis Ababa | School girls | Amhara (31%), Ooromo (23.6%), Tigray (6.4%), Guraghe (31.2%), Others (7.9%) | <5-20+ | Patient Report | School  (Multiple) | NA | Mod. |
| **Ghana** | Nonterah [10] | 2019 | 2003-2013 | Retrospective audit of records, Cohort | Kassena-Nankana district- North Eastern Ghana | Attending Clinic | NA | <20-35+ | Both | Hospital/ Clinic | NA | Mod. |
|  | Sakeah[11] | 2018 | NA | Random and convenience, Cross-Sectional | Bawku municipality and Pusiga District | Women of reproductive age | Kusassi (1.33%) Maprussi (21.33%) Busanga (60.96%) Moshie (8.31%) Hausa (1.33%) Other (6.74%) | 15-49 | Patient Report | Household | NA | Low |
| **Mali** | Dicko-Traore[12] | 2014 | 2011 | Purposive, Cross Sectional | Bamako | Girls attending clinic | NA | 0-15 | Exam | Hospital/ Clinic | NA | Mod. |
| **Nigeria**  **Nigeria** | Ezeoke[13] | 2021 | NA | Multi-stage sampling of schools and purposive selection of students, cross sectional | North Central Nigeria | Secondary school students | NA | 13-19 | Patient Report | School | NA | Mod. |
|  | Chinawa [14] | 2020 | NA | Multi-stage sampling of schools; stratified selection of schools; random selection of students, cross sectional | Enugu metropolis | Secondary school students | Igbo (n=412, 99.5%), Hausa (n=1, 0.2%), Yoruba (n=1, 0.2%) | 13-21 | Patient Report | School | NA | Low. |
|  | Ezenyeaku[15] | 2011 | 2009-2010 | Purposive, Cross Sectional | Enugu and Awka (Southeast geopolitical zone of the Nigeria) | Attending clinic | NA | 14-50 | Patient report | Hospital/ Clinic | NA | Mod. |
|  | Lawani[16] | 2014 | 2012 | Purposive, Prospective | Abakaliki, Ebonyi, southeast Nigeria | Women seeking maternity services | NA | 15-49 | Exam | Hospital/ Clinic  (Multiple) | 1,2,3 | Mod. |
|  | Makinde[17] | 2012 | 2007 | Purposive, Descriptive prospective | South-West of Nigeria | Girls visiting emergency and gynecological wards | Hausa (4.1%), Ibo (4.1%), Yoruba (85.1%), Any Other (6.7%) | 0-14 | Exam | Hospital/ Clinic | 1,2,3,4 | Mod. |
|  | Dattijo[18] | 2010 | 2007 | Systematic random sampling, Cross-Sectional | Jos, north-central Nigeria | Attending antenatal care | Hausa (19.6%), Igbo (15.4%), Yoruba (13.8%), Berom (8.8%), Mughavul (5.8%), Ngas (5.4%) Edo (5.0%), Tarok (3.8%), Idoma (3.5%), Tiv (3.5%), other (15.4%). | <20-35+ | Patient Report | Hospital/ Clinic | Clitoridectomy, Excision, Infibulations | Mod. |
|  | Ogah[19] | 2019 | 2016 | Consecutive, Cross-Sectional | Ilorin, Kwara state | Attending health clinic | NA | 15-60 | Both | Hospital/ Clinic | NA | Mod. |
|  | Ashimi[20] | 2014 | 2014 | Systematic random sampling, Cross-Sectional | Jigawa state, Northwest Nigeria | Attending antenatal clinic | Hausa (70.6%), Fulani (26.0%), Yoruba (1.9%), Igbo (1.5%) | 15-40 | Patient report | Hospital/ Clinic  (Multiple) | 1,2, Hymen removal, Gishiri, Angurya | Low |
|  | Ashimi[21] | 2015 | 2014 | Systematic random sampling, Cross-Sectional | Three clinics, Birnin Kudu, Jigawa state | Infants presented to 3 clinics | Hausa (73%), Fulani (27%) | 1-21+ days | Both | Hospital/ Clinic  (multiple) | 1,2,3,4 | Low |
|  | Adeniran [22] | 2015 | 2014 | Multistage and purposive, Cross-Sectional | 18 schools in Ilorin, North Central Nigeria, all those eligible were invited | Secondary school teachers | NA | 20-60 | Patient report | School | NA | Mod. |
|  | Ibrahim[23] | 2013 | 2012 | All possible participants were included, Cross-Sectional | Bayelsa state, Niger-Delta of Nigeria | Nurses and doctors | Not FGM/C only: Ijaw (48.3%), Igbo (24.6%), Hausa (1.7%), Others (18.6%) no response (6.8%) | < 25-55 + | Patient report | Hospital/ Clinic  (Multiple) | NA | Mod. |
|  | Dike[24] | 2012 | NA | Purposive, Cross-Sectional | Afikpo, Ebonyi State, Southeastern Nigeria | Female students of Nursing and Midwifery | NA | 15-45 | Patient Report | School | NA | Low |
|  | Garba[25] | 2012 | 2011 | Purposive, Cross Sectional | Kano, Northern Nigeria | Infants | Hausa/Fulani ethnic group (58%), other ethnic group (42.3 %) | < 5 – 16 + days | Mother's report | Hospital/ Clinic | 1,2,3,4 | Low |
|  | Iliyasu[26] | 2012 | NA | Multistage sampling, Cross Sectional | Bayero University, Kano, Kano State, Northern Nigeria | University students | Hausa (44.3%), Fulani (17.5%), Yoruba (18.7%), Igbo (4.7%), Others (14.8%) | 17-40 | Patient Report | School | Flesh removed, nicked, sewn | Low |
|  | Jimoh[27] | 2018 | 2011 | All community invited, Cross-Sectional | Tsibiri village, a rural community in Giwa Local Government area of Kaduna state, north western region of Nigeria. | Women and girls who were married | NA | 15-49,10-14 | Patient Report | Community | NA | Low |
|  | Agbede[28] | 2019 | 2018 | Convenience, Cross Sectional | 4 wards in Ede South LGA, Osun State | Parents of daughters with FGM/C | Yoruba (94.7%), Igbo (3.7%), Hausa (1.6%) | 15- 40+ (parents) | Patient Report | Community | NA | Mod. |
| **Sierra Leone** | Bjälkander[29] | 2012 | 2006 | Random sampling, Cross-Sectional | Bo Town, Bo District, Southern region and Makeni Town, Bombali District, Northern region | Attending clinic | Temne (30.5%), Limba (26%), Mende (20.8%), Fulah (4.5%), Loko (7.2%), Susu (2.7%), Kono (3.2%), Korankoh (3.2%), Madingo (1.9%) | 11-45 | Patient Report | Hospital/ Clinic  (Multiple) | NA | Low |
| **Uganda** | Ivanova[30] | 2019 | 2018 | Convenience, Cross-Sectional | Nakivale refugee settlement | Refugees from DR Congo, Burundi, Rwanda, Ethiopia, Somalia, Uganda, South Sudan, Tanzania, Eritrea, Kenya | NA | 13-19 | Patient Report | Refugee Camp | NA | Mod. |
| **EMR** | | | | | | | | | | | | |

| **Djibouti** | Minsart[31] | 2015 | 2012-2014 | All possible participants, Cohort | Djibouti City | Women who gave birth | NA | <25-35+ | Patient report and exam | Hospital/ Clinic | 1,2,3 | Mod. |
| --- | --- | --- | --- | --- | --- | --- | --- | --- | --- | --- | --- | --- |
| **Somalia** | Adigüzel[32] | 2018 | 2017 | Purposive, Cross-Sectional | Mogadishu, Beladwayne, Kismaayo, Jawhar, Ceelbuur, Baardhere, Benadir, lower Shabelle, middle Shabelle, Hiiraan, Galgaduud, Mudug, Bakool, Gedo, lower Jubba | Presented to the obstetrics and gynecology outpatient clinics, married with at least one daughter | NA | Mean 28.76SD ±8.77 | Both | Hospital/ Clinic  (Multiple) | 1,2,3,4 | Low |
| **Sudan** | Birge[33] | 2017 | 2014-2015 | Convenience, Cross-Sectional | Darfur | Patients/working at hospital | NA | 17 - 65 | Both | Hospital/ Clinic | 1,2,3 | Mod. |
|  | Ali[34] | 2012 | 2012 | Random, Cross-Sectional | Eight schools in Kassala, Eastern Sudan | Students | Hadandawa (15.0%), Biniamir (40.4%), other tribes (44.5%) | 9-16 | Patient Report | School  (Multiple) | NA | Mod. |
|  | Sharfi[35] | 2013 | 2007-2012 | Purposive, Cross-Sectional | Khartoum | University students and out-clinic patients | NA | 20-62 | Both | Hospital/ Clinic and School | 3 | Mod. |
|  | Akbas[36] | 2019 | 2016 | All possible participants, Cross-Sectional | Nyala | University students | NA | Mean 19.5 SD ±1.95 | Patient Report | School | NA | Low |
|  | Mahgoub[37] | 2019 | 2018-2019 | Multi-stage sampling of schools, Quasi-experimental *(Cross-sectional selection)* | Karary Locality, Khartoum State, Sudan | Students | Jaalia tribe (31.2%) | 14-17 | Patient report | School | NA | Mod. |
|  | Birge[38] | 2021 | 2018-2019 | Cross sectional, purposive | Nyala, Darfur | Attending clinic | NA | 18+ | NA | Clinic | 1,2,3 | Mod. |
| **Iraq** | Yasin[39] | 2013 | 2007-2009 | Convenience, Cross-Sectional | Erbil, Kurdistan region | Attending clinic | NA | 15-49 | Both | Hospital/Clinic  (Multiple) | 1,2 | Mod. |
|  | Saleem[40] | 2013 | 2011 | Purposive, Cross-Sectional | Kurdistan region | Attending clinic | NA | 0.5-20 | Both | Hospital/Clinics  (Multiple) | 1,2,3,4 | Mod |
| **Egypt** | Hassanin[41] | 2012 | 2011 | Purposive, Cross-Sectional | Upper Egypt | Mothers lived in urban areas, completed secondary school, heard about the ban, with children in school | NA | 8-14 | Patient Report | Hospital/Clinic  (Multiple) | NA | High |
|  | Mitwaly[42] | 2017 | 2014-2015 | Purposive, Cross-Sectional | Luxor city ,Upper Egypt | Attending clinic | NA | 15-49 | Exam | Hospital/Clinic | 1,2 | Mod. |
|  | Arafa[43] | 2018 | 2016-2017 | Multistage random sampling, Cross-Sectional | Beni-Suef | University students | NA | Mean/SD 20.89 ± 1.68 | Patient Report | School | NA | Mod. |
|  | Mostafa[44] | 2017 | 2016 | All possible participants, Cross-Sectional | Beni-Suef | Overweight and obese premenopausal women | NA | 20-49 | Patient Report | Hospital/Clinic | NA | Mod. |
|  | Abdel-Aleem[45] | 2016 | 2011-2014 | Purposive, Cross-Sectional | Assiut and Sohag | Recently married | NA | 17-31 | Both | Hospital/Clinic | 1,2,3 | Low |
|  | Ahmed[46] | 2017 | 2015-2016 | Purposive, Cross-Sectional | Suez Canal | Students attending clinic | NA | 14-19 | Patient Report | Hospital/Clinic  (Multiple) | NA | Mod. |
|  | Rasheed[47] | 2011 | 2008-2010 | All possible participants, Cross-Sectional | Sohag and Qena | Attending clinic | NA | 5-25 | Patient Report | Hospital/Clinic | NA | High |
|  | Abolfotouh[48] | 2015 | 2012-2013 | Convenience, Cross-Sectional | Upper (Southern) and lower (Northern) Egypt | Medical students from 19 Egyptian universities, each in a different governorate | NA | 18+ | Patient Report | Online | NA | Low |
|  | Elbendary[49] | 2021 | 2018 | Random selection, Cross-sectional | Fayoum | Attending Clinic | NA | 18-45 | Exam | Clinic | 1,2,3,4 | Low |
|  | Galal[50] | 2022 | NA | Online, Cross-Sectional | Upper and lower Egypt | Medical Students | NA | Mean 21 years | Patient report | Online | NA | Mod. |
| **Iran** | Dehghankhalili[51] | 2015 | 2010-2013 | Purposive, Cross-Sectional | Minab, Dehbaz, Bandar-e-Lenge, Qeshm, Bandar-e-Khamir, and Bastak in Hormozgan, Southern Iran | Attending Clinic | NA | 14-38 | Exam | Clinic | 1,2,3,4, clitoris nicking | Mod. |
| **Oman** | Al Hinai [52] | 2014 | 2013 | Purposive survey, Cross-sectional | Muscat, Al-Baona, Al-Dakhiliya, Sharqiya  North, Sharqiya South, Al-Dhahira | General population attending public spaces | NA | 16-55+ | Self-report | Community | NA | High |

| **SEAR** |
| --- |

| **Malaysia** | Rashid [53] | 2019 | NA | Snowball, Cross-Sectional | Kedah and Penang, Northern region of Peninsular Malaysia | Villagers | NA | 18+ | Patient report | Village | 4 | Mod. |
| --- | --- | --- | --- | --- | --- | --- | --- | --- | --- | --- | --- | --- |
|  | Rashid[54] | 2009 | 2008-2009 | Convenience, cross sectional | Five villages in north Malaysia. | All women from villages | NA | 1 month-91 years | Patient report | Villages | NA | Low |
|  | Khalid [55] | 2017 | 2012 | Sequential convenience, Cross-Sectional | Selangor | Attending Clinic | Muslims alone: Malay (88.3%) Indonesian (5%), Others (6.7%) / Non- Muslims alone: Chinese (57.1%), Indians (32.7%), Others (10.2%) | 18+ | Patient report | Hospital/Clinic  (Multiple) | NA | Mod. |

Abbreviations: AFR: African Region; EMR: Eastern Mediterranean Region; SEAR: South East Asian Region

**Table B.** Studies reporting FGM/C in Women and Girls in School, Community or Facility based excluding studies on migrant populations.

|  | **Author** | **Year** | **Sampling method** | **Sub- Region** | | **Population description** | **Age** | **FGM/C (%)** | **Total FGM** | **Sample Size** |
| --- | --- | --- | --- | --- | --- | --- | --- | --- | --- | --- |
| **AFR** | | | | | | | | | | |
| **Nigeria** | Ashimi[20] | 2014 | Systematic random sampling | Jigawa state, Northwest Nigeria | | Attending antenatal clinic | 15-40 | 39.1%^#^ | 100 | 256 |
|  | Ashimi [21] | 2015 | Systematic random sampling | Birnin Kudu, Jigawa state | | Infants presented to 3 clinics | 1-21+ days | 47.8% | 215 | 450 |
|  | Ezenyeaku [15]^##^ | 2011 | Purposive | Enugu and Awka, Southeast Nigeria | | Attending clinic | 14-50 | 42.1% | 144 | 342 |
|  | Makinde[17] | 2012 | Purposive | South-West of Nigeria | | Visiting emergency and gynecological wards | 0-14 | 41.9% | 237 | 565 |
|  | Ogah[19] | 2019 | Consecutive | Ilorin, Kwara state | | Attending health clinic | 15-60 | 49% | 98 | 200 |
|  | Adeniran [22] | 2015 | Multistage and purposive | 18 schools in Ilorin, North Central Nigeria | | Secondary school teachers | 20-60 | 42.2%* | 109 | 258 |
|  | Ibrahim[23] | 2013 | All possible participants were included | Bayelsa state, Niger-Delta of Nigeria | | Nurses and doctors | <25 - 55 + (not only FGM/C sample) | 27.1% | 19 | 70 |
|  | Dike[24] | 2012 | Purposive | Afikpo, Ebonyi State, Southeastern Nigeria | | Nursing and midwifery students | 15-45 | 54.3% | 146 | 269 |
|  | Iliyasu[26] | 2012 | Multistage random sampling | Bayero University, Kano, Kano State, Northern Nigeria | | University Students | 17-40 | 12.1% | 43 | 359 |
|  | Lawani[16] | 2014 | Purposive | Abakaliki, Ebonyi, southeast Nigeria | | Women seeking maternity services at 2 clinics | 15-49 | 66.3% | 342 | 516 |
|  | Garba[25] | 2012 | Purposive | Kano, Northern Nigeria | | Infants attending clinic | < 5 –16+ days | 13% | 26 | 200 |
|  | Agbede[28] | 2019 | Convenience | Four wards Ede South LGA, Osun State | | Parents of daughters with FGM/C | 15-40+ (parents) | 38.8% | 146 | 376 |
|  | Dattijo[18] | 2010 | Systematic random sampling | Jos, north-central Nigeria | | Attending antenatal care | < 20 –35+ | 31.3% | 81 | 260 |
|  | Ezeoke[13] | 2021 | Multi-stage sampling of schools and purposive selection of students | North Central Nigeria | | Secondary school students | 13-19 | 35%** | 699 | 2000 |
|  | Chinawa[14] | 2020 | Multi-stage sampling of schools; stratified selection of schools; random selection of students | Enugu metropolis | | Secondary school students | 13-21 years | 9.4% | 39 | 414 |
|  | Jimoh[27] | 2018 | All community invited. | Tsibiri village, a rural community in Giwa Local Government area of Kaduna state, northwestern region of Nigeria. | | Women of reproductive age | 15-49,10-14 (married) | 0.4% | 1 | 220 |
| **Mali** | Dicko-Traore  [12] | 2014 | Purposive | Bamako | | Girls who were hospitalized | 0-15 | 73% | 224 | 305 |
| **Sierra Leone** | Bjälkander [29] | 2012 | Random sampling | Bo Town, Bo District, in the southern region and in Makeni Town, Bombali District, in the northern region of Sierra Leone | | Attending antenatal care clinic (Facility-based) | 11-45 | 100% | 258 | 258 |
| **Ethiopia** | Tamire[7] | 2013 | Multi-staged cluster sampling | Hadiya zone, Southern Ethiopia | | High school students | 13 - 25 | 82.2% | 641 | 780 |
|  | Abathun[8] | 2018 | Purposive (location), stratified (participants) | Somali and Harari Regions | | Sampled girls and boys who attended primary and secondary schools from the selected schools | 16-22 | 32.1% | 79 | 246 |
|  | Gebremariam[6] | 2016 | Muti-stage stratified random sampling | Jijiga district, Somali regional state, eastern Ethiopia | | High school and college female students | 15-24 | 82.6% | 538 | 651 |
|  | Gudu[5] | 2017 | Purposive | Jijiga | | Nulliparous women who gave birth | Mean 21.8 | 91.7% | 264 | 288 |
|  | Shay[9] | 2010 | Random sampling | Addis Ababa | | School girls | < 5 – 20+ | 26% | 106 | 407 |
| **Ghana** | Nonterah[10] | 2019 | Retrospective audit of records | Kassena-Nankana district- North Eastern Ghana | | Women who had delivered children at a hospital. | <20-35+ | 17.7% | 1647 | 9306 |
|  | Sakeah[11] | 2018 | Random and convenience | Bawku municipality and Pusiga District | | Women of reproductive age | 15-49 | 61.3% | 509 | 830 |
| **Burkina Faso** | Ndiaye[1] | 2010 | Purposive | Fada Ngourma | | Women who gave birth in 4 maternity wards | 14-44 | 59% | 210 | 354 |
|  | Ouédraogo[2] | 2017 | Convenience | Boromo and Bittou | | Hawkers/Street vendors | 13-24 | 60.6% | 160 | 264 |
| **Uganda** | Ivanova[30] | 2019 | Convenience | Nakivale refugee settlement | | Refugees | 13-19 | 10.4% | 27 | 260 |
| **Gambia** | Kaplan[3] | 2013 | Purposive | Western Health Region | | Women examined for antenatal care or delivery in hospitals and health centers | 12-45 | 75.6% | 431 | 570 |
| **Tanzania** | Suleiman[4] | 2021 | All possible participants, hospital records | Kilimanjaro region | | Women who delivered single births | 15-49 | 15.4% | 4675 | 30,286 |
| **SEAR** | | | | | | | | | | |
| **Malaysia** | Rashid[53] | 2019 | Snowball | Kedah and Penang, in the Northern region of Peninsular Malaysia | | Villagers | 18+ | 99.3% | 601 | 605 |
|  | Rashid[54] | 2009 | Convenience, cross sectional | Five villages in north Malaysia. | | All women from villages | 1 month-91 years | 94.8% | 597 | 630 |
|  | Khalid[55] | 2017 | Sequential convenience | Selangor | | Attending clinic | 18+ | 70.6% | 353 | 500 |
| **EMR** | | | | | | | | | | |
| **Djibouti** | Minsart [31] | 2014 | All possible participants | | Djibouti City | Women who gave birth | <25-35+ | 95.5% | 614 | 643 |
| **Somalia** | Adigüzel[32] | 2018 | Purposive | | Mogadishu, Beladwayne, Kismaayo, Jawhar, Ceelbuur, and Baardhere, and regions such as Benadir, lower Shabelle, middle Shabelle, Hiiraan, Galgaduud, Mudug, Bakool, Gedo, and lower Jubba | Presented to the obstetrics and gynecology outpatient clinics, married with at least one daughter (Facility based) | Mean 28.76±8.77 | 99.7% | 355 | 356 |
| **Sudan** | Mahgoub[37] | 2019 | Multi-stage sampling of schools | | Karary Locality, Khartoum State, Sudan | Students | 14-17 | 30.3% | 47 | 154 |
|  | Birge[38] | 2021 | Cross sectional, Purposive | | Nyala, Darfur | Attending clinic | 18+ | 87.2 | 3767 | 4320 |
|  | Birge[33] | 2017 | Convenience | | Darfur | Patients/working at hospital | 17 - 65 | 87.9% | 210 | 239 |
|  | Akbas[36] | 2019 | All possible participants | | Nyala | University students | Mean/SD 19.5±1.95 | 80.1% | 330 | 412 |
|  | Sharfi[35] | 2013 | Purposive | | Khartoum | University students and out-clinic patients | 20-62 | 73.4% | 1468 | 2000 |
|  | Ali[34] | 2012 | Random | | Eight schools in Kassala, Eastern Sudan | Students | 9-16 | 83.3% | 810 | 972 |
| **Iran** | Dehghankhalili[51] | 2015 | Purposive | | Minab, Dehbaz, Bandar-e-Lenge, Qeshm, Bandar-e-Khamir, and Bastak (rural areas) in Hormozgan, Southern Iran | Attending healthcare clinic | 14-38 | 68.5% | 535 | 780 |
| **Egypt** | Rasheed[47] | 2011 | All possible participants | | Sohag and Qena, Egypt | Attending clinic | 5-25 | 89.2% | 3711 | 4158 |
|  | Mitwaly[42] | 2017 | Purposive | | Luxor city, Upper Egypt | Attending clinic | 15-49 | 89.1% | 1047 | 1175 |
|  | Arafa[43] | 2018 | Multistage random sampling | | Beni-Suef | University students | Mean 20.89 (SD ± 1.68) | 47.3% | 815 | 1723 |
|  | Ahmed[46] | 2017 | Purposive | | Suez Canal | Students attending clinic | 14-19 | 66.2% | 135 | 204 |
|  | Mostafa[44] | 2017 | All possible participants | | Beni-Suef | Overweight and obese premenopausal women attending nutrition clinic | 20-49 | 59.3% | 89 | 150 |
|  | Abdel-Aleem[45] | 2016 | Purposive | | Assiut and Sohag | Recently married attending 2 clinics | 17-31 | 87.4% | 376 | 430 |
|  | Hassanin[41] | 2012 | Purposive | | Upper Egypt | Mothers in urban areas, completed secondary school, heard about the ban, with children in school | 8-14 | 71.6% | 358 | 500 |
|  | Abolfotouh[48] | 2015 | Convenience | | N/A | Medical students | 18+ | 14.7% | 47 | 320 |
|  | Elbendary[49] | 2021 | Random selection | | Fayoum | Attending clinic | 18-45 | 62% | 62 | 100 |
|  | Galal[50] | 2022 | All possible participants | | Upper and lower Egypt | Medical Students | Mean 21 years | 19.4% | 142 | 733 |
| **Iraq** | Yasin[39] | 2013 | Convenience | | Erbil, Kurdistan region, Iraq | Attending clinic | 15-49 | 58.6%*** | 1164 | 1987 |
|  | Saleem[40] | 2013 | Purposive | | Kurdistan region | Attending clinics | 0.5-20 | 23% | 348 | 1508 |
| **Oman** | Al Hinai [52] | 2014 | Purposive | | Muscat, Al-Baona, Al-Dakhiliya, Sharqiya  North, Sharqiya South, Al-Dhahira | General population attending public spaces | 16-55+ | 78% | 78 | 100 |

^#^Among respondents aware of FGM/C. ^##^Out of 342 women, 49 reported FGM/C in daughter(s) (14.3%). *Out of the female school teachers. **Without excluding those who were unsure if they had been mutilated. *** Prevalence according to clinical examination.

Abbreviations: AFR: African Region EMR: Eastern Mediterranean Region SEAR: South East Asian Region FGM/C: Female Genital Mutilation/Cutting. FGM/C:

**Table C.** Types of FGM/C in School, Community or Facility based studies excluding studies on migrant populations.

| **Country** | **Author** | **Year** | **FGM/C Sample** | **Sample Size** | | **Type 1 (%)** | **Type 2 (%)** | **Type 1 or 2(%)** | **Type 3 (%)** | **Type 4 (%)** | **Other Type / Did Not Know (%)** |
| --- | --- | --- | --- | --- | --- | --- | --- | --- | --- | --- | --- |
| **AFR** | | | | | | | | | | | |
| **Nigeria** | Ashimi[20] | 2014 | 100 | 323 |  | |  | Type I and type II together:2.0% |  |  | Gishiri cut (56.0%), Angurya (49.0%), type I and type II together (2.0%), one participant: removal of hymen. |
|  | Ashimi[21] | 2015 | 215 | 450 | 11.2% | | 2.3% |  | 2.3% | 43.3% | Do not know (40.9%) |
|  | Dattijo[18] | 2010 | 81 | 260 | 33.2%* | |  |  |  |  | Excision (0.8%), Don't know (66%) |
|  | Makinde[17] | 2012 | 237 | 565 | 35.4% | | 58.2% |  | 6.3% |  |  |
|  | Lawani[16] | 2014 | 342 | 516 | 28.1% | | 59.6% |  | 12.3% |  |  |
|  | Garba[25] | 2012 | 26 | 200 | 96.2% | | 3.8% |  |  |  |  |
|  | Iliyasu[26] | 2012 | 43 | 359 |  | |  | 37.2%^†^ | 4.7%^¶^ | 9.3%^‡^ | Do not know (20.9%) |
| **Tanzania** | Suleiman[4] | 2021 | 4675 | 30,286 | 61% | | 37% |  |  |  | Type III and IV reported together 2% |
| **Gambia** | Kaplan[3] | 2013 | 431 | 570 | 75.6% | | 24.4% |  |  |  |  |
| **Burkina Faso** | Ndiaye[1] | 2010 | 210 | 354 | 47.1%** | | 46.7%** |  | 6.2%** |  |  |
| **Ethiopia** | Gebremariam[6] | 2016 | 538 | 651 | 49.3% | |  |  | 45.4% |  | Both type I and III (5.3%) |
|  | Gudu[5] | 2017 | 264 | 288 |  | | 7.6% |  | 92.4% |  |  |
| **SEAR** | | | | | | | | | | | |
| **Malaysia** | Rashid[53] | 2019 | 601 | 605 | |  |  |  |  | 100% |  |
| **EMR** | | | | | | | | | | | |
| **Egypt** | Mitwaly[42] | 2017 | 1047 | 1175 | | 58.5% | 41.5% |  |  |  |  |
|  | Abdel-Aleem[45] | 2016 | 376 | 430 | | 12.8% |  |  |  |  | Both type II and III (87.2%) |
|  | Elbendary[49] | 2021 | 62 | 100 | | 14% | 86% |  |  |  |  |
| **Iraq** | Yasin[39] | 2013 | 1164 (verified by clinical examination) | 1987 | | 99.6% | 0.4% |  |  |  |  |
|  | Saleem[40] | 2013 | 348 (with 239 examined cases) | 1508 | | 76.2% | 13.4% |  | 0% | 10.5% |  |
| **Iran** | Dehghankhalili[51] | 2015 | 535 | 780 | | 27.9% | 5.9% |  | 6% | 28.7%^#^ |  |
| **Somalia** | Adigüzel[32] | 2018 | 355 | 356 | | 82.8% | 10.4% |  | 2.3% | 4.5% |  |
| **Djibouti** | Minsart [31] | 2014 | 614 | 643 | | 1.1% | 60.1% |  | 38.8% |  |  |
| **Sudan** | Birge[38] | 2021 | 3767 | 4320 | | 9.1% | 37.3% |  | 53.6% |  |  |
|  | Birge[33] | 2017 | 210 | 239 | | 23.3% | 51% |  | 25.7% |  |  |
|  | Sharfi[35] | 2013 | 1468 | 2000 | |  |  |  | 62.9% |  |  |

^*^ Clitoridectomy. ^**^Manually calculated from the report. ^†^ flesh removed (Type I or II).  ^¶^ genital area was sewn after cutting. ^‡^ genital area was nicked (without cutting).  ^#^ Clitoral nicking.

Abbreviations: AFR: African Region; EMR: Eastern Mediterranean Region; SEAR: South East Asian Region; FGM/C: Female Genital Mutilation/Cutting

**Table D.** Characteristics of FGM/C Procedure in School, Community or Facility based studies excluding studies on migrant populations.

|  | **Author** | **Year** | **Age at FGM/C** | **Performer of FGM** | **Location of Procedure** |
| --- | --- | --- | --- | --- | --- |
| **AFR** | | | | | |
| **Ethiopia** | Gudu[5] | 2017 | Most at 7 years (40.3%) (Range: 2 – 9 years). |  |  |
|  | Gebremariam[6] | 2016 | Years: ≤ 6 (20.4%), 7-10 (49.8%), 11-14 (29.8%) | Traditional circumcisers (72.9%), traditional birth attendant (25.5%), health professional (1.6%) | Home (63.6%) |
|  | Tamire[7] | 2013 | Mean 11 (SD±2.3) years. | Traditional (69.1%), Medically Trained (29%), Don't Know (1.9%) | Home (82.5%) |
|  | Abathun[8] | 2018 | 6 to 14 years (45.8%), Don't know (23.8%) | Traditional (73%), Medically Trained (3.6%) |  |
|  | Shay[9] | 2010 | Years: <1 (20.8%), 1-5 (50.9%), 6-10 (18.9%), >10 (9.4%) | Traditional (77.4%), Medically trained (22.6%) |  |
| **Nigeria** | Ezeoke[13] | 2021 | Mean age: 3.85±3.24 (Range: 1 - 8) years |  |  |
|  | Chinawa[14] | 2020 | <5 years (51.3%), 5-15 years (15.4%), ≥16 years (33.3%) | Nurse/midwife (48.7%), Doctors (15.4%), Traditional expert (15.4%) Mother (12.8%), Grandmother (7.7%) |  |
|  | Makinde[17] | 2012 | < 1 month (41.7%), 1 month-1 year (51.5%), 1-10 years (3.4%), more than 10 years (3.4%) | Traditional (64.6%), Medically trained (35.4%) |  |
|  | Dattijo[18] | 2010 | Years: <1 (44.6%), 1-5 (41.9%) >5 (13.5%) | Traditional (56.5%), Medically trained (8.2%), Don't know (35.3%) |  |
|  | Ashimi[20] | 2014 |  | Traditional (100%) |  |
|  | Ashimi[21] | 2015 | Days: 1–7 (93.5%), 8–14 (4.4%), 15–21 (0.4%), 22–28 (0.9%), 29–35 (0%), 36–42 (0%), ≥43 (0.9%) | Traditional (100%) |  |
|  | Adeniran[22] | 2015 | Mean 4.76 (SD± 4.86) | Traditional (67.9%), Medically Trained (32.1%) | Home (63.9%), Clinic (36.1%) |
|  | Garba [25] | 2012 |  | Traditional (84.6%), Medically Trained (15.4%) |  |
|  | Iliyasu[26] | 2012 | 1 – 4 years (48.8%), 5 – 10 years (23.3%), infancy (4.7%), Don't know (18.6%) | Traditional (74.4%), Medically Trained (11.6%), Don't know (13.9%). | Home (55.8%), Clinic (4.7%), Circumciser's house (32.6%) |
|  | Agbede[28] | 2019 | Youngest child circumcised at age: 0–11 months (20.7%), 1–4 years (10.6%), 5–8 years (4.8%), 9–12 years (2.7%) |  |  |
| **Sierra Leone** | Bjälkander[29] | 2012 | Years: 0-1 (0.7%), 2-4 (7.1%), 5-9 (18.2%), 10-14 (41.6%), 15+ (25.3%), Don't know (7.1%) |  |  |
| **DR Congo** | Ivanova[30] | 2019 | Mean age: 7.3 (95% CI: 5.4, 9.1) years |  |  |
| **EMR** | | | | | |
| **Iraq** | Yasin[39] | 2013 | Years: <4 (16.6%), 4–7 (60.2%), 8–11 (21.2%), 12–15 (1.9%), ≥16 (0.1%) | Traditional (99.1%), Medically trained (0.9%) |  |
|  | Saleem[40] | 2013 | Years: 0–2 (20.9%), 3–4 (32.6%), 5–6 (25%), ≥7 years (20.9%) | Traditional (84.6%), Medically (15.4%) | City (25.9%), District (25.6%), Sub-District (32.9%), Village (15.7%) |
| **Egypt** | Hassanin[41] | 2012 | Years: ≤8 (25.7%), 8–9 (32.9%), 9–10 (19.3%), ≥11 (22.1%). | Medically (54.5%), No answer (54.1%) |  |
|  | Elbendary[49] | 2021 | Median age: 13.2 ±2.2 years | Gynecological doctor (32.5%), Surgeon (11.5%), Midwife (56%), Barber (0%) | Hospital (22.5%), Home (60%), private (17.5%) |
|  | Mitwaly[42] | 2017 | 6 years (most frequent) followed by 5 years | Traditional (4.4%), Medically (95.7%) |  |
|  | Ahmed[46] | 2017 | Mean/SD 7.8 ± 1.1 years | Traditional (32.6%) Medically (67.4%) |  |
|  | Rasheed[47] | 2011 | Mean/SD 8.2± 0.9 years | *Graph is unclear.* General Practitioners were the most common. | Home (74.3%), Clinic (25.7%) |
| **Somalia** | Adigüzel[32] | 2018 | 1-14 years | Traditional (74.4%), Medically Trained (24.2%), Don't know (1.4%). |  |
| **Sudan** | Sharfi[35] | 2013 | <6 years (96.9%) | Traditional (94.5%) |  |
|  | Akbas[36] | 2019 | 5-12 years (93%); mean age 7.97 ± 2.49 |  |  |
| **Iran** | Dehghankhalili[51] | 2015 | Mean 5.6 (SD± 3.6) (Ranging from 2 to 38 years) | Traditional (100%) |  |
| **Oman** | Al Hinai [52] | 2014 | Years: <1 (14.1%), 1-5 (26.9%), 6-10 (15.4), Don't Know (43.6%) | Traditional (85.9%), Medically (4.7%), Don't know (9.4%) | At home (85.9%), Clinic (4.7%), Don't know (9.4%) |
| **SEAR** | | | | | |
| **Malaysia** | Rashid[53] | 2019 | Median: 6 years; Range: birth to 30 years | Traditional (60.7%), Medically (39.3%) |  |
|  | Rashid [54] | 2009 | < 12 months old (88.6%), 13 - 24 months (6%), 24 months + (6%) | Traditional (67.8%), Medical (28.4%), Don't know (3.8%) | Home (67.8%), Clinic (28.4%), unsure (3.8%) |

Abbreviations: AFR: African Region; EMR: Eastern Mediterranean Region; SEAR: South East Asian Region; FGM/C: Female Genital Mutilation/Cutting

**References**

1. Ndlaye P, Diongue M, Faye A, Ouedraogo D, Dia AT. Female genital mutilation and complications in childbirth in the province of Gourma (Burkina Faso). Sante Publique. 2010;22(5):563-70.

2. Ouedraogo SY, Sisawo EJ, Huang SL. Sexual abuse and risky sexual behaviors among young female hawkers in Burkina Faso: a mixed method study. BMC International Health & Human Rights. 2017;17(1):1. PubMed PMID: 28052765.

3. Kaplan A, Forbes M, Bonhoure I, Utzet M, Martin M, Manneh M, et al. Female genital mutilation/cutting in The Gambia: long-term health consequences and complications during delivery and for the newborn. International Journal of Women's Health. 2013;5:323-31. PubMed PMID: 23843705.

4. Suleiman IR, Maro E, Shayo BC, Alloyce JP, Masenga G, Mahande MJ, et al. Trend in female genital mutilation and its associated adverse birth outcomes: A 10-year retrospective birth registry study in Northern Tanzania. Plos One. 2021;16(1):13. doi: 10.1371/journal.pone.0244888. PubMed PMID: WOS:000607915500017.

5. Gudu W, Abdulahi M. Labor, Delivery and Postpartum Complications in Nulliparous Women with Female Genital Mutilation Admitted to Karamara Hospital. Ethiopian Medical Journal. 2017;55(1):11-7. PubMed PMID: 29148634.

6. Gebremariam K, Assefa D, Weldegebreal F. Prevalence and associated factors of female genital cutting among young adult females in Jigjiga district, eastern Ethiopia: A cross-sectional mixed study. International Journal of Women's Health. 2016;8:357-65. doi: 10.2147/IJWH.S111091.

7. Tamire M, Molla M. Prevalence and belief in the continuation of female genital cutting among high school girls: a cross - sectional study in Hadiya zone, Southern Ethiopia. BMC Public Health. 2013;13:1120. PubMed PMID: 24304497.

8. Abathun AD, Sundby J, Gele AA. Pupil's perspectives on female genital cutting abandonment in Harari and Somali regions of Ethiopia. BMC Women's Health. 2018;18(1):167. PubMed PMID: 30333023.

9. Shay TZ, Haidar J, Kogi-Makau W. Magnitude of and driving factors for female genital cutting in schoolgirls in Addis Ababa, Ethiopia: A cross-sectional study. SAJCH South African Journal of Child Health. 2010;4(3):78-82.

10. Nonterah EA, Kanmiki EW, Agorinya IA, Sakeah E, Tamimu M, Kagura J, et al. Prevalence and adverse obstetric outcomes of female genital mutilation among women in rural Northern Ghana. European Journal of Public Health. 2019;21:21. PubMed PMID: 31637426.

11. Sakeah E, Debpuur C, Oduro AR, Welaga P, Aborigo R, Sakeah JK, et al. Prevalence and factors associated with female genital mutilation among women of reproductive age in the Bawku municipality and Pusiga District of northern Ghana. BMC Women's Health. 2018;18(1):150. PubMed PMID: 30227845.

12. Dicko-Traore F, Diakite FL, Diakite AA, Konate D, Keita JT, Traore F, et al. Connaisssances, attitudes et pratiques des meres relatives a l'excision a Bamako [Mothers knowledges, attitudes and practices on female genital excision in Bamako]. Mali Medical. 2014;29(1):34-9. PubMed PMID: 30049139.

13. Ezeoke GG, Adeniran AS, Adesina KT, Fawole AA, Ijaiya MA, Olarinoye AO. Female adolescents and the future of female genital mutilation/cutting: a report from an endemic area. African Health Sciences. 2021;21(4):1808-16. doi: 10.4314/ahs.v21i4.38. PubMed PMID: WOS:000754471200038.

14. Chinawa AT, Chinawa JM, Ossai EN, Aronu AE, Ozokoli GE, Enebe J. Pattern of Female Genital Mutilation among Adolescents Attending Secondary Schools' in Enugu Metropolis. Journal of Tropical Pediatrics. 2021;67(1):10. doi: 10.1093/tropej/fmaa103. PubMed PMID: WOS:000637542600031.

15. Ezenyeaku C, Okeke T, Chigbu C, Ikeako L. Survey of Women's Opinions on Female Genital Mutilation (FGM) in Southeast Nigeria: Study of Patients Attending Antenatal Clinic. Annals of Medical & Health Sciences Research. 2011;1(1):15-20. PubMed PMID: 23209950.

16. Lawani LO, Onyebuchi AK, Iyoke CA, Okeke NE. Female genital mutilation and efforts to achieve Millennium Development Goals 3, 4, and 5 in southeast Nigeria. International Journal of Gynaecology & Obstetrics. 2014;125(2):125-8. PubMed PMID: 24602774.

17. Makinde ON, Elusiyan JB, Adeyemi AB, Taiwo OT. Female Genital Mutilation: Are We Winning? East African Medical Journal. 2012;89(6):193-8. PubMed PMID: 26856041.

18. Dattijo LM, Nyango DD, Osagie OE. Awareness, perception and practice of female genital mutilation among expectant mothers in Jos University Teaching Hospital Jos, north-central Nigeria. Nigerian Journal of Medicine: Journal of the National Association of Resident Doctors of Nigeria. 2010;19(3):311-5. PubMed PMID: 20845637.

19. Ogah J, Kolawole O, Awelimobor D. High risk human papilloma virus (HPV) common among a cohort of women with female genital mutilation. African Health Sciences. 2019;19(4):2985-92. doi: 10.4314/ahs.v19i4.19.

20. Ashimi AO, Amole TG. Perception and attitude of pregnant women in a rural community north-west Nigeria to female genital mutilation. Archives of Gynecology and Obstetrics. 2015;291(3):695-700. doi: 10.1007/s00404-014-3478-z.

21. Ashimi AO, Amole TG, Iliyasu Z. Prevalence and predictors of female genital mutilation among infants in a semi urban community in northern Nigeria. Sexual and Reproductive Healthcare. 2015;6(4):243-8. doi: 10.1016/j.srhc.2015.05.005.

22. Adeniran AS, Fawole AA, Balogun OR, Ijaiya MA, Adesina KT, Adeniran IP. Female genital mutilation/cutting: Knowledge, practice and experiences of secondary schoolteachers in North Central Nigeria. South African Journal of Obstetrics and Gynaecology. 2015;21(2):39-43. doi: 10.7196/SAJOG.1047.

23. Ibrahim IA, Oyeyemi AS, Ekine A. Knowledge, attitude and practice of female genital mutilation among health care workers in the Niger delta. International Journal of Gynecology and Obstetrics. 2015;131:E257-E8.

24. Dike EI, Ojiyi EC, Chukwulebe AE, Egwuatu VF. Female genital mutilation: Awareness and attitude of nursing and midwifery students in Afikpo, Nigeria. Internet Journal of Gynecology and Obstetrics. 2012;16(3). doi: 10.5580/2c1b.

25. Garba ID, Muhammed Z, Abubakar IS, Yakasai IA. Prevalence of female genital mutilation among female infants in Kano, Northern Nigeria. Archives of Gynecology & Obstetrics. 2012;286(2):423-8. PubMed PMID: 22491808.

26. Iliyasu Z, Abubakar IS, Galadanci HS, Haruna F, Aliyu MH. Predictors of female genital cutting among university students in northern Nigeria. Journal of Obstetrics & Gynaecology. 2012;32(4):387-92. PubMed PMID: 22519488.

27. Jimoh AO, Adaji SE, Adelaiye H, Olorukooba AA, Bawa U, Ibrahim HI, et al. A cross-sectional study of traditional practices affecting maternal and newborn health in rural Nigeria. The Pan African Medical Journal. 2018;31:64. PubMed PMID: 31007811.

28. Agbede C, Kio J, Adeyemo O. Correlates of intention to discontinue female genital mutilation in Osun State, Nigeria: Implication for women well-being. Public Health Nursing. 2019;36(5):615-22. doi: 10.1111/phn.12633. PubMed PMID: WOS:000487345900005.

29. Bjälkander O, Bangura L, Leigh B, Berggren V, Bergström S, Almroth L. Health complications of female genital mutilation in Sierra Leone. International Journal of Women's Health. 2012;4(1):321-31. doi: 10.2147/ijwh.s32670.

30. Ivanova O, Rai M, Mlahagwa W, Tumuhairwe J, Bakuli A, Nyakato VN, et al. A cross-sectional mixed-methods study of sexual and reproductive health knowledge, experiences and access to services among refugee adolescent girls in the Nakivale refugee settlement, Uganda. Reproductive Health. 2019;16(1):35. PubMed PMID: 30890170.

31. Minsart AF, N'Guyen T S, Ali Hadji R, Caillet M. Maternal infibulation and obstetrical outcome in Djibouti. Journal of Maternal-Fetal & Neonatal Medicine. 2015;28(14):1741-6. PubMed PMID: 25234101.

32. Adigüzel C, Baş Y, Erhan MD, Gelle MA. The Female Genital Mutilation/Cutting Experience in Somali Women: Their Wishes, Knowledge and Attitude. Gynecologic and Obstetric Investigation. 2019;84(2):118-27. doi: 10.1159/000493130.

33. Birge O, Arslan D, Ozbey EG, Adiyeke M, Kayar I, Erkan MM, et al. Which type of circumcision is more harmful to female sexual functions? Clinical and Experimental Obstetrics and Gynecology. 2017;44(5):691-4. doi: 10.12891/ceog3464.2017.

34. Ali AA, Okud A, Mohammed AA, Abdelhadi MA. Prevalence of and factors affecting female genital mutilation among schoolgirls in Eastern Sudan. International Journal of Gynaecology & Obstetrics. 2013;120(3):288-9. PubMed PMID: 23219094.

35. Sharfi AR, Elmegboul MA, Abdella AA. The continuing challenge of female genital mutilation in Sudan. African Journal of Urology. 2013;19(3):136-40. doi: 10.1016/j.afju.2013.06.002.

36. Akbas M, Birge O, Arslan D, Ozbey EG. Opinions of university students about female genital mutilation in sudan. Eastern Mediterranean Health Journal. 2019;25(4):225-9. doi: 10.26719/emhj.18.033.

37. Mahgoub E, Nimir M, Abdalla S, Elhuda DA. Effects of school-based health education on attitudes of female students towards female genital mutilation in Sudan. Eastern Mediterranean Health Journal. 2019;25(6):406-12. doi: 10.26719/emhj.18.053.

38. Birge O, Serin AN, Bakir MS. Female genital mutilation/cutting in sudan and subsequent pelvic floor dysfunction. BMC Women’s Health. 2021;21(1):8. doi: 10.1186/s12905-021-01576-y. PubMed PMID: WOS:000734775000006.

39. Yasin BA, Al-Tawil NG, Shabila NP, Al-Hadithi TS. Female genital mutilation among Iraqi Kurdish women: a cross-sectional study from Erbil city. BMC public health. 2013;13:809.

40. Saleem RA, Othman N, Fattah FH, Hazim L, Adnan B. Female genital mutilation in Iraqi Kurdistan: description and associated factors. Women & Health. 2013;53(6):537-51. PubMed PMID: 23937728.

41. Hassanin IM, Shaaban OM. Impact of the complete ban on female genital cutting on the attitude of educated women from Upper Egypt toward the practice. International Journal of Gynaecology & Obstetrics. 2013;120(3):275-8. PubMed PMID: 23245920.

42. Mitwaly A AB, Abd El Aal DEM, Aziz PA, Hassanin AI, Abbas AM. A recent look for the implication and attitude of practicing female genital mutilation in upper Egypt: a cross sectional study. International Journal of Reproduction, Contraception, Obstetrics and Gynecology 2017;6(10):4224-4226 <http://dxdoiorg/1018203/2320-1770ijrcog20174398>. 2017.

43. Arafa AE, Elbahrawe RS, Shawky SM, Abbas AM. Epidemiological and gynecological correlates with female genital mutilation among Beni-Suef University students; cross sectional study. Middle East Fertility Society Journal. 2018;23(3):184-8. doi: 10.1016/j.mefs.2017.11.005.

44. Mostafa AM, Khamis Y, Helmy HK, Arafa AE, Abbas AM. Prevalence and patterns of female sexual dysfunction among overweight and obese premenopausal women in Upper Egypt; a cross sectional study. Middle East Fertility Society Journal. 2018;23(1):19-22. doi: 10.1016/j.mefs.2017.08.006.

45. Abdel-Aleem MA, Elkady MM, Hilmy YA. The relationship between female genital cutting and sexual problems experienced in the first two months of marriage. International Journal of Gynecology and Obstetrics. 2016;132(3):305-8. doi: 10.1016/j.ijgo.2015.07.030.

46. Ahmed MR, Shaaban MM, Meky HK, Amin Arafa ME, Mohamed TY, Gharib WF, et al. Psychological impact of female genital mutilation among adolescent Egyptian girls: A cross-sectional study. The European Journal of Contraception and Reproductive Health Care. 2017;22(4):280-5. doi: 10.1080/13625187.2017.1355454. PubMed PMID: 2017-41705-007.

47. Rasheed SM, Abd-Ellah AH, Yousef FM. Female genital mutilation in Upper Egypt in the new millennium. International Journal of Gynaecology & Obstetrics. 2011;114(1):47-50. PubMed PMID: 21513937.

48. Abolfotouh SM, Ebrahim AZ, Abolfotouh MA. Awareness and predictors of female genital mutilation/cutting among young health advocates. International Journal of Women's Health. 2015;7:259-69. doi: 10.2147/IJWH.S78664.

49. Elbendary RN, Shokry DA, Deeb WS, Morsi EM. Female genital mutilation (FGM): Is it still an existing problem in Egypt? Forensic SciInt. 2021;318:5. doi: 10.1016/j.forsciint.2020.110574. PubMed PMID: WOS:000609011200020.

50. Galal AF, El Gelany S, Goma K. Female genital mutilation (FGM) in Egypt, knowledge and concepts of Egyptian medical students: a cross-sectional study. Journal of Obstetrics and Gynaecology 2022. doi: 10.1080/01443615.2022.2035335. PubMed Central PMCID: PMC WOS:000765594700001.

51. Dehghankhalili M, Fallahi S, Mahmudi F, Ghaffarpasand F, Shahrzad ME, Taghavi M, et al. Epidemiology, Regional Characteristics, Knowledge, and Attitude Toward Female Genital Mutilation/Cutting in Southern Iran. Journal of Sexual Medicine. 2015;12(7):1577-83. PubMed PMID: 26139452.

52. Al Hinai H. Female Genital Mutilation in the Sultanate of Oman: Orchid Project; 2014. Available from: <https://www.orchidproject.org/wp-content/uploads/2019/02/habiba-al-hinai-female-genital-mutilation-in-the-sultanate-of-oman1.pdf>.

53. Rashid A, Iguchi Y. Female genital cutting in Malaysia: a mixed-methods study. BMJ Open. 2019;9(4):e025078. PubMed PMID: 30940756.

54. Rashid AK, Patil S, Valimalar AS. The Practice Of Female Genital Mutilation Among The Rural Malays In North Malaysia. The Internet Journal of Third World Medicine. 2009;9.

55. Khalid S, Sanip S, Mokhtar RH, Masri MA, Mohd Aris MS, Wan Deraman MR, et al. Knowledge and acceptance on female circumcision. BJOG: An International Journal of Obstetrics and Gynaecology. 2013;120:578. doi: 10.1111/1471-0528.12354.
