# Supplementary material for: The global prevalence of female genital mutilation/cutting: A systematic review and meta-analysis of national, regional, facility, and school-based studies
Source: PLoS Med. 2022 Sep 1;19(9):e1004061. doi: 10.1371/journal.pmed.1004061 (PMC9436112; doi:10.1371/journal.pmed.1004061)
Supplement: S3 Appendix — Table A. Characteristics of studies on migrant populations. *Conflicting numbers within the study. The methods state that this was part of a larger study of 338 women with FGM/C. The results are based on 188 women with FGM/C Type III. †Calculated manually using data available in the report. Table B. Studies reporting FGM/C in migrant populations. *Calculated manually. Table C. Types of FGM/C in migrant populations. *Flesh removed. ‡% of mothers. ¶% of daughters. **Pharaonic (Type III). †Genital area cut without flesh removed or nicked. §Genital area sewn closed. ΔTissue removed and some stitching. #Includes 2% untouched in the proportion. ##Includes 2% none in the proportion. Table D. Characteristics of FGM/C procedure for migrant populations. (DOCX) [file pmed.1004061.s012.docx]

**S3 Appendix.** Studies on Migrant Populations.

**Table A.** Characteristics of Studies on Migrant Populations.

| **Host** | **Author** | **Year** | **Data Collection** | **Sampling Method, Study Design** | **Country of Origin** | **Sub-Region** | **Population Description** | **Age** | **Exam/ Patient report** | **Data Collection Site** | **Total FGM** | **Risk of Bias** |
| --- | --- | --- | --- | --- | --- | --- | --- | --- | --- | --- | --- | --- |
| **EUR** | | | | | | | | | | | | |
| **Belgium, Denmark, UK, France** | Leye[1] | 2018 | NA | Purposive, Case series | Unclear | NA | Involved in court cases | 15-49 | NA | NA | Belgium- 21, Denmark- 2, France- 30, UK- 179 | Mod. |
| **Finland** | Koukkula[2] | 2016 | 2010-2012 | Stratified random sampling, Cross Sectional | Somalia (43%), Kurdistan (57%) | Helsinki, Espoo, Vantaa, Turku, Tampere, Vaasa | Migrants | 18-64 | Patient Report | Home, Clinic, Other | 186 | Mod. |
| **Germany** | Loucas[3] | 2017 | 2015-2016 | Purposive, Cross Sectional | Syria (33.6%), Egypt (16.5%), Kosovo (6.9%), Albania (6.7%), Somalia (4.6%), Eritrea (4.8%), Serbia (3.7%), Afghanistan (23.2%) | Mainz, Ingelheim | Migrants | 0.5-18 | Patient  Report | Refugee Centers | 23 | High |
|  | Zinka[4] | 2018 | 2017 | Convenience, Retrospective evaluation of the FGM/C reports, Cross-sectional | Nigeria (n=109), Somalia (n = 27), Sierra Leone (n=6), Eritrea (n=6), Ethiopia (n=4), Tanzania (n=1) | Munich | Asylum seekers | 3 weeks – 45 years | Exam | Community / aid organizations | Women: 45 | Mod. |
|  |  |  |  |  |  |  |  |  |  |  | Girls: 7 |  |
|  | Koschollek[5] | 2020 | 2015-2016 | Convenience, Cross Sectional | Sub-Saharan Africa | Munich, Rhine-Ruhr region, Cologne, Berlin, Frankfurt am Main, Hanover | Migrants | 18-45+ | Patient Report | Public Places | 281 | Mod. |
|  | Hänselmann[6] | 2011 | 2008-2009 | Purposive, Case series | Nigeria, Burkina Faso, Eritrea (43.5%), Ghana, Sudan, Somalia (26.9%) | Nationwide with a focus on the Rhine-Neckar Area | Migrants | 19-59 | Patient report | Hospital/ Clinic  (multiple) | 24 participated (out of 37) | High. |
| **Italy** | Castagna[7] | 2018 | 2007- 2016 | Database of women who have had trauma, retrospective database analysis of migrant women with trauma | Nigeria (n=64), Democratic Republic of Congo (n=24),, Ivory Coast (n=23), Cameroon (n=13), Somalia (n=4), Guinea (n=2), Ethiopia (n=2), Gambia (n=1), Eritrea (n=1), Gabon (n=1), Mali (n=1) | Turin | Migrants | 14-48 | Exam | Rape center | 17 | Mod. |
| **Malta** | Padovese[8] | 2013 | 2010-2011 | Medical records of migrants attending clinic, Cross Sectional | Somalia (70.1%), Eritrea (10.7%), Sudan (4%), Ethiopia (3.7%), Mali (3.1%), Ivory Coast (2.1%), Nigeria (2.1%), Chad (0.5%), Burkina Faso (0.5%), Niger (0,5%), Others (2.7%) | NA | Asylum Seekers | 0-46 (not only FGM/C) | Both | Hospital/ Clinic | 163 | Mod. |
| **Netherlands** | Korfker[9] | 2012 | 2009 | National survey of all midwifery practices, Cross sectional | Burkina Faso, Ivory Coast, Djibouti, Egypt, Eritrea, Ethiopia, Gambia, Guinea Bissau, Liberia, Mali, Mauretania, Sierra Leone, Somalia, Sudan, Chad | NA | Migrants | NA | Exam | Hospital/ Clinic  (All midwifery practices in the country) | 470 | Mod. |
|  | Kawous[10] | 2020 | 2018 | National survey of all midwifery practices, Cross sectional |  | NA | Women who gave birth in a primary care midwifery practice | Reproductive age | Exam | Hospitals/Clinics | 523 | Low |
| **Norway** | Taraldsen[11] | 2021 | 2004-2015 | All possible cases from hospital records, Case series | Somalia (74%), Eritrea (10%), Ethiopia (4.5%), Other/Not Stated (11.5%) | NA | All women who had been examined for an FGM/C related issue at an outpatient clinic | Median 26 years | Exam | Hospital Database | 891 | Low |
|  | Mbanya[12] | 2018 | 2014 | Respondent driven sampling, Cross Sectional | Somalia | Oslo | Migrants | 16-25 | Patient Report | Community based | 82 | Mod. |
| **Portugal** | Division of Sexual, Reproductive, Child and Youth Health; Division of Health Statistics and Monitoring; Shared Services Ministry of Health (SPMS)[13] | 2018 | 2014-2017 | Electronic Health Records in Portugal, Case series | The Republic of Guinea-Bissau; Senegal; Nigeria; Gambia; Ivory Coast; Eritrea; Somalia; Benin; Egypt; Ghana; Sierra Leone | Lisbon and Tagus Valley Health Region | Women who attended hospital with FGM/C | 14-64 | Exam | The Electronic Health Record (RSE) | 237 | Low |
| **Greece** | Vrachnis[14] | 2012 | 2009 | Purposive, Case series | Ethiopia, Eritrea, Egypt, Somalia | Athens | Migrants | 19-31 | Both | Hospital/ Clinic | 7 | Low |
| **Switzerland** | Frick[15] | 2021 | 2010-2016 | Hospital database, Case series | Somalia (34%) Eritrea (28%) Burkina Faso (6%) Ethiopia (5%) Guinea (5%) Sudan (5%) Other (16%) | Geneva | Women attending specialised FGM/C clinic | 20-50 | Exam | Hospital/ clinic | 338* | Low |
|  | Cottler-Casanova[16] | 2021 | 2016-2018 | Purposive, Cross sectional | Participants were from 30 FGM/C practicing countries | Geneva, Lausanne, Bern, Zurich | Women and girls admitted to Swiss university hospital | 0-49 years | Exam | Hospital/ clinic | 207 | Mod. |
| **Sweden** | Wahlberg[17] | 2017 | 2015 | Purposive, RCT | Somalia | Gothenburg and Malmö | Migrants | 18+ | Patient Report | Somali organization | 189† | Mod. |
|  | Wahlberg[18] | 2019 | 2015 | Purposive and Snowball, Cross-Sectional | Somalia | Malmo, Gothenburg, Stockholm, Uppsala | Migrants | 18+ | Patient Report | Somali organisation, cafe´s, Swedish courses, mosques. | 270 | Mod. |
| **England** | Hodes [19] | 2016 | 2006-2014 | Hospital clinic, case series of children suspected with FGM/C | 67% from Somalia, % unavailable for Kenya, Ethiopia, Gambia, Zambia, Malaysia | London | Migrants | Children | Exam | Hospital/ Clinic | 27 | Mod. |
|  | Ali[20] | 2020 | 2014-2019 | Hospital records, Case series | Multiple countries (40% Somalia). 96% African, 4% Asian | London | Patients attending clinic | Median 13 years (2-16) | Patient report and/or Exam | Hospital/ Clinic | 55 | Mod. |
|  | Fawcett[21] | 2018 | 2015-2016 | Hospital records, retrospective database analysis |  | Staffordshire | Patients attending emergency department | 23-40 years old | Exam | Hospital/ Clinic | 34 | Mod. |
|  | Creighton[22] | 2016 | 2014 - 2015 | Purposive, Case series | Somalia, Saudi Arabia, Gambia, UK, unknown | Inner London | Migrant children/ children of migrants | 0-18 | Exam | Hospital/ Clinic | 18 | Mod. |
| **Scotland** | Ford[23] | 2018 | 2010-2013 | Hospital electronic database, retrospective analysis of database. | Nigeria, Sudan, The Gambia, Democratic Republic of Congo, Egypt, Iraq, Sierra Leone, Somalia and Tanzania, Malaysia, India, Brunei, United Arab Emirates, Saudi Arabia and England | Lothian | Migrants accessing maternity services | NA | Exam | Hospitals /Clinics | 107 | Mod. |
| **UK + Ireland** | Hodes[24] | 2021 | 2015-2017 | Surveillance system, Case series | Sudan, Somalia, Gambia, and Eritrea, Europe, the Middle East, South-East Asia |  | Children | 0-16, mean 3 | Exam | Hospital/clinic | 103 | Low |
| **France** | Andro [25] | 2010 | 2007-2009 | Hospital based, Case-Control | Mainly from West and North Africa |  | Women attending gynaecological and family planning clinics | <25 - 45+ | Self-report and exam | Clinic | 678 | High |
| **AMR** | | | | | | | | | | | | |
| **USA** | Sudhinaras-et[26] | 2019 | 2017-2013 | Database of refugees attending clinics in California, Retrospective database analysis | South Asia (73.9%), Southeast Asia (7.8%), Africa (7.4%), Latin America and the Caribbean (4.4%), and Europe/Central Asia (6.4%). | California | Refugees | 0-65+ | Patient Report | Refugee clinics | 162 | Mod. |
|  | Akinsulure-Smith[27] | 2016 | 1996-2014 | Clinic database, retrospective database analysis | Guinea, Cameroon, Sierra Leone, Zaire/Democratic Republic of Congo, Cote d’Ivoire, Burkina Faso, Congo, Liberia, Mauritania | New York City | Survivors of Torture | 34.3  Mean  9.7  (SD) | Patient Report | Hospital/ Clinic | 133 | Mod. |
|  | Chu[28] | 2015 | 2014 | Convenience, Cross-sectional | Sierra Leone (67.6%), Guinea (20.6%), Mali (17.6%), Gambia (16.2%) | New York City | Migrants | 18 +, 35.2  (Mean) 13.4  (SD) | Patient Report | Community | 46 | Mod. |
|  | Akinsulure-Smith[29] | 2014 | NA | Purposive, Cross-Sectional | Sierre Leone (48%) and Liberia (52%) | New York City | Migrants | 20-57 | Patient Report | Community site | 7 | Mod. |
|  | Geynisman-Tan[30] | 2019 | NA | Convenience, Case series | Unclear | New York, Boston, Chicago, Minneapolis, San Francisco | All but one were migrants | 24-40 | Patient Report | Online | 30 | Mod. |
|  | Johnson-Agbakwu[31] | 2022 | 2017 | Community-based purposive snowball sample, Cross-sectional | Somalia | Phoenix and Tucson, Arizona | Somali migrants and refugees | 15+ years | Patient Report | Community | 687 | Mod. |
|  | Wikholm[32] | 2020 | 1996-2020 | Convenience sample of records from a clinic, Case series | Countries with high FGM/C prevalence | New York | Asylum seekers | 18+ years | Both | Hospital/Clinic | 100 | High |
|  | Michlig[33] | 2021 | 2017 | Purposive snowball sampling, Cross sectional | Somaliland | Phoenix and Tucson, Arizona | Bantu Somali | 15+ years | Patient report | Community, health needs assessment survey | 680 | Mod. |
|  | Ukoha[34] | 2015 | NA | Convenience, Cross Sectional | Nigeria | DFW, Texas | Migrants,  Igbo (100%) | 19-55 | Patient Report | Online | Mothers: 64 | Mod. |
|  |  |  |  |  |  |  |  |  |  |  | Girls: 21 |  |
| **WPR** | | | | | | | | | | | | |
| **Australia** | Zurynski[35] | 2017 | 2014 | Purposive, Case series | Kenya, Sudan, Australia, Eritrea, Ethiopia, Sierra Leone, Somalia, East Africa | NA | Refugees, Migrants | 0-18 | Exam | Online | 59 | Mod. |
|  | Varol[36] | 2016 | 2006-2012 | Hospital Electronic Records, Retrospective database analysis | Tanzania, Burundi, Rwanda, Uganda, Sudan, Ethiopia, Eritrea, Djibouti, Somalia, and Kenya, Sierra Leone, Liberia, Guinea, Nigeria and the Democratic Republic of Congo, Egypt, Iran, Iraq, Saudi Arabia and Yemen, Pakistan, Sri Lanka, Indonesia and Singapore. | Unnamed Region | Pregnant Migrants | 15-40+ | Exam | Hospital/ Clinic | 196 | Mod. |
|  | Gibson-Helm[37] | 2014 | 2002–2011 | Entire Database, Case series | North Africa, Middle and East Africa, West Africa | NA | Migrants/ refugees at pregnancy clinic | <20 to over 35 | Exam | Hospital/ Clinic | 78 | High |
|  | Davis[38] | 2019 | 2011-2015 | Entire Database, Retrospective database analysis | Somalia, Sudan, Sierra Leone, Ethiopia, Egypt, Indonesia, Other | Sydney | Women who gave birth | <20 to over 35 | Exam | Hospital/ Clinic | 142 | Mod. |
|  | Shukralla[39] | 2020 | 2014 | Hospital records, Retrospective database analysis | Born in Africa, Malaysia, New Zealand | Western Australia | Women who gave birth | 15-39 years old | Exam | Hospital/ Clinic | 53 | Mod. |
| **EMR** | | | | | | | | | | | | |
| **Saudi Arabia** | Rouzi [40] | 2019 | 2016-2017 | Convenience sample of women attending clinic, Cross-Sectional | Saudi 49.7% Naturalised 13.1% Non Saudi 37.1% | Jeddah | Migrants and nationals | 18- 75 years old | Exam | Hospital/ Clinic | 175 | Mod. |
|  | Rouzi[41] | 2017 | 2014 - 2016 | Convenience sample of women attending clinic, Case-series | Sudan | Jeddah | Migrants | 23-49 years old | Exam | Hospital/ Clinic | 107 | Mod. |
|  | Rouzi[42] | 2017 | 2014 - 2015 | Consecutive convenience, Cross Sectional | Sudan | Jeddah | Migrants | 39.76 (mean) | Patient Report | Hospital/ Clinic | 179 | Mod. |
|  | Malak[43] | 2020 | 2019 | Unclear sampling method, Cross Sectional | Saudi (89.7%) Non-Saudi (10.3%) | NA | Locals & Migrants | 18+ | Patient Report | Online | 50 | Mod. |
| **UAE** | Al Awar[44] | 2020 | 2016-2017 | Purposive, cross sectional | African country, Arab country, Asian country, European country North/South America, Australia, NZ, UAE | Al Ain & Eastern region of Abu Dhabi | Locals & Migrants | 18-50+ years old | Patient Report | Three University Campuses | 344 (mothers) 114 (girls) | Mod. |

^*^Conflicting numbers within the study. The methods state that this was part of a larger study of 338 women with FGM/C. The results are based on 188 women with FGM/C Type III.

^†^Calculated manually using data available in the report

Abbreviations: EMR: Eastern Mediterranean Region. SEAR: South East Asian Region. EUR: European Region. WPR: Western Pacific Region AMR: American Region

**Table B.** Prevalence and studies reporting FGM/C in Migrant Populations.

| **Country** | **Author** | **Year** | **Study Design and Sampling Method** | **Country of Origin** | **Subregion** | **Population description** | **FGM/C (%)** | **Total FGM** | **Sample Size** |
| --- | --- | --- | --- | --- | --- | --- | --- | --- | --- |
| **EMR** | | | | | | | | | |
| **Saudi Arabia** | Malak[43] | 2020 | Unclear sampling method, Cross Sectional | Saudi and Non-Saudi Nationals | NA | Migrants and nationals | 9.4% | 50 | 530 |
|  | Rouzi[40] | 2019 | Convenience sample of women attending clinic, Cross-Sectional | Non-Saudi: Yemen, Sudan, Egypt, Somalia and Ethiopia | Jeddah | Migrants and nationals | 18.2% | 175 | 963 |
|  | Rouzi [42] | 2017 | Consecutive convenience, Cross Sectional | Sudan | Jeddah | Migrants | 67.3% | 179 | 266 |
| **UAE** | Al Awar[44] | 2020 | Purposive, Cross sectional | African country, Arab country, Asian country, European country North/South America, Australia, NZ, UAE | Al Ain and Abu Dhabi | Locals & Migrants | 41.1% (mothers) | 344 (mothers) | 831 (mothers) |
|  |  |  |  |  |  |  | 34.2%* (girls) | 114 (girls) | 333 (girls) |
| **EUR** | | | | | | | | | |
| **Netherlands** | Kawous[10] | 2020 | National survey of all midwifery practices, Cross sectional |  |  | Migrants | 0.54% | 523 | 96932 |
|  | Korfker[9] | 2012 | National survey of all midwifery practices, Cross sectional | Burkina Faso, Ivory Coast, Djibouti, Egypt, Eritrea, Ethiopia, Gambia, Guinea Bissau, Liberia, Mali, Mauretania, Sierra Leone, Somalia, Sudan, Chad | NA | Migrants | 0.32% | 470 | 145,492 |
| **Italy** | Castagna[7] | 2018 | Database of women who have had trauma, retrospective database analysis of migrant women with trauma | Nigeria, Democratic Republic of Congo, Ivory Coast, Cameroon, Somalia, Guinea, Ethiopia, Gambia, Eritrea, Gabon, Mali | Turin | Migrants | 12.5% | 17 | 136 |
| **Malta** | Padovese[8] | 2013 | Hospital records, Cross Sectional | Somalia, Eritrea, Sudan, Ethiopia, Mali, Ivory Coast, Nigeria, Chad, Burkina Faso, Niger, Others | NA | Asylum Seekers | 42.5% | 163 | 384 |
| **England** | Fawcett[21] | 2018 | Hospital records, retrospective database analysis |  | Straffordshire | Refugees/ Migrants | 0.38% | 34 | 8788 |
| **Norway** | Mbanya[12] | 2018 | Respondent driven sampling, Cross Sectional | Somalia | Oslo | Migrants | 51.6% | 82 | 159 |
| **Germany** | Koschollek[5] | 2020 | Convenience, Cross Sectional | Sub-Saharan Africa | Munich, the Rhine-Ruhr region, Cologne, Berlin, Frankfurt am Main, and the region of Hanover | Migrants | 26.9% | 281 | 1,044 |
|  | Loucas[3] | 2017 | Purposive, Cross-Sectional | Syria, Egypt, Kosovo, Albania, Somalia, Eritrea, Serbia, Afghanistan | Mainz, Ingelheim | Refugees | 11% | 23 | 209 |
|  | Zinka[4] | 2018 | Convenience, Cross-sectional | Nigeria, Somalia, Eritrea, Sierra Leone, Ethiopia, Tanzania | Munich | Asylum seekers | Women 67% | 45 | 67 |
|  |  |  |  |  |  |  | Girls 8.14% | 7 | 86 |
| **Sweden** | Wahlberg[18] | 2019 | Purposive and Snowball, Cross-Sectional | Somalia | Gothenburg and Malmö | Migrants | 85% | 270 | 318 |
|  | Wahlberg[17] | 2017 | Purposive, RCT | Somalia | Gothenburg and Malmö | Migrants | 99% | 189† | 191 |
| **Switzerland** | Cottler-Casanova[16] | 2021 | Purposive, Cross-sectional | Participants were from 30 FGM/C practicing countries | Geneva, Lausanne, Bern, Zurich | Migrants | 2.4% | 207 | 8720 |
| **Scotland** | Ford[23] | 2018 | Hospital electronic database, Retrospective analysis of database. | Lothian, originally from: Nigeria, Sudan, The Gambia, Democratic Republic of Congo, Egypt, Iraq, Sierra Leone, Somalia and Tanzania, Malaysia, India, Brunei, United Arab Emirates, Saudi Arabia, England and unknown | Lothian | Migrants attending maternity services | 0.24% | 107 | 44,460 |
| **Finland** | Koukkula[2] | 2016 | Stratified random sampling, Cross Sectional | Somalia and Kurdistan | Helsinki, Espoo, Vantaa, Turku, Tampere, Vaasa | Migrants | 47.81% | 186 | 389 |
| **AMR** | | | | | | | | | |
| **USA** | Sudhinaraset[26] | 2019 | Database of refugees attending clinics in California, Retrospective database analysis | Africa (145/162 patients), Europe/Central Asia, Latin America and the Caribbean, South Asia | California | Refugees | 2.1% | 162 | 8751 |
|  | Ukoha[34] | 2015 | Convenience, Cross Sectional | Nigeria | DFW metropolitan area in Texas | Migrants – mothers  and daughters | Mothers- 46% | 64 | 139 |
|  |  |  |  |  |  |  | Daughters- 33.3% | 21 | 67 |
|  | Akinsulure-Smith[27] | 2016 | Clinic database, Retrospective database analysis | Guinea, Cameroon, Sierra Leone, Zaire/Democratic Republic of Congo, Cote d’Ivoire, Burkina Faso, Congo, Liberia, Mauritania | New York City | Survivors of Torture | 25.9% | 133 | 514 |
|  | Johnson-Agbakwu[31] | 2022 | Community-based purposive snowball sample, Cross-sectional | Somalia | Phoenix and Tucson, Arizona | Somali migrants and refugees | 79% | 687 | 848 |
|  | Michlig[33] | 2021 | Purposive snowball sampling, Cross sectional | Somaliland | Phoenix and Tucson, Arizona | Refugees/ Migrants | 77.4% | 680 | 879 |
|  | Akinsulure-Smith[29] | 2014 | Purposive, Cross-Sectional | Sierra Leone and Liberia | New York City | Migrants | 30% | 7 | 23 |
|  | Chu[28] | 2015 | Purposive, Cross-sectional | Sierra Leone, Guinea, Mali, and Gambia | New York City | Migrants | 68% | 46 | 68 |
| **WPR** | | | | | | | | | |
| **Australia** | Davis[38] | 2019 | Entire Database, Retrospective database analysis | Somalia, Sudan, Sierra Leone, Ethiopia, Egypt, Indonesia, Other | Sydney | Refugees/ Migrants | 1.64% | 142 | 8622 |
|  | Shukralla[39] | 2020 | Hospital records, Retrospective database analysis | Born in Africa, Malaysia, New Zealand | Western Australia | Migrants | 0.71% | 53 | 7494 |
|  | Varol[36] | 2016 | Hospital Electronic Records, Retrospective database analysis | Tanzania, Burundi, Rwanda, Uganda, Sudan, Ethiopia, Eritrea, Djibouti, Somalia, and Kenya, Sierra Leone, Liberia, Guinea, Nigeria and the Democratic Republic of Congo, Egypt, Iran, Iraq, Saudi Arabia and Yemen, Pakistan, Sri Lanka, Indonesia and Singapore. | NA | Pregnant migrants | 2.3% | 196 | 8552 |
| **Case series** | | | | | | | | | |
| **Saudi Arabia** | Rouzi [41] | 2017 | Convenience sample of women attending clinic, Case-series | Sudan | Jeddah | Migrants | 100% | 107 | 107 |
| **Belgium** | Leye[1] | 2018 | Purposive, Case series | NA | NA | Involved in court cases | 100% | 21 | 21 |
| **Denmark** | Leye[1] | 2018 | Purposive, Case series | NA | NA | Involved in court cases | 100% | 2 | 2 |
| **Croatia** | Leye[1] | 2018 | Purposive, Case series | NA | NA | Involved in court cases | 100% | 179 | 179 |
| **France** | Leye[1] | 2018 | Purposive, Case series | NA | NA | Involved in court cases | 100% | 30 | 30 |
| **Greece** | Vrachnis[14] | 2012 | Purposive, Case series | Ethiopia, Eritrea, Egypt, Somalia | Athens | Migrants | 100% | 7 participated, 11 identified | 7 |
| **Norway** | Taraldsen[11] | 2021 | All possible cases from hospital records, Case series | Somalia (74%), Eritrea (10%), Ethiopia (4.5%), Other/Not Stated (11.5%) | NA | Migrants | 97% | 891 | 913 |
| **Portugal** | Division of Sexual, Reproductive, Child and Youth Health; Division of Health Statistics and Monitoring; Shared Services Ministry of Health (SPMS) [13] | 2018 | Electronic Health Records in Portugal, Case series | The Republic of Guinea-Bissau; Senegal; Nigeria; Gambia; Ivory Coast; Eritrea; Somalia; Benin; Egypt; Ghana; Sierra Leone | Lisbon and Tagus Valley Health Region | Attended hospital with FGM |  | 237 | NA |
| **Switzerland** | Frick[15] | 2021 | Hospital database, Case-series | Somalia (34%) Eritrea (28%) Burkina Faso (6%) Ethiopia (5%) Guinea (5%) Sudan (5%) Other (16%) | Geneva | Migrants | 100% | 338 (188 Type III) | 338 (188 Type III) |
| **Germany** | Hänselmann[6] | 2011 | Purposive, Case series | Nigeria, Burkina Faso, Eritrea (43.5%), Ghana, Sudan, Somalia (26.9%) | Germany, Focus on the Rhine-Neckar region | Migrants |  | 24 participated, 37 identified | 37 |
| **England** | Hodes[19] | 2016 | Hospital clinic, Case series of children suspected with FGM/C | Somalia, Kenya, Ethiopia, Gambia, Zambia, Malaysia | London | Migrants | 57% | 27 | 47 |
|  | Creighton[22] | 2016 | Purposive, Case series | Somalia, Saudi Arabia, Gambia, UK, unknown | Inner London | Migrant children/ children of migrants | 100% | 18 | 38 suspected |
|  | Ali [20] | 2020 | Hospital records, Case Series | Multiple countries (40% Somalia). 96% African, 4% Asian | London | Migrant and British children | 100% | 112 | 112 |
| **UK + Ireland** | Hodes[24] | 2021 | Surveillance system, Case series | Sudan, Somalia, Gambia, and Eritrea, Europe, the Middle East, South-East Asia |  | Migrants | 100% | 103 | 103 |
| **France** | Andro [25] | 2014 | Hospital based, Case-Control | Mainly from West and North Africa |  | Migrants | 28.4% | 678 | 2384 |
| **USA** | Wikholm[32] | 2020 | Convenience from records from a clinic, case series | Countries with high FGM/C prevalence | New York | Asylum seekers | 84% | 100 | 119 |
|  | Geynisman-Tan[30] | 2019 | Convenience, Case series | Unclear | New York, Boston, Chicago, Minneapolis, and San Francisco, USA | All but one were migrants | 100% | 30 | 30 |
| **Australia** | Zurynski[35] | 2017 | Purposive, Case series | Kenya, Sudan, Australia, Eritrea, Ethiopia, Sierra Leone, Somalia, East Africa | NA | Refugees and Migrants | 100% | 59 | 59 |
|  | Gibson-Helm[37] | 2014 | Entire Database, Case series | Africa | NA | Migrants/ refugees at pregnancy clinic | 100% | 78 | 78 |

*Calculated manually. Abbreviations: EMR: Eastern Mediterranean Region. SEAR: South East Asian Region. EUR: European Region. WPR: Western Pacific Region AMR: American Region FGM/C: Female Genital Mutilation/Cutting

**Table C.** Types of FGM/C in Migrant Populations.

| **Author** | **Year** | **Country of Origin** | **Host Country** | **Total FGM** | **Sample Size** | **Type 1 (%)** | **Type 2 (%)** | **Type 1 or 2 (%)** | **Type 3 (%)** | **Type 4 (%)** | **Don't Know/Missing Type (%)** | **Other (%)** |
| --- | --- | --- | --- | --- | --- | --- | --- | --- | --- | --- | --- | --- |
| **EMR** | | | | | | | | | | | | |
| Rouzi[40] | 2020 | Non-Saudi: Yemen, Sudan, Egypt, Somalia and Ethiopia | Saudi Arabia | 175 | 963 |  |  | 21.1%* | 6.3% | 26.3% | 46.3% |  |
| Rouzi[41] | 2017 | Sudan | Saudi Arabia | 107 | 107 | 39% | 25% |  | 36% |  |  |  |
| Al Awar[44] | 2020 | African country, Arab country, Asian country, European country North/South America, Australia, NZ, UAE | UAE | 344^‡^ | 831 | 62.8%^‡^ | 16.6%^‡^ |  | 5%^‡ **^ |  | 1.4%^‡^ | 14.2%^‡^ didn’t want to answer |
|  |  |  |  | 114^¶^ | 333 | 81.6%^¶^ | 18.4%^¶^ |  |  |  |  |  |
| **WPR** | | | | | | | | | | | | |
| Zurynski[35] | 2017 | Kenya, Sudan, Australia, Eritrea, Ethiopia, Sierra Leone, Somalia, East Africa | Australia | 59 | 59 | 38.5% | 19.2% |  | 19.2% | 23.1% | (n=33 missing) |  |
| Varol[36] | 2016 | Tanzania, Burundi, Rwanda, Uganda, Sudan, Ethiopia, Eritrea, Djibouti, Somalia, and Kenya, Sierra Leone, Liberia, Guinea, Nigeria and the Democratic Republic of Congo, Egypt, Iran, Iraq, Saudi Arabia and Yemen, Pakistan, Sri Lanka, Indonesia and Singapore. | Australia | 196 | 196 | 33% | 33% |  | 26% |  |  |  |
| Davis[38] | 2019 | Somalia, Sudan, Sierra Leone, Ethiopia, Egypt, Indonesia, Other | Australia | 142 | 8622 | 21.2% | 24.1% |  | 41.1% |  | 13.6% |  |
| Shukralla[39] | 2020 | Born in Africa, Malaysia, New Zealand | Australia | 53 | 7494 | 30% | 32% |  | 34% | 4% |  |  |
| **AMR** | | | | | | | | | | | | |
| Geynisman-Tan[30] | 2019 | Unclear | USA | 30 | 30 | 40% | 23.3% |  | 23.3% |  | 13.3% |  |
| Chu[28] | 2015 | Sierra Leone, Guinea, Mali, and Gambia | USA | 46 | 68 |  |  | 91.9%^*^ |  | 8.9%^†^ |  |  |
| Ukoha[34] | 2015 | Nigeria | USA | 64 ^‡^ | 139 |  |  | 84.4%^‡*^ | 48.4%^‡§^ | 50.8%^‡†^ |  |  |
|  |  |  |  | 21^¶^ | 67 |  |  | 69.6%^¶*^ | 43.5%^¶§^ | 50%^¶†^ |  |  |
| Johnson-Agbakwu [31] | 2022 | Somalia | USA | 687 | 848 | 32% | 20% |  | 35% |  |  |  |
| Wikholm [32] | 2020 | Asylum seekers from countries with high FGM/C prevalence | USA | 100 | 119 | 4.6% | 84.6% |  | 9.2% |  |  |  |
| Michlig [33] | 2021 | Somaliland | USA | 680 | 797 | 36.9% | 23% |  | 40.1% |  |  |  |
| **EUR** | | | | | | | | | | | | |
| Korfker[9] | 2012 | Burkina Faso, Ivory Coast, Djibouti, Egypt, Eritrea, Ethiopia, Gambia, Guinea Bissau, Liberia, Mali, Mauretania, Sierra Leone, Somalia, Sudan, Chad | Netherlands | 470 | 145,492 |  |  |  | 40% |  | 10% | 50% |
| Kawous[10] | 2020 |  | Netherlands | 523 | 96,932 |  |  |  | 32% |  |  |  |
| Vrachnis[14] | 2012 | Ethiopia, Eritrea, Egypt, Somalia | Greece | 7 | 7 | 1 case | 4 cases |  | 2 cases |  |  |  |
| Zinka[4] | 2018 | Nigeria, Somalia, Eritrea, Sierra Leone, Ethiopia, Tanzania | Germany | Women- 45 | 67 | 18 cases | 30 cases |  | 4 cases |  |  |  |
|  |  |  |  | Girls - 7 | 86 |  |  |  |  |  |  |  |
| Hänselmann[6] | 2011 | Nigeria, Burkina Faso, Eritrea (43.5%), Ghana, Sudan, Somalia (26.9%) | Germany | 24 | NA (223 clinics) | 12.6% | 58.3% |  | 25% | 4.1% |  |  |
| Padovese[8] | 2013 | Somalia, Eritrea, Sudan, Ethiopia, Mali, Ivory Coast, Nigeria, Chad, Burkina Faso, Niger, Others | Malta | 163 | 384 | 15.3% | 4.9% |  | 51.5% | 28.2% |  |  |
| Castagna[7] | 2018 | Nigeria, Democratic Republic of Congo, Ivory Coast, Cameroon, Somalia, Guinea, Ethiopia, Gambia, Eritrea, Gabon, Mali | Italy | 17 | 136 | 8 cases | 7 cases |  | 2 cases |  |  |  |
| Hodes[19] | 2016 | Somalia, Kenya, Ethiopia, Gambia, Zambia, Malaysia | England | 27 | 47 | 7.4% | 29.6% |  | 0% | 40.7% | 22.2% |  |
| Creighton[22] | 2016 | Somalia, Saudi Arabia, Gambia, UK, unknown | England | 18 | 38 (suspected) | 5.6% | 11.1% |  | 22.2% | 61.1% |  |  |
| Ali[20] | 2020 | Multiple countries (40% Somalia) | England | 55 | 148 | 27% | 25% |  | 5% | 24% | 18% |  |
| Hodes[24] | 2021 | Sudan, Somalia, Gambia, and Eritrea, Europe, the Middle East, South-East Asia | UK + Ireland | 103 | 103 |  |  | 58% | 8% | 21% | 13% |  |
| Wahlberg[18]^#^ | 2019 | Somalia | Sweden | 270 | 318 |  |  | 12%^*^ | 66% (25%^∆^ &  41%^§^) | 7% | 13% |  |
| Wahlberg[17]^##^ | 2017 | Somalia | Sweden | 187 | 191 |  |  | 11%^*^ | 83% (32%^∆^ &  51%^§^) | 5% |  |  |
| Division of Sexual, Reproductive, Child and Youth Health; Division of Health Statistics and Monitoring; Shared Services Ministry of Health (SPMS) [13] | 2018 |  | Portugal | 237 |  | 3.4% | 54.9% |  | 41.3% |  |  |  |
| Taraldsen[11] | 2021 | Somalia (74%), Eritrea (10%), Ethiopia (4.5%), Other/Not stated (11.5%) | Norway | 891 | 913 | 7.5% | 7.7% |  | 83.4% |  | 1.3% |  |
| Frick[15] | 2021 | Somalia (34%) Eritrea (28%) Burkina Faso (6%) Ethiopia (5%) Guinea (5%) Sudan (5%) Other (16%) | Switzerland | 338 | 338 |  |  |  | 55.6% |  |  | 44.4% |
| Cottler-Casanova[16] | 2021 | Mainly from Eritrea, Somalia, and Cameroon | Switzerland | 207 | 8927 | 12.6% | 27.5% |  | 44.9% | 1.4% | 13.5% |  |
| Andro[25] | 2014 | Mainly from West and North Africa | France | 678 | 2384 |  |  |  | 2.4% |  | 40% | 57.4% no stitching (Type I, II or IV) |

*Flesh removed ^‡^ % of mothers ^¶^ % of daughters ^**^ Pharaonic (Type III) † genital area cut without flesh removed or nicked ^§^ Genital area sewn closed ^∆^Tissue removed and some stitching. ^#^ Includes 2% untouched in the proportion. ^##^ Includes 2% none in the proportion.

Abbreviations: EMR: Eastern Mediterranean Region; SEAR: South East Asian Region; EUR: European Region; WPR: Western Pacific Region; AMR: American Region; FGM/C: Female Genital Mutilation/Cutting

**Table D.** Characteristics of FGM/C Procedure for Migrant Populations.

|  | **Country** | **Author** | **Year** | **Age at FGM/C** | **Performer of FGM** | **Location of Procedure** |
| --- | --- | --- | --- | --- | --- | --- |
| **EUR** | England | Hodes[19] | 2016 | Years: <1 (14.8%), 1-3 (11.1%), 4-6 (18.5%), 7-9 (29.6%), 10-12 (7.4%), 13 (3.7%), unknown (18.5%) | Traditional (41%), Medically (35%), not described (23%) |  |
|  |  | Ali[20] | 2020 | For cases where a history was available: Median age 6 years (range from a few weeks to 14 years) | Medically (13 cases), Traditional (21 cases) | 48/55 (87%) underwent FGM prior to UK entry |
|  |  | Creighton[22] | 2016 | Mean age 6.8 years (range 7 months– 10 years). | Traditional (38%), Medically (62%) | Clinic (38%). Other: One girl - in her bedroom in London at the age of 10. She was cut along with her sister and two cousins. Type 2 FGM/C was confirmed on examination. Another child - while on a family holiday, consistent with type 4 FGM. One child’s parents took her to Malaysia at the age of 7 months and a prick was made to the clitoris by a practitioner at the local hospital with parental consent. The parents were unaware that this traditional practice constituted FGM. |
|  | Greece | Vrachnis[14] | 2012 | Between 3-8 (range), median 4.9 | Traditional – one patient | Outdoors “in the bush” – one patient |
|  | Germany | Hänselmann[6] | 2011 | Infancy (28.6%), 11-15 years (14.3%) |  |  |
|  | UK + Ireland | Hodes[24] | 2021 | 3 years (range 0-11 years) | 45% health professionals, 36% traditional, 12% relative |  |
|  | Norway | Taraldsen[11] |  | Median 7 years |  |  |
|  | Portugal | Division of Sexual, Reproductive, Child and Youth Health; Division of Health Statistics and Monitoring; Shared Services Ministry of Health (SPMS) [13] |  | 6.6 years mean, 0-37 range |  |  |
| **EMR** | Saudi Arabia | Malak[43] | 2020 | One day to a month (52.0%), two months to a year (18.0%), two to five years (6.0%), and six to 15 years (10.0%), don't remember (14%) |  |  |
|  |  | Rouzi[40] | 2019 | Within one week after birth (57.7%) | Traditional (20%), Medically (58.8%), Other (1.2%), Don't know (20%) | Home (58.9%), Clinic (23.4%), midwife's house (4.6%), other (13.2%) |
|  | UAE | Al Awar[44] | 2020 | Daughters: during infancy (0–1 years) (78.9%)  Mothers: 2-10 years (49.2%) | Daughters: Traditional (13.5%), Medically (84.5%)  Mothers: Traditional (74.4%), Medically (25%), don’t know (0.6%) | Daughters: hospital/ clinic (84.5%)  Mothers: hospital/clinic (25%) |
| **AMR** | USA | Wikholm[32] | 2020 | Average 9 years |  |  |
|  |  | Michlig [33] |  | Mean 7.09 years, range 0-15 |  |  |
|  |  | Geynisman-Tan[30] | 2019 | Between ages 1 week and 16 years, median, 6 years |  |  |
|  |  | Chu[28] | 2015 | Under 1 year old (14.6%), 1 to 5 years old (17.4%), 6 to 10 years old (37%), 11 to 15 years old (17.4%), 15 years old or older (4.3%) | Traditional (90.6%), Medically (9.5%) | Country where the procedure occurred: In home country (95.5%) In other West African country (4.5%) |
|  |  | Ukoha[34] | 2015 |  | Mothers: Traditional (73.5%), Medically Trained (17.2%), 9.4 (don't know/other). Daughters: Traditional (60.9%), Medically Trained (39.1%), |  |
| **WPR** | Australia | Zurynski[35] | 2017 |  |  | Where FGM/C occurred based on country of birth: Sudan: Sudan (3) Malaysia (2) Eritrea (1) Kenya: Kenya (4) Sudan (3) Somalia (2) Eritrea: Eritrea (2) Somalia (1) Australia: Australia (2) Indonesia (1) Sierra Leone: Sierra Leone (2) Uganda: Somalia (1) Somalia: Somalia (1) Egypt: Egypt (1) |
|  |  |  |  |  |  |  |

Abbreviations: EMR: Eastern Mediterranean Region. SEAR: South East Asian Region. EUR: European Region. WPR: Western Pacific Region AMR: American Region FGM/C: Female Genital Mutilation/Cutting

**References**

1. Leye E, Van Baelen L. Estimating prevalence of female genital mutilation in the European Union: Existing evidence and future opportunities to optimise estimations. European Journal of Contraception and Reproductive Health Care. 2016;21:10. doi: 10.3109/13625187.2015.1135897.

2. Koukkula M, Keskimaki I, Koponen P, Molsa M, Klemetti R. Female Genital Mutilation/Cutting among Women of Somali and Kurdish Origin in Finland. Birth. 2016;43(3):240-6. PubMed PMID: 27157533.

3. Loucas M, Loucas R, Muensterer OJ. Surgical Health Needs of Minor Refugees in Germany: A Cross-Sectional Study. European Journal of Pediatric Surgery. 2018;28(1):60-6. doi: 10.1055/s-0037-1604398.

4. Zinka B, Bormann C, Graw M, Ackermann I. Morphologische Befunde nach Verstümmelung des weiblichen Genitales [Anatomical findings after female genital mutilation: Investigations of African asylum-seeking women and young girls]. Gynakologe. 2018;51(5):433-44. doi: 10.1007/s00129-018-4235-3.

5. Koschollek C, Kuehne A, Mullerschon J, Amoah S, Batemona-Abeke H, Dela Bursi T, et al. Knowledge, information needs and behavior regarding HIV and sexually transmitted infections among migrants from sub-Saharan Africa living in Germany: Results of a participatory health research survey. PLoS ONE [Electronic Resource]. 2020;15(1):e0227178. PubMed PMID: 31986162.

6. Hänselmann K, Börsch C, Ikenberg H, Strehlau J, Klug SJ. Female genital mutilation in Germany. Geburtshilfe und Frauenheilkunde. 2011;71(3):205-8. doi: 10.1055/s-0030-1270887.

7. Castagna P, Ricciardelli R, Piazza F, Mattutino G, Pattarino B, Canavese A, et al. Violence against African migrant women living in Turin: clinical and forensic evaluation. International Journal of Legal Medicine. 2018;132(4):1197-204. PubMed PMID: 29308532.

8. Padovese V, Egidi AM, Melillo Fenech T, Podda Connor M, Didero D, Costanzo G, et al. Migration and determinants of health: clinical epidemiological characteristics of migrants in Malta (2010-11). Journal of Public Health. 2014;36(3):368-74. PubMed PMID: 24277779.

9. Korfker DG, Reis R, Rijnders ME, Meijer-van Asperen S, Read L, Sanjuan M, et al. The lower prevalence of female genital mutilation in the Netherlands: a nationwide study in Dutch midwifery practices. International Journal of Public Health. 2012;57(2):413-20. PubMed PMID: 22314540.

10. Kawous R, van den Muijsenbergh M, Geraci D, Hendriks KRM, Ortensi LE, Hilverda F, et al. Estimates of female genital mutilation/cutting in the Netherlands: a comparison between a nationwide survey in midwifery practices and extrapolation-model. BMC Public Health. 2020;20(1):6. doi: 10.1186/s12889-020-09151-0. PubMed PMID: WOS:000548954800016.

11. Taraldsen S, Owe KM, Bodtker AS, Bjorntvedt IW, Midhaugeide B, Sandberg M, et al. Female genital cutting in women living in Norway - consequences and treatment. Tidsskrift for Den Norske Laegeforening. 2021;141(15):1438-44. PubMed PMID: WOS:000714665500010.

12. Mbanya VN, Gele AA, Diaz E, Kumar B. Health care-seeking patterns for female genital mutilation/cutting among young Somalis in Norway. BMC Public Health. 2018;18(1):517. PubMed PMID: 29669570.

13. Division of Sexual R, Child and Youth Health; Division of Health Statistics and Monitoring; Shared Services Ministry of Health (SPMS). Mutilação Genital Feminina Análise dos casos registados na PDS/RSE- PP 2014 - 2017. Government of Portugal, 2018.

14. Vrachnis N, Salakos N, Iavazzo C, Iliodromiti Z, Bakalianou K, Kouiroukidou P, et al. Female genital mutilation in Greece. Clinical & Experimental Obstetrics & Gynecology. 2012;39(3):346-50. PubMed PMID: 23157041.

15. Frick A, Azuaga A, Abdulcadir J. Cervical dysplasia among migrant women with female genital mutilation/cutting type III: A cross-sectional study. Int J Gynecol Obstet. 2021:7. doi: 10.1002/ijgo.13921. PubMed PMID: WOS:000705333500001.

16. Cottler-Casanova S, Horowicz M, Gayet-Ageron A, Abdulcadir J. Female genital mutilation/cutting (FGM/C) coding capacities in Swiss university hospitals using the International Classification of Diseases (ICD). BMC Public Health. 2021;21(1):11. doi: 10.1186/s12889-021-11160-6. PubMed PMID: WOS:000664845500001.

17. Wahlberg A, Johnsdotter S, Selling KE, Kallestal C, Essen B. Baseline data from a planned RCT on attitudes to female genital cutting after migration: when are interventions justified? BMJ Open. 2017;7(8):e017506. PubMed PMID: 28801440.

18. Wahlberg A, Johnsdotter S, Ekholm Selling K, Essen B. Shifting perceptions of female genital cutting in a Swedish migration context. PLoS ONE [Electronic Resource]. 2019;14(12):e0225629. PubMed PMID: 31800614.

19. Hodes D, Armitage A, Robinson K, Creighton SM. Female genital mutilation in children presenting to a London safeguarding clinic: A case series. Archives of Disease in Childhood. 2016;101(3):212-6. doi: 10.1136/archdischild-2015-308243.

20. Ali S, Patel R, Armitage AJ, Learner HI, Creighton SM, Hodes D. Female genital mutilation (FGM) in UK children: a review of a dedicated paediatric service for FGM. Archives of Disease in Childhood. 2020;105(11):1075-8. doi: 10.1136/archdischild-2019-318336. PubMed PMID: WOS:000585987800012.

21. Fawcett RJ, Kernohan G. A retrospective analysis of 34 potentially missed cases of female genital mutilation in the emergency department. Emergency Medicine Journal. 2018;35(10):587-9. doi: 10.1136/emermed-2017-206649.

22. Creighton SM, Dear J, de Campos C, Williams L, Hodes D. Multidisciplinary approach to the management of children with female genital mutilation (FGM) or suspected FGM: service description and case series. BMJ Open. 2016;6(2):e010311. PubMed PMID: 26928027.

23. Ford CM, Darlow K, Massie A, Gorman DR. Using electronic maternity records to estimate female genital mutilation in Lothian from 2010 to 2013. European Journal of Public Health. 2018;28(4):657-61. PubMed PMID: 29596591.

24. Hodes D, O'Donnell NA, Pall K, Leoni M, Lok W, Debelle G, et al. Epidemiological surveillance study of female genital mutilation in the UK. Archives of Disease in Childhood. 2021;106(4):372-6. doi: 10.1136/archdischild-2020-319569. PubMed PMID: WOS:000631880800019.

25. Andro A, Cambois E, Lesclingand M. Long-term consequences of female genital mutilation in a European context: self perceived health of FGM women compared to non-FGM women. Social Science & Medicine. 2014;106:177-84. PubMed PMID: 24565761.

26. Sudhinaraset M, Cabanting N, Ramos M. The health profile of newly-arrived refugee women and girls and the role of region of origin: using a population-based dataset in California between 2013 and 2017. International Journal for Equity in Health. 2019;18(1):158. PubMed PMID: 31619244.

27. Akinsulure-Smith AM, Chu T. Exploring female genital cutting among survivors of torture. Journal of Immigrant and Minority Health. 2017;19(3):769-73. doi: 10.1007/s10903-016-0419-x. PubMed PMID: 2017-18207-031.

28. Chu T, Akinsulure-Smith AM. Health outcomes and attitudes toward female genital cutting in a community-based sample of West African immigrant women from high-prevalence countries in New York City. Journal of Aggression, Maltreatment & Trauma. 2016;25(1):63-83. doi: 10.1080/10926771.2015.1081663. PubMed PMID: 2016-08151-006.

29. Akinsulure-Smith AM. Exploring female genital cutting among West African immigrants. Journal of Immigrant and Minority Health. 2014;16(3):559-61. doi: 10.1007/s10903-012-9763-7. PubMed PMID: 2014-16662-030.

30. Geynisman-Tan J, Milewski A, Dahl C, Collins S, Mueller M, Kenton K, et al. Lower Urinary Tract Symptoms in Women With Female Genital Mutilation. Female Pelvic Medicine & Reconstructive Surgery. 2019;25(2):157-60. PubMed PMID: 30807420.

31. Johnson-Agbakwu CE, Fox KA, Banke-Thomas A, Michlig GJ. Influence of Female Genital Mutilation/Cutting on Health Morbidity, Health Service Utilization and Satisfaction with Care among Somali Women and Teenage Girls in the United States. J Racial Ethn Health Disparities. 2022:9. doi: 10.1007/s40615-022-01266-x. PubMed PMID: WOS:000766061800001.

32. Wikholm K, Mishori R, Ottenheimer D, Korostyshevskiy V, Reingold R, Wikholm C, et al. Female Genital Mutilation/Cutting as Grounds for Asylum Requests in the US: An Analysis of More than 100 Cases. Journal of Immigrant and Minority Health. 2020;22(4):675-81. doi: 10.1007/s10903-020-00994-8. PubMed PMID: WOS:000520809300001.

33. Michlig G, Warren N, Berhe M, Johnson-Agbakwu C. Female Genital Mutilation/Cutting among Somali Women in the US State of Arizona: Evidence of Treatment Access, Health Service Use and Care Experiences. International Journal of Environmental Research and Public Health. 2021;18(7):15. doi: 10.3390/ijerph18073733. PubMed PMID: WOS:000638497400001.

34. Ukoha DE. Female genital mutilation/circumcision: Culture and women sexual health in lgbo women residing in Dallas-Fort Worth, Texas. Walden Dissertations and Doctoral Studies. 1944. <https://scholarworks.waldenu.edu/dissertations/19442016>.

35. Zurynski Y, Phu A, Sureshkumar P, Cherian S, Deverell M, Elliott EJ, et al. Female genital mutilation in children presenting to Australian paediatricians. Archives of Disease in Childhood. 2017;102(6):509-15. PubMed PMID: 28082321.

36. Varol N, Dawson A, Turkmani S, Hall JJ, Nanayakkara S, Jenkins G, et al. Obstetric outcomes for women with female genital mutilation at an Australian hospital, 2006-2012: a descriptive study. BMC Pregnancy & Childbirth. 2016;16(1):328. PubMed PMID: 27793119.

37. Gibson-Helm M, Teede H, Cheng IH, Block A, Knight M, East C, et al. Maternal health and pregnancy outcomes among women of refugee background from african countries. Journal of Paediatrics and Child Health. 2014;50:11.

38. Davis G, Jellins J. Female genital mutilation: Obstetric outcomes in metropolitan Sydney. Australian & New Zealand Journal of Obstetrics & Gynaecology. 2019;59(2):312-6. PubMed PMID: 30734267.

39. Shukralla HK, McGurgan P. Maternity care of women affected by female genital mutilation/cutting: An audit of two Australian hospitals. Women and Birth. 2020;33(4):E326-E31. doi: 10.1016/j.wombi.2019.07.008. PubMed PMID: WOS:000541401900003.

40. Rouzi AA, Berg RC, Alamoudi R, Alzaban F, Sehlo M. Survey on female genital mutilation/cutting in Jeddah, Saudi Arabia. BMJ Open. 2019;9(5):e024684. PubMed PMID: 31154295.

41. Rouzi AA, Berg RC, Sahly N, Alkafy S, Alzaban F, Abduljabbar H. Effects of female genital mutilation/cutting on the sexual function of Sudanese women: a cross-sectional study. American Journal of Obstetrics & Gynecology. 2017;217(1):62.e1-.e6. PubMed PMID: 28267442.

42. Rouzi AA, Sahly N, Sawan D, Mansouri N, Alsenani N, Bahkali N, et al. Attitudes towards female genital mutilation among Sudanese men and women living in Saudi Arabia. Clinical and Experimental Obstetrics and Gynecology. 2017;44(6):875-8. doi: 10.12891/ceog3646.2017.

43. Malak M, Basalem D, Aleiidi S, Helabi N, Almutairi M, Alhamed A. Awareness of Female Genital Mutilation/Cutting Among the General Population in 2019: A Survey-based Study in Saudi Arabia. Cureus. 2020;12(1):e6651. PubMed PMID: 31949997.

44. Al Awar S, Al-Jefout M, Osman N, Balayah Z, Al Kindi N, Ucenic T. Prevalence, knowledge, attitude and practices of female genital mutilation and cutting (FGM/C) among United Arab Emirates population. BMC Women’s Health. 2020;20(1):12. doi: 10.1186/s12905-020-00949-z. PubMed PMID: WOS:000529898100003.
